# Understanding the Propagation Step in a Photoredox Cycloaddition Chain Reaction

**DOI:** 10.1021/acscatal.5c08683

**Published:** 2026-04-09

**Authors:** Annemarie A. Lee, Nicole K. Oo, Henry T. Eaton, Cristabella R. Fortna, Lisa A. Fredin, John R. Swierk

**Affiliations:** † Department of Chemistry, 14787 Binghamton University, Binghamton, New York 13902, United States; ‡ Department of Chemistry, 1687 Lehigh University, Bethlehem, Pennsylvania 18015, United States

**Keywords:** transient absorption spectroscopy, kinetic parameters, quantum yield, photoredox, chain reaction, radical cation

## Abstract

Photoredox catalysis has become increasingly significant in academic and industrial processes, replacing harsh reaction conditions and high temperatures with visible light. Photoredox chain reactions that proceed via dark electron or hole “catalysis” represent an intriguing pathway to achieving high-productivity photoredox reactions. Though photoredox chain reactions are known, our understanding of how to design reactions that exhibit chain behavior remains limited. In particular, a key step in designing a chain reaction is a dark propagation step, where an energetically upconverted electron or hole initiates a subsequent cycle of product generation. This study combines quantum yield (QY) measurements, transient absorption spectroscopy (TAS), electrochemical investigations, kinetic modeling, and computational studies of a ruthenium-catalyzed photoredox chain [4 + 2] cyclization between *trans*-anethole and various dienes to interrogate the kinetics of the propagation step. That data demonstrate that the free energy for the propagation step (Δ*G*_prop_) is the key kinetic descriptor for the reaction. There is a linear relationship between Δ*G*_prop_ and the rate constant for propagation, and Δ*G*_prop_ is a good predictor for the QY of the reaction. In addition, Δ*G*_prop_ correlates with the oxidation potential of the diene, offering a simple molecular predictor of chain behavior.

## Graphical abstract

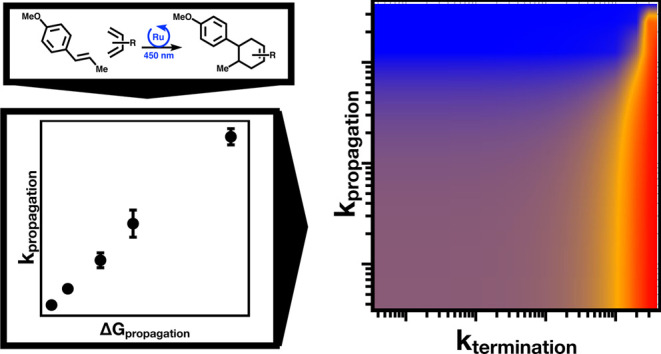

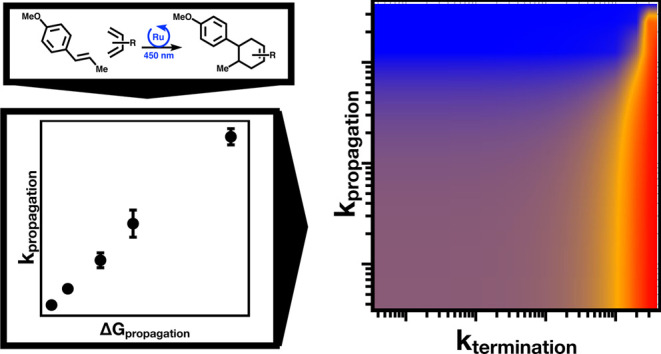

## Introduction

Light-driven synthetic reactions are increasingly valuable tools for small-molecule synthesis. Photoredox reactions rely on photocatalysts that harvest light and undergo photoinduced electron transfer to transform substrates into reactive radical ion intermediates, which can then react further in nonphotochemical steps.^1–4^ The excited states of these photocatalysts are potent oxidants or reductants, while being bench-stable in their ground state, thus avoiding the need for harsh reaction conditions or high temperatures. Additionally, the use of visible light allows for selective activation, as most organic molecules do not absorb in the visible region. This effectively leads to less undesired side-product formation.

While the number of photoredox reactions demonstrated in academic settings has rapidly grown in the last 10+ years, translation to industry has been slower. We recently demonstrated that at an industrial scale, reaction quantum yield (QY, i.e., how much product is made per photon) is a more important reaction parameter than product yield, though QY remains an underreported reaction parameter.^5,6^ Photoredox chain reactions, which produce multiple product molecules per photon, are particularly attractive. Currently, a minority of reactions found in photoredox catalysis are known to operate by a chain mechanism.^7–11^ These reactions can offer effective regio- and chemoselectivity for constructing carbon-heteroatom bonds, including C–C, C–N, C–O, and C–S bonds, via a variety of pathways including cyclization, alkylation, arylation, and aldehyde functionalization.^7,12–15^ Wide substrate scopes have shown the robust nature of photoredox chain reactions and their versatility. The challenge remains, however, in translating milligram-scale reactions to kilogram-scale industrial procedures.

Radical chain reactions, those involving neutral radical species, have been studied and are thoroughly understood in the sense of classical organic chemistry.^16–18^ Some photochemical chain reactions, however, proceed through radical ion intermediates, leading to very different criteria for reactivity and propagation.^7,19^ Generally, photoredox chain reactions start with photoexcitation and charge separation. Single-electron transfer (SET) from the excited state can proceed either through photoinduced oxidation or reduction of the photocatalyst and produces radical ion species.^19^ These species then undergo nonphotoinduced steps. For a chain mechanism to operate, one of these steps must be a propagation step. In the propagation step, a radical ion species sustains the reaction by oxidizing or reducing a substrate molecule, thus restarting the reaction without requiring an additional photon. Essentially, the dark reaction proceeds via hole or electron catalysis, which is how many chain reactions are described. Most commonly, photoredox chain reactions are identified by QY exceeding 1, although Pitre and co-workers recently demonstrated that pulsed illumination can also be effective in identifying photoredox chain reactions.^20^ This efficient use of photon energy reduces photocatalyst loading and enables selective catalytic processes for small-molecule transformations, thereby broadening reaction utility in both academic and industrial settings.

Chabuka and Alabugin have provided a theoretical basis for understanding photoredox chain reactions by describing them as events of electron or hole upconversion.^11^ Using the cyclization of 1,3-butadiene and ethene, Alabugin identified crucial characteristics for a radical ion intermediate to become a more potent oxidizer than its neutral counterpart. This is a requirement for successful electron upconversion, as propagation only occurs when a reaction intermediate is able to oxidize or reduce the starting substrate without additional photon energy. They attribute a radical ion’s effective oxidizing ability to electronic delocalization and hyperconjugative effects. Radical cations are electron-deficient, making them highly reactive in the presence of electron-donating species. Their ability to delocalize the radical ion charge across the molecular structure increases their oxidation potential. Hyperconjugation is responsible for stabilizing the oxidized state of the radical cation and encouraging the reaction to move forward by lowering the activation barrier. Furthermore, electron-withdrawing groups can increase the oxidation potential of the product, leading to even greater oxidizing ability. This last piece of information provides an important consideration when selecting substrates. Experimental support was provided by Chiba and Okada, who analyzed the cycloaddition of anethole and chosen dienes in electrochemical studies.^21^ When comparing anethole to the [4 + 2] cycloaddition product with isoprene, they found that anethole had a lower oxidation potential. Additionally, this held true when reacting anethole with 2,3-dimethyl-1,3-butadiene and 1-phenyl propene. When the oxidation potential of the starting materials was compared to the resulting products, each showed an increase in oxidation potential. Inspired by electrochemical studies such as this one, we were interested in understanding redox upconversion in photocatalyzed reactions, as it can lead to higher product yields and potentially higher QY.

Our previous work focused on the [4 + 2] photoredox cyclization between *trans*-anethole and isoprene, which is catalyzed by a ruthenium bipyrazine photocatalyst ([Ru­(bpz)_3_]^2+^).^22^ Under O_2_, the experimental QY was observed to be ∼40, which agreed well with previous reports.^7,22^ Using transient absorption spectroscopy (TAS), we were able to map out the complete reaction mechanism and kinetics for each step. Briefly, photoexcited [Ru­(bpz)_3_]^2+^ oxidizes *trans*-anethole to generate a radical cation species, which then undergoes a [4 + 2] cyclization with isoprene to generate the product radical cation. The product radical cation can be reduced by the reduced photocatalyst (closed cycle) or can oxidize a new molecule of *trans*-anethole (propagation). Both pathways lead to the neutral product, but chain reactivity was observed when the propagation pathway dominated. Kinetically, this highlighted the competitive nature between the propagation pathway and the chain-terminating reduction step. Use of single-wavelength TAS revealed a rate constant for the propagation pathway of 3.3 (±1.4) × 10^4^ M^–1^ s^–1^, while the reduction of the product radical cation by the reduced photocatalyst had a rate constant of 6.0 (±2.0) × 10^9^ M^–1^ s^–1^. We observed that despite the five orders of magnitude between the rate constants for the two pathways, chain behavior still occurs within the system, albeit at a low level (QY of ∼2). With oxygen present, however, the reduced photocatalyst is oxidized by oxygen to generate superoxide. Reduction of the product radical cation by superoxide has a rate constant an order of magnitude smaller (6.0 (±2.0) × 10^8^ M^–1^ s^–1^) and allows the propagation step to be competitive, thus increasing the QY. In addition, entering the experimental rate constants into a kinetic model allowed us to closely predict the experimental reaction QY. A key observation from the kinetic modeling was how influential the propagation pathway rate constant is on the QY. It demonstrated that as the propagation pathway’s rate constant increases, so does the QY, while altering rate constants of the remaining pathways had a much smaller effect on the overall QY.

Following successful analysis of the *trans-*anethole-isoprene cycloaddition, we hypothesized that a wider range of dienes would allow us to probe the relationship between Δ*G* and the rate of propagation. As previously noted, conversion from starting material to product intermediate raises the oxidation potential, which in turn increases the driving force of the propagation step. We expect that a greater change in the driving force of chain propagation should increase the kinetics of the propagation step, yet no study has examined this relationship experimentally. In this study, we characterize the kinetics of the [4 + 2] cyclization between *trans*-anethole and various dienes (Scheme 1), with a specific interest in the propagation pathway’s rate constant. From here, we can relate the Δ*G* for each unique product to the rate constant for propagation and demonstrate a linear relationship between the two.

**Scheme 1:**
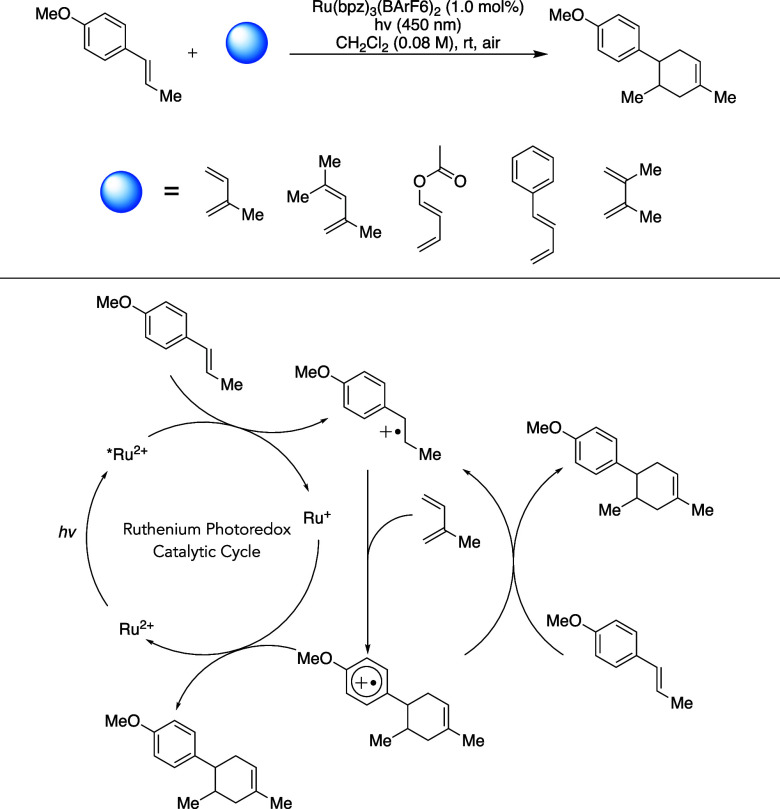
Diels–Alder [4 + 2] Reaction Scheme between Anethole and Multiple Dienes Used to Kinetically Investigate the Propagation Pathway, along with the Originally Proposed Mechanism^7^

## Methods

*trans*-Anethole, 1-acetoxy-1,3-butadiene, dibromomethane, and ruthenium­(III) chloride were purchased from Sigma-Aldrich. Meanwhile 1-phenyl-1,3-butadiene, 2,3-dimethyl-1,3-butadiene, and sodium tetrakis [3,5-bis­(trifluoromethyl)­phenyl]­borate were purchased from Ambeed. Dichloromethane (DCM), ethylene glycol, methanol, acetonitrile, and 2,4-dimethyl-1,3-pentadiene were purchased from Fisher Scientific. Tris­(2,2′-bipyriazyl)­ruthenium­(II) bis­(tetrakis­(3,5-bis­(trifluoromethyl)­phenyl)­borate), also referred to as [Ru­(bpz)_3_]^2+^, was synthesized as previously described by Yoon and co-workers.^23^ All reagents were used as received, unless stated otherwise.

### Quantum Yield Measurements

The reactions for this study were executed using a slightly altered version of Yoon’s reaction conditions.^23^ The external QYs were determined through applying the methodology described in our previous reports.^22,24,25^ *trans*-Anethole (0.19 mmol), [Ru­(bpz)_3_]^2+^ catalyst (1.9 μmol, 1 mol %), and the chosen diene (1.90 mmol, 10 equiv) were added to a 1 cm path-length cuvette. This was followed by 2.4 mL of DCM. The solvent was not purged with nitrogen as these QYs were collected under ambient air. The reaction mixture was set on a stir plate 30 cm away from the 450 nm LED light source (Thorlabs M450LP2). Each reaction was exposed to the LED for a specific amount of time (1–2.5 h) while it was set at a power of 100 μW cm^–2^. Calibration of the LED was performed with a photometer (Thor Laboratories S120C).

The product yield as a function of time was analyzed by using quantitative NMR spectroscopy (Supporting Information). An aliquot of the reaction mixture (25 μL) was dissolved in acetonitrile-d6, along with dibromomethane, as the internal standard, and the resulting ^1^H NMR spectrum was recorded by using a Bruker 400 MHz spectrometer. The external QY was calculated using the equation described in previous publications.^22,24,25^

### Electrochemical Analysis

Spectroelectrochemical experiments were conducted with a BioLogic SP-50 potentiostat, using a platinum honeycomb electrode and a spectroelectrochemical cell with a path length of 1.7 mm (Pine). The potentials were measured relative to a Ag/AgCl reference electrode, and all samples were prepared in a 0.1 M ammonium hexafluorophosphate (NH_4_PF_6_) supporting electrolyte solution with acetonitrile. UV–vis spectra were obtained with a Shimadzu UV-2600 spectrophotometer.

Cyclic voltammograms of the products were obtained using a glassy carbon working electrode and a platinum wire counter electrode, while potentials were measured relative to a Ag/AgCl reference electrode. These samples were also prepared in a 0.1 M NH_4_PF_6_ electrolyte solution, as previously described. Oxidation potentials were determined by fitting the cyclic voltammograms of each cycloaddition product. The fitting of the voltammograms (Supporting Information) and determination of the oxidation potentials were achieved using a cyclic voltammetry simulation method.^26^

### Transient Absorption Spectroscopy

TAS experiments were performed using a Spectra-Physics Quanta-Ray Pro 290 pulsed Nd:YAG laser, in combination with a PrimoScan OPO (optical parametric oscillator). The laser was set to an output power of 2.8 W before the OPO, while the excitation wavelength was set at 430 nm for all experiments. The measured power density of the laser at the location of the sample was found at 20.2 mW/cm^2^. TAS spectra were collected with the Andor Kymera Spectrograph and an Andor iStar CCD (charge-coupled device) camera.

The single-wavelength experiments were performed with the same instrumentation as previously described.^25^ All TAS samples were prepared with 53 mM anethole, 0.50 M of the chosen diene, and 27 μM of the photocatalyst [Ru­(bpz)_3_]^2+^, all dissolved in DCM. When the sample was run under inert conditions, DCM was degassed for 45 min before being added to the cuvette. TAS was performed to determine the propagation rate constant through the same methodology utilized for studying the cyclization reaction between anethole and isoprene.^22^ In a similar manner, samples of anethole and the corresponding diene were analyzed through TAS, and the propagation pathway rate constants were determined accordingly.

Initially, TAS was collected through a CCD camera to get the full TAS spectra within the visible region, covering wavelengths from 375 to 725 nm. Data were collected at delays of 100 ns, 500 ns, 800 ns, and 10 μs. This was followed by single-wavelength experiments that allowed for extraction of kinetic data from a kinetic model. CCD camera experiments were followed by collecting single-wavelength traces, which was performed in 6.4 ns intervals up to 12 μs and then in 1 μs intervals up to 10 ms. Similarly, when testing shorter time traces, data were collected with and without the probe blocked so as to remove residual laser scattering or emission that might be captured. Each anethole and diene pair was investigated using 6–8 different wavelengths from 375 to 610 nm. A new sample was prepared for each wavelength investigated. Following data collection, a detailed kinetic model was used to fit the TAS traces to determine the relative rate constants (Supporting Information)

### Computational Analysis

Energy levels of different cyclohexene products were modeled with density functional theory (DFT) to understand how different substituents can affect the process of “hole” catalysis. The polarizable continuum model^27^ was used to simulate the effect of dichloromethane (CH_2_Cl_2_) as a solvent for both neutral and radical cation species. *trans*-Anethole, a range of substituted dienes, and their cycloadduct products were optimized in Gaussian16 ^28^ using the double-hybrid long-range corrected ωB97XD functional^29^ with a balanced polarized triple-ζ basis set, def2-TZVP,^30,31^ with a super fine integration grid. Each form of product led to numerous conformations that resulted from rotational and steric interactions, which were fully optimized in the ground state. The oxidized states of each reactant and product were optimized after the removal of one electron to form a radical cation. The Gibbs free energies of the lowest-energy ground state (Δ*G* = *G*_left chair_ – [*G*_diene_ + *G*_dienophile_], with energies from Table S11) and the photo-oxidized reaction (Δ*G* ^·+^ = *G*_left chair_^·+^ – [*G*_diene_ + *G*_dienophile_^·+^], with energies from Table S11) are reported. This corresponds to the formation of the left-chair product and the oxidation of the dienophile for all of the reactions. The difference between the ground and radical cation paths was used to quantify the efficiency of “hole” catalysis (Δ*G*_up_ = Δ*G* ^·+^ – Δ*G*). All energies are reported in electron volts (eV). Single-point energies of all compounds were also calculated at the DLPNO-CCSD­(T)^32,33^ level of theory in ORCA 6.1.0 ^34^ with a def2-TZVP orbital basis set^35^ and a def2-TZVP/C auxiliary basis set^36^ under “TIGHTPNO” convergence in vacuum. Single-point energies (Table S12) and reaction favorabilities with upconversion efficiency (Table S13) are provided in the SI. Electronic energies and reaction energies agree well between these two methods.

## Results and Discussion

### Photochemical Studies

For QY studies, the dienophile was fixed as *trans-*anethole, and the diene varied (Scheme 1). All products were previously prepared and characterized by Yoon.^7,37^ It is apparent that the QY varies depending on the diene, with the QY observed for 2,4-dimethyl-1,3-pentadiene reaching a maximum of 68 (Figure 1a). This exceeds the QY when isoprene is used, which has a maximum QY of 43. Meanwhile, the three remaining dienes, including 1-phenyl-1,3-butadiene, 1-acetoxy-1,3-butadiene, and 2,3-dimethyl-1,3-butadiene, all exhibited much lower QYs, ranging from 3 to 6 (Figure 1b). While all anethole–diene pairs exhibited a QY that exceeds 1, thus indicating the presence of chain propagation, the wide range of QYs indicates that the diene influences the productivity of the chain mechanism. In addition, we also observed a slightly higher yield of the anethole dimer in the reaction mixtures with lower QYs (Supporting Information).

**Figure 1:**
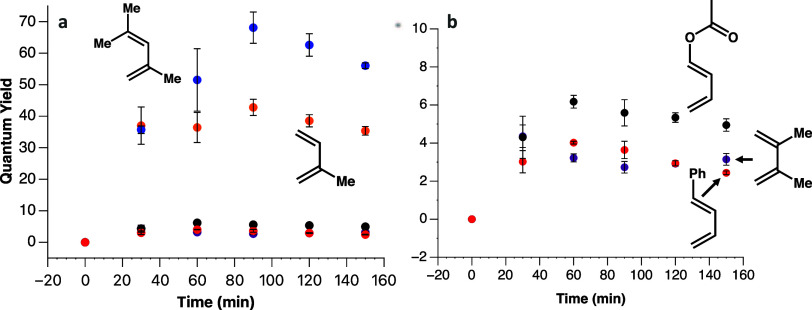
(a) External QY of the [4 + 2] Diels–Alder reaction while irradiated by a 450 nm LED (100 μW cm^–2^) while open to air. Anethole was cyclized with 2,4-diemthyl-1,3-pentadiene (blue), isoprene (orange), 2,3-dimethyl-1,3-butadiene (purple), 1-acetoxy-1,3-butadiene (black), and 1-phenyl-1,3-butadiene (red). (b) External QY of the [4 + 2] Diels–Alder reaction performed with the last three dienes on a smaller scale for clarity.

### Electrochemical Analysis of Oxidation Potentials

Each diene/anethole product was isolated, and the reduction potential for the product radical cation was determined using cyclic voltammetry. In addition to determining the oxidation potentials, we also simulated the cyclic voltammograms to determine the reverse decyclization rate constants. When in the [4 + 2] product radical cation form, decyclization back to the anethole and diene is possible, which could explain a lower product yield. The cyclization products with isoprene and 1-phenyl-1,3-butadiene exhibited the largest rate constants at 100 s^–1^, while the 1-acetoxy-1,3-butadiene product had a decyclization rate constant of 5 s^–1^. The decyclization rate constants for the products with 2,4-dimethyl-1,3-pentadiene (10 s^–1^) and 2,3-dimethyl-1,3-butadiene (50 s^–1^) lie between these extremes.

Contrary to our expectations, there was no obvious trend between decyclization and QY. While the anethole and 2,4-dimethyl-1,3-pentadiene product had a small rate constant and high QY, the smallest decyclization rate constant was for the 1-acetoxy-1,3-butadiene product, which has one of the lowest QYs. When considering the time scale of most of the reaction steps, i.e., nanoseconds to microseconds, it is unsurprising that decyclization of the product radical cation appears to play little, if any, role. Based on the decyclization rate constants, decyclization would be expected to occur on a time scale of 10 ms or longer, which TAS data (vide infra) show is much slower than the rate of electron transfer to reduce the product radical cation.

### Computational Exploration of Hole Upconversion

Each substrate and [4 + 2] product were fully optimized at the ωB97XD/def2-TZVP/PCM­(DCM) level of theory. The ground state of each product is the left-chair isomer (Table S11, Δ*G* in Table 1). The favorability of the reactions ranges from 1.80 to 1.98 eV in the ground state, indicating that the left-chair products are highly stabilized in each case, typically 0.2 eV lower in energy than the right-chair products (Table S11). The excited-state path (Δ*G* ^·+^, Table 1) proceeds through oxidation of the dienophile (Table S11) and then addition to the diene to form the oxidized left-chair product. The diene is 0.28 eV easier to oxidize than 1-phenyl-1,3-butadiene and 0.89 eV more reducing than isoprene. The oxidized reactions are also all favorable, with Gibbs free energies of reaction ranging from 1.46 to 1.64 eV.

**Table 1:** Predicted Favorability of the Lowest-Energy Ground-State (Δ*G*) and Excited-State (Δ*G* ^·+^) Paths for Each Diene and the Predicted “Hole” Catalysis Potential (Δ*G*_up_)­^a^

reagent	Δ*G* (eV)	Δ*G* ^·+^ (eV)	Δ*G*_up_ (eV)
2,4-dimethyl-1,3-pentadiene	–1.80	–1.47	0.32
isoprene	–1.98	–1.65	0.33
2,3-dimethyl-1,3-butadiene	–1.92	–1.60	0.33
1-acetoxy-1,3-butadiene	–1.98	–1.55	0.43
1-phenyl-1,3-butadiene	–1.84	–1.50	0.35

a ωB97XD/def2-TZVP/PCM­(DCM).

Though 4 + 2 cycloadditions are known photodriven reactions, to constitute a chain reaction, propagation must occur after photoexcitation. One such measure of the possibility of propagation is Δ*G*_up_, the difference between the oxidized and neutral Δ*G*. A positive Δ*G*_up_ indicates that the oxidized product is a more powerful oxidizing agent than its ground-state counterpart, allowing the oxidized product to “catalyze” regeneration of the oxidized dienophile as it reduces to the ground-state product. This allows a single photoexcitation to be harnessed to generate multiple products through the lower-energy oxidized mechanism. The oxidized reactions have very similar favorability ordering relative to the ground-state reaction.

Therefore, it is unsurprising that the Δ*G*_up_ for each is ∼0.3 eV, indicating that “hole” catalysis is possible in these systems. These calculated Δ*G*_up_ values agree with experimentally observed QY > 1; however, these energetics do not indicate why isoprene and 2,4-dimethyl-1,3-pentadiene have the highest quantum yields, indicating that reactivity is more complex than this simple picture.

### Transient Absorption Spectroscopy (TAS)

TAS data were collected with samples containing the [Ru­(bpz)_3_]^2+^, anethole, and the chosen diene. Similarly to the TAS collected using anethole and isoprene, absorption features were centered around 365, 495, and 610 nm, while a small bleach consistently appeared around 430 nm (Figures 2 and S20–S23).^22^ From spectroelectrochemical experiments, we assigned the bleach at 430 nm to the reduced ruthenium catalyst with much of the bleach hidden under the transient absorption from the product radical cations. As a result, the bleach at 430 nm is smaller than what the spectroelectrochemical experiment would predict. Generally, all the product intermediates were found to have low absorbance within the range of 550–750 nm, while increasing absorbance was observed within the range of 325–475 nm (Supporting Information). In each combination of diene and anethole, the TAS spectra initially showed the primary formation of the *trans*-anethole radical cation, with consumption at longer delay times. As [4 + 2] cyclization occurs, the bleach at 430 nm become less apparent.

**Figure 2:**
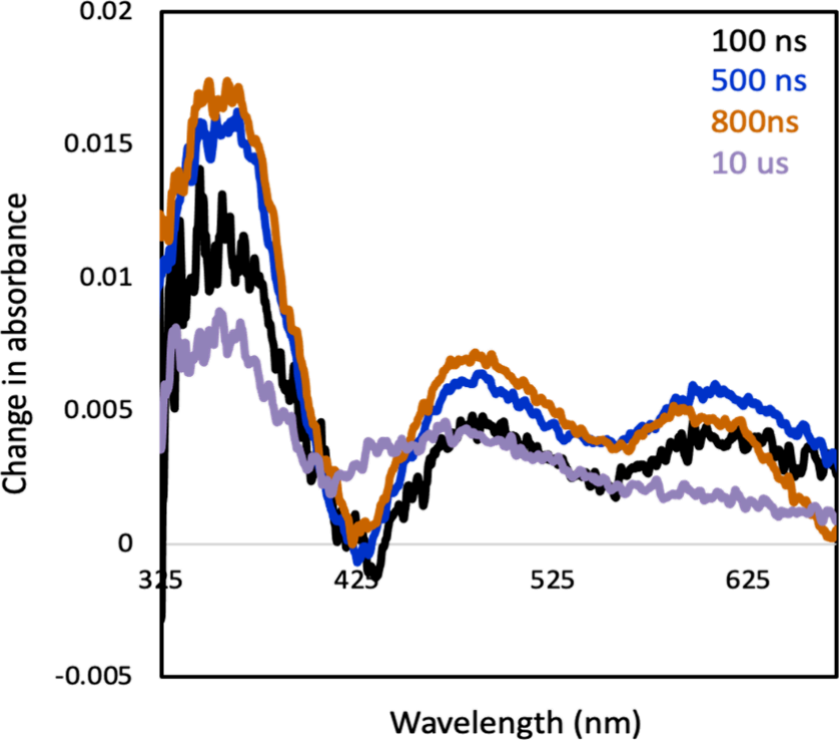
Transient absorption spectrum displaying the changes in absorbance found at delay times ranging from 100 ns to 10 μs after 430 nm excitation of the sample. The samples contained 53 mM anethole, 50 mM 1-phenyl-1,3-butadiene, and 27 μM photocatalyst [Ru­(bpz)­3]­2+ and were open to air.

Following the collection of transient spectra, single-wavelength TAS data were collected. When comparing the single-wavelength traces between different dienes, it was noticeable that the signal had a much longer lifetime for the dienes that displayed greater QY (Figure 3). In order to probe the kinetics of the propagation pathway for each diene, two conditions were studied by TAS: (1) [Ru­(bpz)_3_]^2+^, anethole, and the diene under an inert atmosphere, and (2) [Ru­(bpz)_3_]^2+^, anethole, and the diene under air (Figure 4). Since the only variable in the experiment was the selected diene, we began fitting the traces using rate constants from our previous study of the cyclization of *trans-*anethole and isoprene.^22^ These rate constants included *k*_BET_, *k*_red1[2+2]_, *k*_[2+2]_, *k*_O2_, *k*_BET2_, and *k*_red2[2+2]_. Good-quality fits were achieved using the rate constant values previously determined, though some minor variation within the experimental error was allowed to improve the quality of the fits.

**Figure 3:**
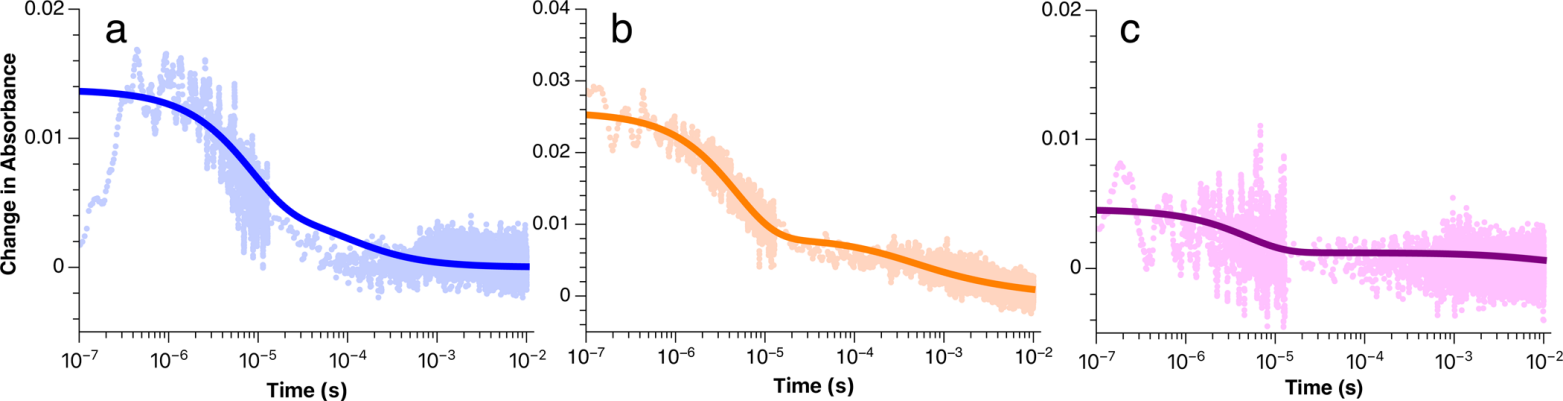
Single-wavelength traces for 27 μM Ru­(bpz)_3_, 53 mM anethole, and (a) 2,4-dimethyl-1,3-pentadiene (50 mM), (b) 2,3 dimethyl-1,3-butadiene (0.5 M), and (c) 1-phenyl-1,3-butadiene (50 mM) at 390 nm under an inert atmosphere.

**Figure 4:**
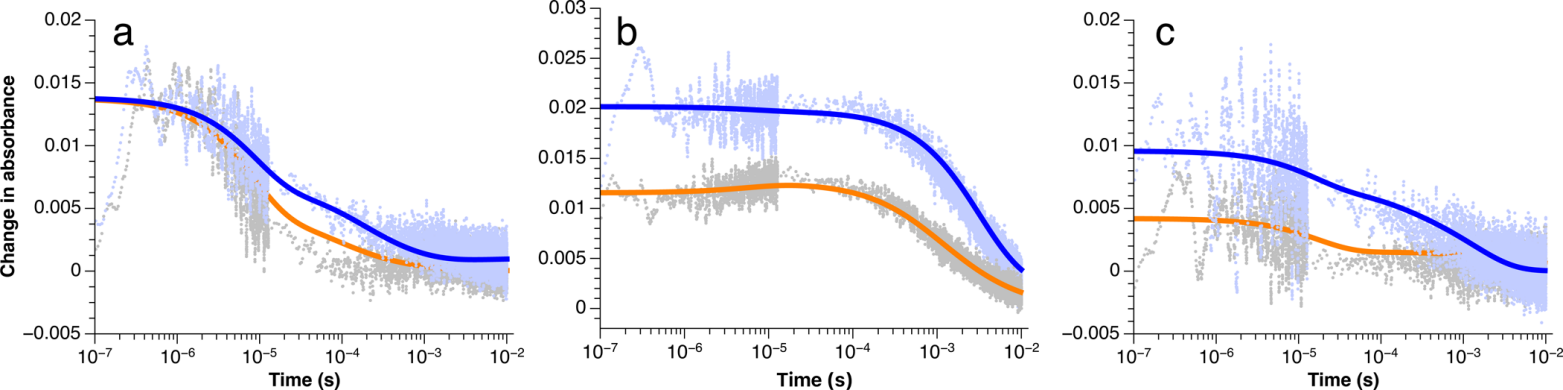
Single-wavelength traces traces for 27 μM Ru­(bpz)_3_, 53 mM anethole, and (a) 2,4-dimethyl-1,3-pentadiene (50 mM at 375 nm), (b) 2,3 dimethyl-1,3-butadiene (0.5 M at 500 nm), and (c) 1-phenyl-1,3-butadiene (50 mM at 390 nm) under an inert atmosphere (orange) and open to air (blue).

After the rate constants for pathways involving [Ru­(bpz)_3_]^2+^, *trans-*anethole, and the diene were determined under an inert atmosphere (*k*_red1[4+2]_, *k*_BET2_ and *k*_prop_), the remaining constant (*k*_red2[4+2]_) was determined by collecting TAS data in air. This allowed us to minimize the number of rate constants determined from fitting any given TAS trace. The inclusion of the propagation pathway in the absence of oxygen was because the reaction exhibited small amounts of chain behavior in the absence of air.^22^ Fits of the TAS data in air used the values of *k*_prop_ determined under N_2_ to confirm that the values were correct.

Rate constants determined for the propagation pathway, *k*_prop_, for each of the dienes agreed with their respective QYs. The cyclization using 2,4-dimethyl-1,3-pentadiene (QY_max_ = 68) had the largest value for *k*_prop_ at 6.5 (±0.2) × 10^4^ M^–1^ s^–1^, which is larger than the value of *k*_prop_ (3.3 (±0.5) × 10^4^ M^–1^ s^–1^) for the reaction with isoprene. The dienes with smaller QY values also exhibited lower values of *k*_prop_. 1-Phenyl-1,3-butadiene as the diene (QY_max_ = 3.0) had a propagation rate constant of 3.2 (±0.4) × 10^3^ M^–1^ s^–1^. The rate constants for the remaining dienes, both of which exhibited low QY, were 1.9 (±0.3) × 10^4^ M^–1^ s^–1^ for 1-acetoxy-1,3-butadiene and 9.3 (±0.4) × 10^3^ M^–1^ s^–1^ for 2,3-dimethyl-1,3-butadiene. Overall, the values of *k*_prop_ determined by TAS for the different dienes are in good agreement with our previous work and compare reasonably to rate constants found for propagation pathways in other radical reactions.^38,39^ In the polymerization of isoprene and trimethyl aminoethyl acrylate, propagation rate constants were determined as 6.8 (±0.7) × 10^4^ and 3.1 (±0.8) × 10^5^ M^–1^ s^–1^, respectively.^39^ Finally, as expected, there is a strong relationship between the propagation rate constant and QY (Figure 5). It appears that this reaction needs a value of *k*_prop_ above 2.0 × 10^4^ M^–1^ s^–1^ to demonstrate significant chain reactivity.

**Figure 5:**
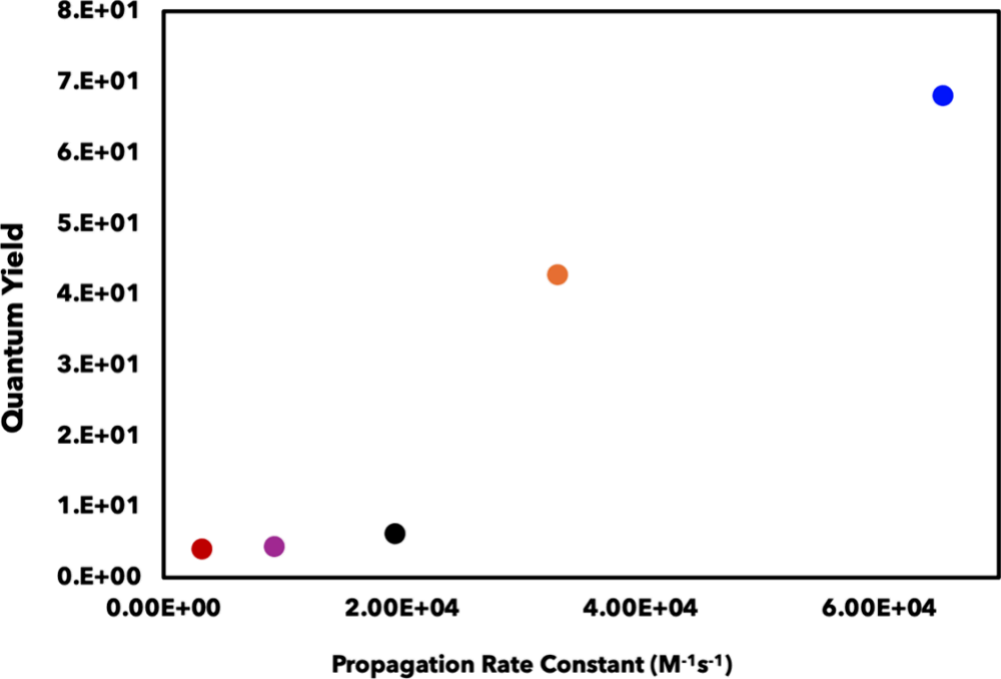
Propagation pathway (*k*_prop_) kinetic rate constants determined for each anethole–diene cyclization in relation to the corresponding reaction QY. The dienes include 2,4-diemthyl-1,3-pentadiene (blue), isoprene (orange), 2,3-dimethyl-1,3-butadiene (purple), 1-acetoxy-1,3-butadiene (black), and 1-phenyl-1,3-butadiene (red).

We also observe a difference in *k*_red1(4+2)_ with different dienes. With isoprene, we previously determined a value of 6.0 (±2.0) × 10^9^ M^–1^ s^–1^, but with the more efficient 2,4-dimethyl-1,3-pentadiene, *k*_red1(4+2)_ decreases to 2.0 (±0.6) × 10^9^ M^–1^ s^–1^.^22^ The slower reduction by the reduced photocatalyst, plus the larger value of *k*_prop_, likely leads to the higher QY. The next two highest-performing dienes, yielding QY_max_ of 6.2 and 4.3, had *k*_red1(4+2)_ rate constants of 1 (±1) × 10^9^ M^–1^ s^–1^ and 2.3 (±0.6) × 10^9^ M^–1^ s^–1^ for 1-acetoxy-1,3-butadiene and 2,3-dimethyl-1,3-butadiene, respectively. These dienes also exhibited much smaller rate constants for the [4 + 2] cyclization. We hypothesize that the slower rate of cyclization prevents significant buildup of product radical cation before [Ru­(bpz)_3_]^+^ is oxidized by O_2_, thus preventing these reactions from benefiting from the slower reduction step. Finally, the 1-phenyl-1,3-butadiene reaction exhibited a large value of *k*_red1(4+2)_ at 3.8 (±0.4) × 10^9^ M^–1^ s^–1^ and the lowest *k*_prop_ at of 3.2 (±0.4) × 10^3^ M^–1^ s^–1^, which helps to explain the low QY_max_ (3.0).

From the TAS and electrochemical data, we are able to determine the relationship between *k*_prop_ and Δ*G*_prop_, the driving force for electron transfer from *trans*-anethole to the different product radical cations. Figure 6 shows a linear correlation between Δ*G*_prop_ and *k*_prop_. As the oxidation potential of the product radical cation increases, the propagation rate constant increases, which demonstrates that the reaction is in the Marcus normal region for electron transfer. Despite being several orders of magnitude smaller than the rate constant for the reduction of the product radical cation by the reduced photocatalyst or superoxide, the large concentration of *trans-*anethole allows the absolute rate of the propagation step to outcompete chain termination. When examining reactions that have less oxidizing product radical cations and thus weakly encourage chain propagation, the absolute rate of propagation is smaller and less competitive with chain termination. This also leads to a strong correlation between QY and Δ*G* (Supporting Information).

**Figure 6:**
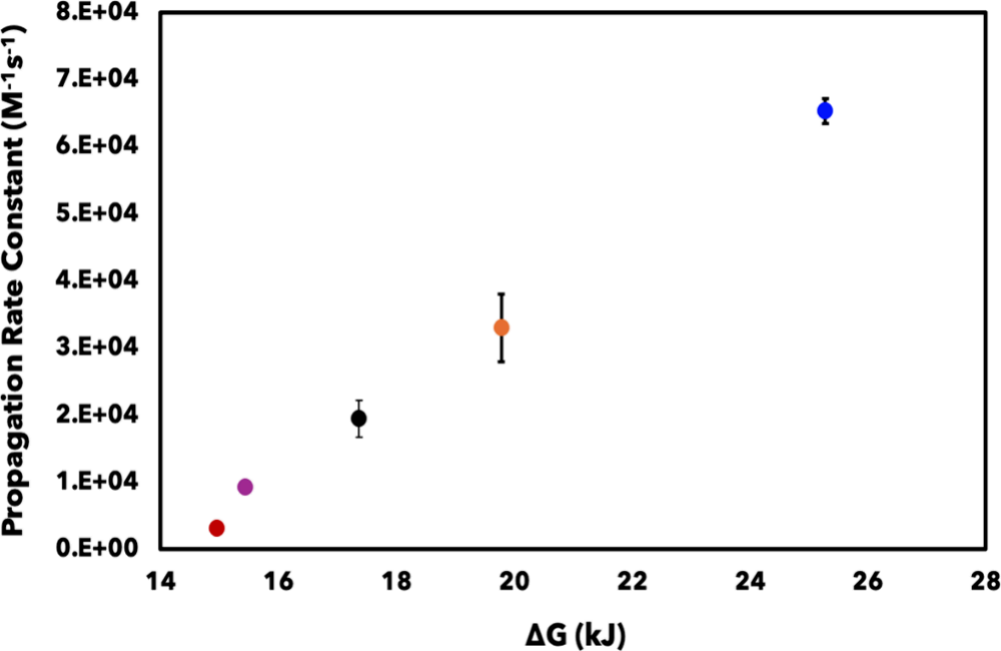
Propagation pathway (*k*_prop_) kinetic rate constants determined for each diene in relation to the change in energy (Δ*G*) of the product intermediate. Each diene has a unique value for *k*_prop_, including 2,4-diemthyl-1,3-pentadiene (blue), isoprene (orange), 2,3-dimethyl-1,3-butadiene (purple), 1-acetoxy-1,3-butadiene (black), and 1-phenyl-1,3-butadiene (red).

### Kinetic Model of the Reaction

Using the rate constants determined from TAS, we used kinetic modeling to predict the behavior of the reaction (Figure S28).^22^ As in our previous report, the predicted QY for anethole and isoprene is slightly lower than that experimentally observed but is in overall good agreement. The predicted QY using 2,4-dimethyl-1,3-pentadiene as the diene was higher than the experimental QY by ∼30% but correctly predicted the increase and then decrease in QY observed experimentally. With the other three dienes, the QY was slightly overestimated than the experimental values, as we saw before when modeling the QY of the anethole/isoprene reaction under N_2_.^22^ Overall, the kinetic modeling is adequate. The difference between experimental and predicted QY is less than an order of magnitude in all cases, and the modeling correctly predicts the order of the QY among the five dienes.

Using product formation between anethole and isoprene as a model for further study, we varied the rates of *k*_prop_ and *k*_red2(4+2)_ (Figure 7, left). The initial rate of product formation over the first 20 min was linear for all combinations of *k*_prop_ and *k*_red2(4+2)_ and is a good proxy for QY. Unsurprisingly, the predicted rate of product formation depends heavily on the value of *k*_prop_ and somewhat less on *k*_red2(4+2)._ Only when the reduction step approaches diffusion-controlled kinetics do we observe a significant drop in the rate.

**Figure 7:**
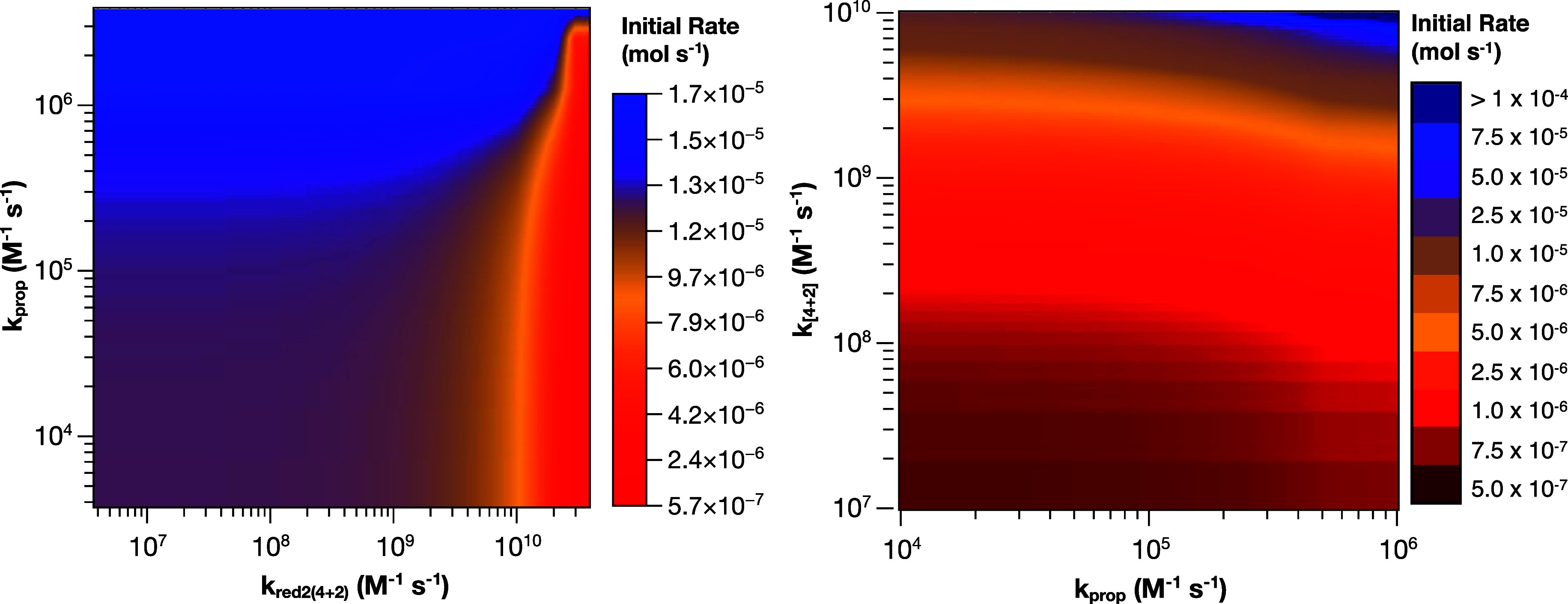
Heatmaps showing the initial rate of 4 + 2 product formation from anethole and isoprene predicted by kinetic modeling as a function of (Left) *k*_prop_ and *k*_red2(4+2)_ and (Right) *k*_prop_ and *k*_[4+2]_. A rate of 5.7 × 10^–7^ mol s^–1^ corresponds to a maximum predicted QY of 1.56, a rate of 1.25 × 10^–5^ mol s^–1^ corresponds to a maximum predicted QY of 35, and a rate of 1.25 × 10^–4^ mol s^–1^ corresponds to a maximum predicted QY of 381.

It is notable that the heatmap between *k*_prop_ and *k*_red2(4+2)_ shows that the reaction appears to have a maximum rate and that further increases in *k*_prop_ will not make a substantial impact. This suggests that another step also plays a role in controlling the QY of the reaction. We hypothesized that the [4 + 2] cyclization step may also be important in controlling QY. To test this, we fixed the value of *k*_red2(4+2)_ at 5 × 10^8^ M^–1^ s^–1^ and varied both *k*_prop_ and *k*_[4+2]_ (Figure 7, right). It is clear from the heatmap between *k*_prop_ and *k*_[4+2]_ that the rate of cyclization between the diene and dienophile plays a key role in determining the QY. Intuitively, this makes sense. A slow cyclization increases the possibility of back electron transfer to the anethole radical cation, which either wastes a photon or terminates the chain. Interestingly, the modeling suggests that if propagation is fast (*k*_prop_ ≥ 10^6^ M^–1^ s^–1^) and the cyclization between the anethole radical cation and diene approaches diffusion-controlled kinetics, then QYs in excess of 1000 are possible for this reaction. Using the same assumptions we previously made when modeling potential productivity with QY,^5^ a reaction with a product yield of 0.9 and a QY of 1000 could potentially produce 155,520 mol of product per day or >33,000 kg of [4 + 2] cycloaddition product. In more practical terms, achieving a QY this high would mean that simple, laboratory-scale flow reactors would be sufficient for photochemistry at scale.

## Conclusions

Designing efficient photoredox chain reactions that operate by hole catalysis requires an understanding of the propagation step. In the case of the photoredox cycloaddition of *trans-*anethole and various dienes, we have demonstrated that there is a linear relationship between Δ*G*_prop_ and *k*_prop_ and that the electron transfer for the propagation step is in the Marcus normal region. In addition, both Δ*G*_prop_ and *k*_prop_ are predictors for QY in the reaction, and if the dienophile in the [4 + 2] cyclization is held constant, the oxidation potentials of the different dienes are a good predictor for Δ*G*_prop_. We also demonstrated computationally that Δ*G*_up_, while a good binary predictor for whether chain reactivity will occur, is a poor predictor for the QY of the reaction. Finally, kinetic modeling shows that *k*_prop_ and *k*_[4+2]_ play a central role in controlling the QY of the chain reaction.

A key finding from the data presented above is the potential impact of increasing *k*_prop_ by increasing Δ*G*_prop_. Larger values of *k*_prop_ lead to higher values of QY and more overall productivity. What is currently unclear, however, is how far Δ*G*_prop_ can be increased. Currently, our data show that the electron transfer of the propagation step is in the Marcus normal region; however, increasing Δ*G*_prop_ could move the reaction into the Marcus inverted region and lead to a decrease in *k*_prop_. In addition, pursuing dienes with higher oxidation potentials may undercut synthetic utility. Thus, it is important to continue exploring the current [4 + 2] photoredox cyclization with a larger range of dienes and dienophiles, as well as other photoredox chain reactions, to understand the limits of Δ*G*_prop_ and *k*_prop_.

Also notable from our data is the importance of *k*_[4+2]_. Experimentally, 2,4-dimethyl-1,3- pentadiene exhibits the largest value of *k*_[4+2]_ and the largest QY. Kinetic modeling further highlights the importance of the cyclization step. Exploration of how the synthetic conditions can modulate and increase the rate of cyclization will likely result in meaningful productivity gains. Further computational exploration may help understand the relative importance of the overall mechanism, transition-state energy, and/or free energy of the cyclization in controlling *k*_[4+2]_.

## Supplementary Material



## Notes

Detailed procedure for QY, single-wavelength traces and fitting, kinetic modeling and computations; NMR data of reactions and [4 + 2] products; cyclic voltammograms and electrochemical simulation fits; TAS spectra and single-wavelength traces and fits; spectroelectrochemical difference spectra; and predicted QY (PDF)
