# Supplementary material for: Understanding the Propagation Step in a Photoredox Cycloaddition Chain Reaction
Source: ACS Catal. 2026 Apr 9;16(8):7367–75. doi: 10.1021/acscatal.5c08683 (PMC13097133; doi:10.1021/acscatal.5c08683)
Supplement: Supplementary file 1 [file cs5c08683_si_001.pdf]

Supporting Information for Understanding the Propagation Step in a Photoredox Cycloaddition Chain Reaction

Annemarie A. Lee,[1] Nicole K. Oo,[2] Henry T. Eaton,[1] Cristabella R. Fortna,[2] Lisa A. Fredin,[2] John R. Swierk[1*]*

1.Department of Chemistry, Binghamton University, Binghamton, NY 13902, United States
2.Department of Chemistry, Lehigh University, Bethlehem, PA 18015, United States

Corresponding Authors: John Swierk (jswierk@binghamton.edu) and Lisa Fredin (laf218@lehigh.edu)

Table of Contents

Procedure for obtaining redox potentials of cyclization products 3

Procedure for fitting TAS singlewavelength traces 3

Table S1. Concentrations determined through fitting difference spectra of reaction intermediates where the $[4+2]^{\bullet+}$ product was synthesized with 2,4-dimethyl-1,3-pentadiene .. 4

Table S2. Concentrations determined through fitting difference spectra of reaction intermediates where the $[4+2]^{\bullet+}$ product was synthesized with 2,3-dimethyl-1,3-butadiene.... 4

Table S3. Concentrations determined through fitting difference spectra of reaction intermediates where the $[4+2]^{\bullet+}$ product was synthesized with 1-acetoxy-1,3-butadiene 4

Table S4. Concentrations determined through fitting difference spectra of reaction intermediates where the $[4+2]^{\bullet+}$ product was synthesized with 1-phenyl-1,3-butadiene 4

Table S5. Rate constants determined from single wavelength traces of $[Ru(bpz)_3]^{2+}$, *trans*-anethole and 2,4-dimethyl-1,3-pentadiene 5

Table S6. Rate constants determined from single wavelength traces of $[Ru(bpz)_3]^{2+}$, *trans*-anethole and 2,3-dimethyl-1,3-butadiene 5

Table S7. Rate constants determined from single wavelength traces of $[Ru(bpz)_3]^{2+}$, *trans*-anethole and 1-acetoxy-1,3-butadiene 6

Table S8. Rate constants determined from single wavelength traces of $[Ru(bpz)_3]^{2+}$, *trans*-anethole and 1-phenyl-1,3-butadiene 6

Figure S1. H1 NMR spectra for reaction quantum yield measurements between anethole and 2,4-dimethyl-1,3-pentadiene and catalyzed by $Ru(bpz)_3^{2+}$. Prominent [4+2] product peaks can be observed at 0.7 ppm and 7.1 ppm. From bottom to top samples are shown from T=0 to 2.5 hr. 7

Figure S2. H1 NMR spectra for reaction quantum yield measurements between anethole and 2,3-dimethyl-1,3-butadiene and catalyzed by $Ru(bpz)_3^{2+}$. Prominent [4+2] product peaks can be observed at 0.7 ppm and 7.1 ppm. From bottom to top samples are shown from T=0 to 2.5 hr. 8

Figure S3. H1 NMR spectra for reaction quantum yield measurements between anethole and 1-acetoxy-1,3-butadiene and catalyzed by $Ru(bpz)_3^{2+}$. Prominent [4+2] product peaks can be observed at 0.7 ppm and 7.1 ppm. From bottom to top samples are shown from T=0 to 2.5 hr. 9

Figure S4. H^1 NMR spectra for reaction quantum yield measurements between anethole and 1-phenyl-1,3-butadiene and catalyzed by $Ru(bpz)_3^{2+}$. Prominent [4+2] product peaks can be

observed at 0.7 ppm and 7.1 ppm. From bottom to top samples are shown from T=0 to 2.5 hr. 10

Figure S5. H1 NMR of isolated [4+2] product synthesized with anethole and 2,4-dimethyl-1,3-pentadiene. 10

Figure S6. H1 NMR of isolated [4+2] product synthesized with anethole and isoprene 11

Figure S7. H1 NMR of isolated [4+2] product synthesized with anethole and 2,3-dimethyl-1,3-butadiene. 11

Figure S8. H1 NMR of isolated [4+2] product synthesized with anethole and 1-acetoxy-1,3-butadiene 12

Figure S9. H1 NMR of isolated [4+2] product synthesized with anethole and 1-phenyl-1,3-butadiene 12

Figure S10. The electrochemically generated difference spectrum for the [4+2] and $[4+2]^{\bullet+}$ product made with anethole and 1-phenyl -1,3-butadiene, in acetonitrile with 0.1M NH_4PF_6. 13

Figure S11. The electrochemically generated difference spectrum for the [4+2] and $[4+2]^{\bullet+}$ product made with anethole and 1-acetoxy-1,3-butadiene, in acetonitrile with 0.1M NH_4PF_6. 13

Figure S12. The electrochemically generated difference spectrum for the [4+2] and $[4+2]^{\bullet+}$ product made with anethole and 2,4-dimethyl-1,3-pentadiene, in acetonitrile with 0.1M NH_4PF_6. 14

Figure S13. The electrochemically generated difference spectrum for the [4+2] and $[4+2]^{\bullet+}$ product made with anethole and 2,3-dimethyl-1,3-butadiene, in acetonitrile with 0.1M NH_4PF_6. 14

Figure S14. The cyclic voltammogram collected for anethole (red) and the fit achieved through electrochemical simulation (blue) to determine oxidation potential 15

Figure S15. The cyclic voltammogram collected for the [4+2] cycloaddition product of anethole and 2,4-dimethyl-1,3-pentadiene (red) and the fit achieved through electrochemical simulation (blue) to determine oxidation potential 15

Figure S16. The cyclic voltammogram collected for the [4+2] cycloaddition product of anethole and isoprene (red) and the fit achieved through electrochemical simulation (blue) to determine oxidation potential 16

Figure S17. The cyclic voltammogram collected for the [4+2] cycloaddition product of anethole and 2,3-dimethyl-1,3-butadiene (red) and the fit achieved through electrochemical simulation (blue) to determine oxidation potential 16

Figure S18. The cyclic voltammogram collected for the [4+2] cycloaddition product of anethole and 1-acetoxy-1,3-butadiene (red) and the fit achieved through electrochemical simulation (blue) to determine oxidation potential 17

Figure S19. The cyclic voltammogram collected for the [4+2] cycloaddition product of anethole and 1-phenyl-1,3-butadiene (red) and the fit achieved through electrochemical simulation (blue) to determine oxidation potential 17

Table S9. Oxidation potentials found for each [4+2] cycloaddition product made by anethole and the specified diene and the calculated ΔG value in kJ 18

Table S10. Constants and values from cyclic volatmmetry simulation to determine the oxidation potentials of *trans*-anethole and each subsequent [4+2] cyclizaton product 18

Computational data 18
Figure S20. Representative left and right half chairs of cyclohexane and the isoprene product, 4'-methoxy-2,4-dimethyl-1,2,3,6-tetrahydro-1,1'-biphenyl, shown for reference 19
Table S11. ωB97XD/def2TZVP/PCM(DCM) Gibbs Free energies of all compounds, including all isomers of cyclized products. .... **Error! Bookmark not defined.**
Figure S21. Transient absorption spectrum displaying the changes in absorbance found at delay times ranging from 100 ns to 10 μs after 430 nm excitation of the sample. The samples contained 53 mM anethole, 50 mM 2,4-dimethyl-1,3-pentadiene and 27 μM of the photocatalyst [Ru(bpz)3]2+ and were open to air. 22
Figure S22. Transient absorption spectrum displaying the changes in absorbance found at delay times ranging from 100 ns to 10 μs after 430 nm excitation of the sample. The samples contained 53 mM anethole, 0.50 M 2,3-dimethyl-1,3-butadiene and 27 μM of the photocatalyst $[Ru(bpz)_3]^{2+}$ and were open to air. 23
Figure S23. Transient absorption spectrum displaying the changes in absorbance found at delay times ranging from 100 ns to 10 μs after 430 nm excitation of the sample. The samples contained 53 mM anethole, 0.50 M 1-acetoxy-1,3-butadiene and 27 μM of the photocatalyst $[Ru(bpz)_3]^{2+}$ and were open to air 23
Figure S24. The 100 ns TAS spectrum for $[Ru(bpz)3]^{2+}$ and anethole excited at 430 nm with the difference spectra obtained through spectro-electrochemical analysis with anethole and 2,4-dimethyl-1,3-pentadiene 24
Figure S25. The 100 ns TAS spectrum for $[Ru(bpz)3]^{2+}$ and anethole excited at 430 nm with the difference spectra obtained through spectro-electrochemical analysis with anethole and 2,3-dimethyl-1,3-butadiene. 24
Figure S26. The 100 ns TAS spectrum for $[Ru(bpz)3]^{2+}$ and anethole excited at 430 nm with the difference spectra obtained through spectro-electrochemical analysis with anethole and 1-acetoxy-1,3-butadiene. 25
Figure S27. The 100 ns TAS spectrum for $[Ru(bpz)3]^{2+}$ and anethole excited at 430 nm with the difference spectra obtained through spectro-electrochemical analysis with anethole and 1-phenyl-1,3-butadiene. 25
Figure S28. Predicted QY from kinetic modelling that utilizes rate constants determined in the transient absorption experiments. 26
References 27

Procedure for obtaining redox potentials of cyclization products
Each diene/anethole product was isolated after complete consumption of anethole. Isolated products were obtained following the procedure from the literature.[1]

Procedure for fitting TAS singlewavelength traces
The single wavelength traces were based on the method used in the previous mechanistic study for the Diels-Alder reaction between anethole and isoprene.[2] The kinetic model was structured similarly with the only difference being [4+2] product changing as the substrates were switched. Changes in absorbance at a given time was determined by:

$$\Delta A(t) = \varepsilon_{an}[An^{\bullet+}]_t + \varepsilon_{Ru(I)}[Ru(I)]_t + \varepsilon_{2+2}[2+2^{\bullet+}]_t + \varepsilon_{4+2}[4+2^{\bullet+}]_t$$

As previously seen the molar extinction coefficient for the anethole radical cation intermediate is ε_{an}, the molar extinction coefficient for the Ruthenium (I) is $\varepsilon_{Ru(I)}$, while ε_{2+2} is the molar extinction coefficient for the anethole dimer radical cation, ε_{4+2} is the molar extinction coefficient for the $[4+2]^{\bullet+}$ intermediate. As in our previous study, $An^{\bullet+}$ represents the anethole radical cation and "diene" refers to selected dienes that were used in each variation of the original anethole and isoprene reaction. Molar extinction coefficients were determined through the difference spectrum for each component. Each unique [4+2] product was synthesized and isolated to find their difference spectrum.

As presented in our previous study, each intermediate's concentration was determined in short intervals of time from 6.4 ns to 1µs, starting from when t=0. The intermediates included Ru(I), $An^{\bullet+}$, $[2+2]^{\bullet+}$, and $[4+2]^{\bullet+}$. Initial concentrations were determined through fitting difference spectra for the afore mentioned intermediates to the 100 ns broadband TAS spectra. Difference spectra previously found for Ru(I), $An^{\bullet+}$, $[2+2]^{\bullet+}$ were applied to the spectral fitting. The following initial concentrations were found for each combination of substrates.

Table S1. Concentrations determined through fitting difference spectra of reaction intermediates where the $[4+2]^{\bullet+}$ product was synthesized with 2,4-dimethyl-1,3-pentadiene

Ru (I)	$An^{\bullet+}$	$[2+2]^{\bullet+}$	$[4+2]^{\bullet+}$
1.75E-06	1.55E-06	1.50E-07	5.00E-08

Table S2. Concentrations determined through fitting difference spectra of reaction intermediates where the $[4+2]^{\bullet+}$ product was synthesized with 2,3-dimethyl-1,3-butadiene

Ru (I)	$An^{\bullet+}$	$[2+2]^{\bullet+}$	$[4+2]^{\bullet+}$
2.48E-06	2.30E-06	9.50E-08	8.50E-08

Table S3. Concentrations determined through fitting difference spectra of reaction intermediates where the $[4+2]^{\bullet+}$ product was synthesized with 1-acetoxy-1,3-butadiene

Ru (I)	$An^{\bullet+}$	$[2+2]^{\bullet+}$	$[4+2]^{\bullet+}$
1.31E-06	1.29E-06	1.82E-07	8.50E-08

Table S4. Concentrations determined through fitting difference spectra of reaction intermediates where the $[4+2]^{\bullet+}$ product was synthesized with 1-phenyl-1,3-butadiene

Ru (I)	$An^{\bullet+}$	$[2+2]^{\bullet+}$	$[4+2]^{\bullet+}$
1.24E-06	1.08E-06	1.30E-07	3.50E-08

Due to the only change in these studies being the diene, while the catalyst and anethole substrate remained unchanged, the rate constants determined for our previous study on the [4+2] cycloaddition were set as the starting point for the reaction pathways that involved anethole, the ruthenium catalyst and O_2. The equations below were used to calculate the concentration of each intermediate species under inert conditions (Tables S1-S4):

$$[An^{\bullet+}]_t = [An^{\bullet+}]_0 - k_{BET}[Ru(I)]_t[An^{\bullet+}]_t - k_{2+2}[An]_t[An^{\bullet+}]_t - k_{4+2}[diene][An^{\bullet+}]_t$$
$$[Ru(I)]_t = [Ru(I)]_0 - k_{BET}[Ru(I)]_t[An^{\bullet+}]_t - k_{red1[2+2]}[Ru(I)]_t[[2+2]^{\bullet+}]_t - k_{red1[4+2]}[Ru(I)]_t[[4+2]^{\bullet+}]_t$$
$$[2+2^{\bullet+}]_t = k_{2+2}[An]_t[An^{\bullet+}]_t - k_{red1[2+2]}[Ru(I)]_t[[2+2]^{\bullet+}]_t$$
$$[4+2^{\bullet+}]_t = k_{4+2}[diene]_t[An^{\bullet+}]_t - k_{red1[4+2]}[Ru(I)]_t[[4+2]^{\bullet+}]_t$$

The equations below were used to calculate the concentration of each intermediate species under non-inert conditions (Tables S1-S4):

$[An^{\bullet+}]_t = [An^{\bullet+}]_0 + k_{prop}[[4+2^{\bullet}]^{+}]_t[An]_t - k_{BET}[Ru(I)]_t[An^{\bullet+}]_t - k_{2+2}[An]_t[An^{\bullet+}]_t - k_{4+2}[diene][An^{\bullet+}]_t - k_{BET2}[An^{\bullet+}]_t[[O_2^{\bullet-}]_t$

$[Ru(I)]_t = [Ru(I)]_0 - k_{BET}[Ru(I)]_t[An^{\bullet+}]_t - k_{red1[2+2]}[Ru(I)]_t[2+2^{\bullet+}]_t - k_{red1[4+2]}[Ru(I)]_t[4+2^{\bullet+}]_t - k_{redO2}[Ru(I)]_t[O_2]_t$

$[2+2^{\bullet+}]_t = k_{2+2}[An]_t[An^{\bullet+}]_t - k_{red1[2+2]}[Ru(I)]_t[[2+2]^{\bullet+}]_t - k_{red2[2+2]}[[2+2]^{\bullet+}]_t[O_2^{\bullet-}]_t$

$[4+2^{\bullet+}]_t = k_{4+2}[diene]_t[An^{\bullet+}]_t - k_{red1[4+2]}[Ru(I)]_t[[4+2]^{\bullet+}]_t - k_{red2[4+2]}[[4+2]^{\bullet+}]_t [[O_2^{\bullet-}]_t - k_{prop}[[4+2]^{\bullet+}]_t[An]_t$

Table S5. Rate constants determined from single wavelength traces of $[Ru(bpz)_3]^{2+}$, *trans*-anethole and 2,4-dimethyl-1,3-pentadiene

No O2		375 nm	380 nm	390 nm	480 nm	490 nm	500 nm	525 nm	610 nm	Average	Error
kBET	M-1 s-1	1.30E+10	1.30E+10	1.30E+10	1.30E+10	1.30E+10	1.30E+10	1.30E+10	1.30E+10	1.30E+10	0.00E+00
kred1[2+2]	M-1 s-1	3.50E+08	3.50E+08	4.00E+08	3.67E+08	3.67E+08	2.90E+08	3.54E+08	3.54E+08	3.54E+08	1.08E+07
k[2+2]	M-1 s-1	2.00E+07	2.00E+07	2.00E+07	2.00E+07	2.00E+07	4.00E+07	2.33E+07	2.33E+07	2.33E+07	2.44E+06
kred1[4+2]	M-1 s-1	2.00E+09	1.00E+09	6.00E+09	1.00E+09	1.00E+09	2.00E+09	2.17E+09	1.00E+09	2.02E+09	5.98E+08
k[4+2]	M-1 s-1	8.00E+08	5.00E+08	8.00E+08	6.00E+08	6.00E+08	6.00E+08	6.00E+08	5.00E+08	6.25E+08	4.12E+07
kprop	M-1 s-1	6.00E+04	8.00E+04	6.00E+04	7.00E+04	6.00E+04	6.00E+04	5.50E+04	5.50E+04	6.25E+04	2.99E+03

With O2		375 nm	380 nm	390 nm	480 nm	490 nm	500 nm	525 nm	610 nm	Average	Error
kBET	M-1 s-1	1.30E+10	1.30E+10	1.30E+10	1.30E+10	1.30E+10	1.30E+10	1.30E+10	1.30E+10	1.30E+10	0.00E+00
kred1[2+2]	M-1 s-1	3.54E+08	3.54E+08	3.54E+08	2.90E+08	2.90E+08	2.90E+08	2.90E+08	2.90E+08	3.14E+08	1.17E+07
k[2+2]	M-1 s-1	2.33E+07	5.00E+07	3.00E+07	5.00E+07	5.00E+07	5.00E+07	5.00E+07	5.00E+07	4.42E+07	3.87E+06
kred1[4+2]	M-1 s-1	2.02E+09	2.02E+09	2.02E+09	2.02E+09	2.02E+09	2.02E+09	2.02E+09	2.02E+09	2.02E+09	0.00E+00
k[4+2]	M-1 s-1	5.00E+08	3.00E+08	6.00E+08	6.00E+08	6.00E+08	6.00E+08	6.00E+08	3.00E+08	5.13E+08	4.79E+07
kredO2	M-1 s-1	3.00E+09	8.00E+09	8.00E+09	8.00E+09	8.00E+09	8.00E+09	8.00E+09	8.00E+09	7.38E+09	6.25E+08
kBET2	M-1 s-1	6.40E+08	6.40E+08	6.40E+08	6.40E+08	6.40E+08	6.40E+08	6.40E+08	6.40E+08	6.40E+08	0.00E+00
kRed2[2+2]	M-1 s-1	2.00E+08	8.00E+07	8.00E+07	8.00E+07	8.00E+07	8.00E+07	8.00E+07	8.00E+07	9.50E+07	1.50E+07
kred2[4+2]	M-1 s-1	9.00E+08	3.00E+08	1.00E+08	1.00E+08	1.00E+08	1.00E+08	1.00E+08	1.00E+08	2.25E+08	9.96E+07
kprop	M-1 s-1	6.25E+04	7.00E+04	6.00E+04	6.00E+04	7.00E+04	7.00E+04	7.00E+04	6.00E+04	6.53E+04	1.80E+03

Table S6. Rate constants determined from single wavelength traces of $[Ru(bpz)_3]^{2+}$, *trans*-anethole and 2,3-dimethyl-1,3-butadiene

No O2		375 nm	380 nm	390 nm	490 nm	500 nm	525 nm	610 nm	Average	Error
kBET	M-1 s-1	1.30E+10	1.30E+10	1.30E+10	1.30E+10	1.30E+10	1.31E+10	1.31E+10	1.30E+10	1.84E+07
kred1[2+2]	M-1 s-1	3.50E+08	2.90E+08	2.90E+08	3.10E+08	3.10E+08	2.00E+08	2.00E+08	2.79E+08	2.16E+07
k[2+2]	M-1 s-1	3.00E+07	8.00E+07	5.00E+07	6.00E+07	8.00E+07	4.00E+07	4.00E+07	5.43E+07	7.51E+06
kred1[4+2]	M-1 s-1	5.00E+09	2.00E+09	2.00E+09	3.00E+09	3.00E+09	6.00E+08	6.00E+08	2.31E+09	5.82E+08
k[4+2]	M-1 s-1	2.00E+08	1.00E+08	1.00E+08	8.00E+07	1.00E+08	2.90E+07	2.90E+07	9.11E+07	2.18E+07
kprop	M-1 s-1	4.00E+03	1.00E+04	1.00E+04	9.00E+03	9.00E+03	1.00E+04	1.00E+04	8.86E+03	8.29E+02

With O2		375 nm	380 nm	390 nm	480 nm	490 nm	500 nm	525 nm	610 nm	Average	Error
kBET	M-1 s-1	1.30E+10	1.30E+10	1.30E+10	1.30E+10	1.30E+10	1.30E+10	1.30E+10	1.30E+10	1.30E+10	0.00E+00
kred1[2+2]	M-1 s-1	2.79E+08	2.79E+08	2.79E+08	2.79E+08	2.79E+08	2.79E+08	2.79E+08	2.79E+08	2.79E+08	0.00E+00
k[2+2]	M-1 s-1	4.00E+07	4.00E+07	4.00E+07	6.00E+07	7.00E+07	7.00E+07	2.50E+07	8.00E+07	5.31E+07	6.88E+06
kred1[4+2]	M-1 s-1	2.31E+09	2.31E+09	2.31E+09	2.31E+09	2.31E+09	2.31E+09	2.31E+09	2.31E+09	2.31E+09	0.00E+00
k[4+2]	M-1 s-1	3.00E+08	1.50E+08	9.00E+07	7.00E+07	7.00E+07	7.00E+07	2.00E+08	6.00E+07	1.26E+08	3.03E+07
kredO2	M-1 s-1	3.00E+09	3.00E+09	3.00E+09	3.00E+09	3.00E+09	3.00E+09	8.00E+09	3.71E+09	3.71E+09	6.19E+08
kBET2	M-1 s-1	6.40E+08	6.40E+08	6.40E+08	6.40E+08	6.40E+08	6.40E+08	6.40E+08	6.40E+08	6.40E+08	0.00E+00
kRed2[2+2]	M-1 s-1	3.00E+08	3.00E+08	3.00E+08	1.00E+08	1.00E+08	8.00E+07	8.00E+07	1.80E+08	1.80E+08	3.68E+07
kred2[4+2]	M-1 s-1	5.00E+08	5.00E+08	3.00E+08	2.00E+08	2.00E+08	2.00E+08	2.00E+08	1.00E+08	2.75E+08	5.26E+07
kprop	M-1 s-1	8.86E+03	8.86E+03	1.00E+04	1.00E+04	1.00E+04	1.00E+04	1.00E+04	9.27E+03	9.27E+03	3.97E+02

Table S7. Rate constants determined from single wavelength traces of $[Ru(bpz)_3]^{2+}$, *trans*-anethole and 1-acetoxy-1,3-butadiene

No O2		375 nm	380 nm	390 nm	480 nm	490 nm	500 nm	525 nm	610 nm	Average	Error
kBET	M-1 s-1	1.30E+10	1.30E+10	1.31E+10	1.30E+10	1.30E+10	1.30E+10	1.30E+10	1.30E+10	1.30E+10	1.25E+07
kred1[2+2]	M-1 s-1	2.90E+08	2.50E+08	2.03E+08	2.03E+08	2.03E+08	2.50E+08	2.50E+08	2.50E+08	2.37E+08	1.11E+07
k[2+2]	M-1 s-1	5.00E+07	3.00E+07	2.49E+07	5.00E+07	5.00E+07	6.00E+07	3.00E+07	5.00E+07	4.31E+07	4.53E+06
kred1[4+2]	M-1 s-1	8.00E+09	9.00E+08	5.20E+08	5.00E+08	3.00E+08	1.00E+08	1.00E+08	1.00E+08	1.32E+09	9.60E+08
k[4+2]	M-1 s-1	8.00E+07	8.00E+07	7.00E+07	2.00E+07	4.00E+07	3.00E+07	8.00E+07	8.00E+07	5.71E+07	9.93E+06
kprop	M-1 s-1	1.00E+04	3.00E+04	1.00E+04	3.00E+04	3.00E+04	2.00E+04	2.00E+04	1.00E+04	2.00E+04	3.27E+03

With O2		375 nm	380 nm	390 nm	480 nm	490 nm	500 nm	550 nm	610 nm	Average	Error
kBET	M-1 s-1	1.30E+10	1.30E+10	1.30E+10	1.30E+10	1.30E+10	1.30E+10	1.3E+10	1.30E+10	1.30E+10	0.00E+00
kred1[2+2]	M-1 s-1	2.90E+08	2.37E+08	2.37E+08	2.90E+08	2.64E+08	2.64E+08	2.64E+08	2.64E+08	2.64E+08	7.08E+06
k[2+2]	M-1 s-1	3.00E+07	4.31E+07	6.00E+07	5.00E+07	2.00E+07	5.00E+07	8.00E+07	3.00E+07	4.54E+07	6.78E+06
kred1[4+2]	M-1 s-1	1.32E+09	1.32E+09	1.32E+09	1.32E+09	1.32E+09	1.32E+09	1.32E+09	1.32E+09	1.32E+09	0.00E+00
k[4+2]	M-1 s-1	1.10E+08	9.00E+07	5.00E+07	6.00E+07	1.20E+08	5.00E+07	2E+08	6.00E+07	9.25E+07	1.81E+07
kredO2	M-1 s-1	3.00E+09	1.00E+10	3.00E+09	3.00E+09	3.00E+09	6.00E+09	5E+09	4.71E+09	4.71E+09	8.60E+08
kBET2	M-1 s-1	6.00E+08	6.00E+08	6.40E+08	6.13E+08	6.40E+08	6.40E+08	6.3E+08	6.40E+08	6.25E+08	6.43E+06
kRed2[2+2]	M-1 s-1	3.00E+08	2.00E+08	3.00E+08	1.00E+08	2.25E+08	1.00E+08	1.3E+08	1.30E+08	1.86E+08	2.95E+07
kred2[4+2]	M-1 s-1	8.00E+08	8.00E+08	7.00E+08	7.67E+08	1.00E+08	1.00E+08	5.45E+08	4.00E+08	5.27E+08	1.05E+08
kprop	M-1 s-1	1.00E+04	1.00E+04	3.00E+04	2.00E+04	3.00E+04	1.50E+04	2.00E+04	2.00E+04	1.94E+04	2.74E+03

Table S8. Rate constants determined from single wavelength traces of $[Ru(bpz)_3]^{2+}$, *trans*-anethole and 1-phenyl-1,3-butadiene

No O2		375 nm	380 nm	390 nm	480 nm	490 nm	525 nm	525 nm	610 nm	Average	Error
kBET	M-1 s-1	1.58E+10	1.58E+10	1.58E+10	1.58E+10	1.30E+10	1.30E+10	1.30E+10	1.30E+10	1.44E+10	5.29E+08
kred1[2+2]	M-1 s-1	5.00E+08	5.00E+08	2.90E+08	2.90E+08	2.90E+08	2.90E+08	2.90E+08	2.90E+08	3.43E+08	3.44E+07
k[2+2]	M-1 s-1	2.00E+07	2.00E+07	5.00E+07	5.00E+07	5.00E+07	5.00E+07	5.00E+07	5.00E+07	4.25E+07	4.91E+06
kred1[4+2]	M-1 s-1	5.00E+09	5.00E+09	5.00E+09	3.00E+09	3.00E+09	3.00E+09	3.00E+09	3.00E+09	3.75E+09	3.66E+08

k[4+2]	M-1 s-1	5.00E+08	5.00E+08	5.00E+08	4.00E+08	4.00E+08	4.00E+08	4.00E+08	4.00E+08	4.38E+08	1.83E+07
kprop	M-1 s-1	4.00E+03	4.00E+03	3.00E+03	1.00E+03	2.00E+03	2.40E+03	3.20E+03	2.60E+03	2.78E+03	3.57E+02

**With O2**		**375 nm**	**380 nm**	**390 nm**	**480 nm**	**490 nm**	**500 nm**	**525 nm**	**610 nm**	**Average**	**Error**
kBET	M-1 s-1	1.30E+10	1.30E+10	1.30E+10	1.30E+10	1.30E+10	1.30E+10	1.30E+10	1.30E+10	1.30E+10	0.00E+00
kred1[2+2]	M-1 s-1	3.43E+08	3.43E+08	3.43E+08	3.43E+08	3.43E+08	2.90E+08	2.90E+08	2.90E+08	3.23E+08	9.70E+06
k[2+2]	M-1 s-1	2.00E+07	2.00E+07	2.00E+07	3.00E+07	3.00E+07	3.00E+07	3.00E+07	3.00E+07	2.63E+07	1.83E+06
kred1[4+2]	M-1 s-1	3.75E+09	3.75E+09	3.75E+09	3.75E+09	3.75E+09	3.75E+09	3.75E+09	3.75E+09	3.75E+09	0.00E+00
k[4+2]	M-1 s-1	6.00E+08	3.00E+08	3.00E+08	5.00E+08	5.00E+08	5.00E+08	5.00E+08	5.00E+08	4.63E+08	3.75E+07
kredO2	M-1 s-1	3.00E+09	3.00E+09	6.00E+09	5.00E+09	5.00E+09	5.00E+09	3.00E+09	3.00E+09	4.13E+09	4.41E+08
kBET2	M-1 s-1	6.40E+08	6.40E+08	6.40E+08	6.40E+08	6.40E+08	6.40E+08	6.40E+08	6.40E+08	6.40E+08	0.00E+00
kRed2[2+2]	M-1 s-1	2.00E+08	1.00E+08	1.00E+08	1.33E+08	1.33E+08	1.00E+08	1.00E+08	1.00E+08	1.21E+08	1.25E+07
kred2[4+2]	M-1 s-1	6.00E+08	4.00E+08	4.00E+08	4.67E+08	2.00E+08	2.00E+08	9.00E+07	9.00E+07	3.06E+08	6.63E+07
kprop	M-1 s-1	1.00E+03	3.00E+03	3.00E+03	2.33E+03	5.00E+03	4.00E+03	4.00E+03	3.00E+03	3.17E+03	4.27E+02

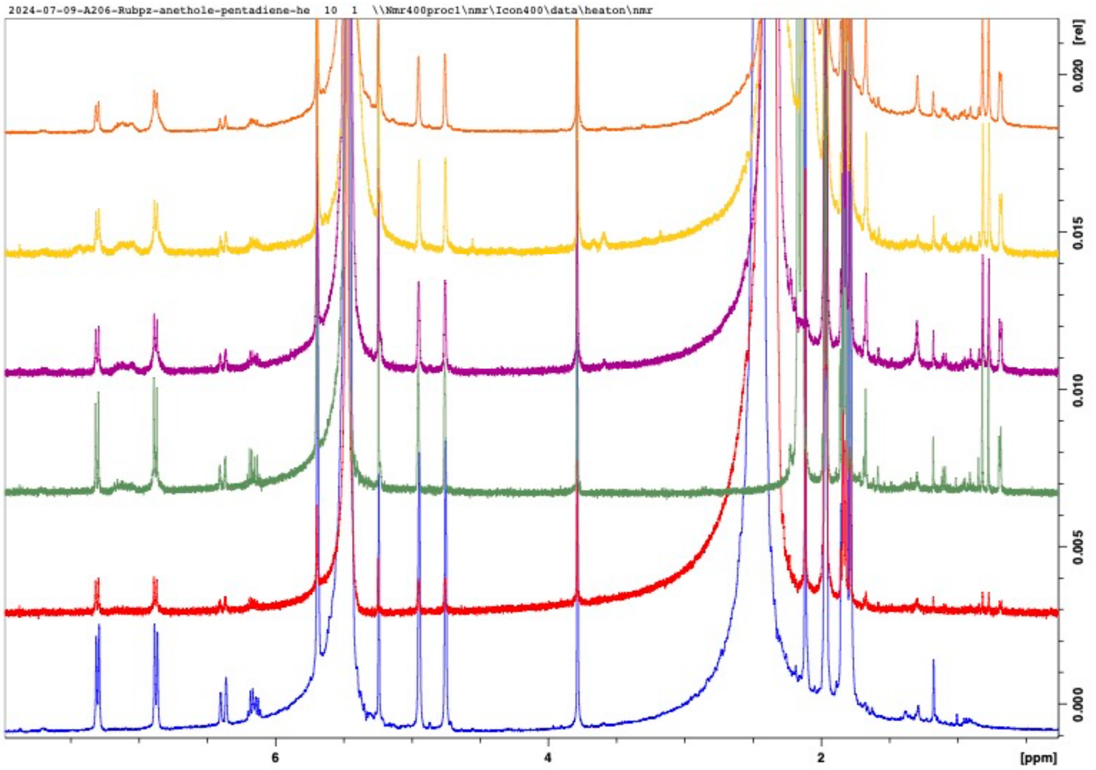

Figure S1. H1 NMR spectra for reaction quantum yield measurements between anethole and 2,4-dimethyl-1,3-pentadiene and catalyzed by $Ru(bpz)_3^{2+}$. Prominent [4+2] product peaks can be observed at 0.7 ppm and 7.1 ppm. From bottom to top samples are shown from T=0 to 2.5 hr.

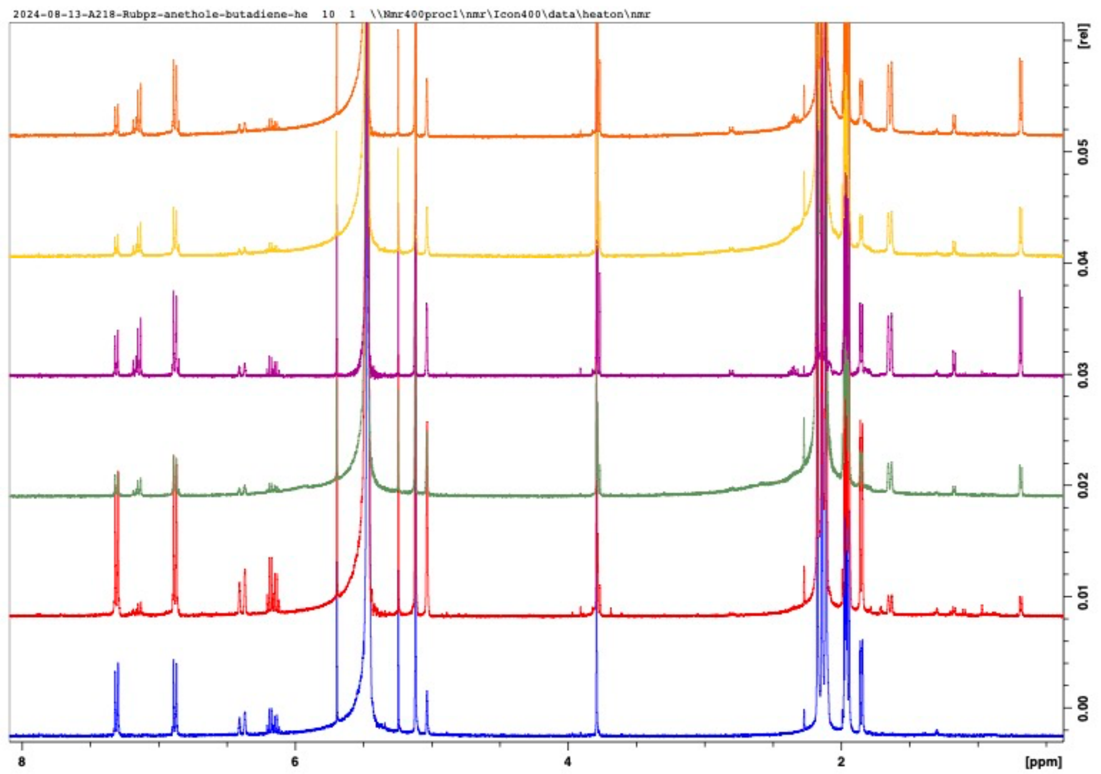

Figure S2. H1 NMR spectra for reaction quantum yield measurements between anethole and 2,3-dimethyl-1,3-butadiene and catalyzed by $Ru(bpz)_3^{2+}$. Prominent [4+2] product peaks can be observed at 0.7 ppm and 7.1 ppm. From bottom to top samples are shown from T=0 to 2.5 hr.

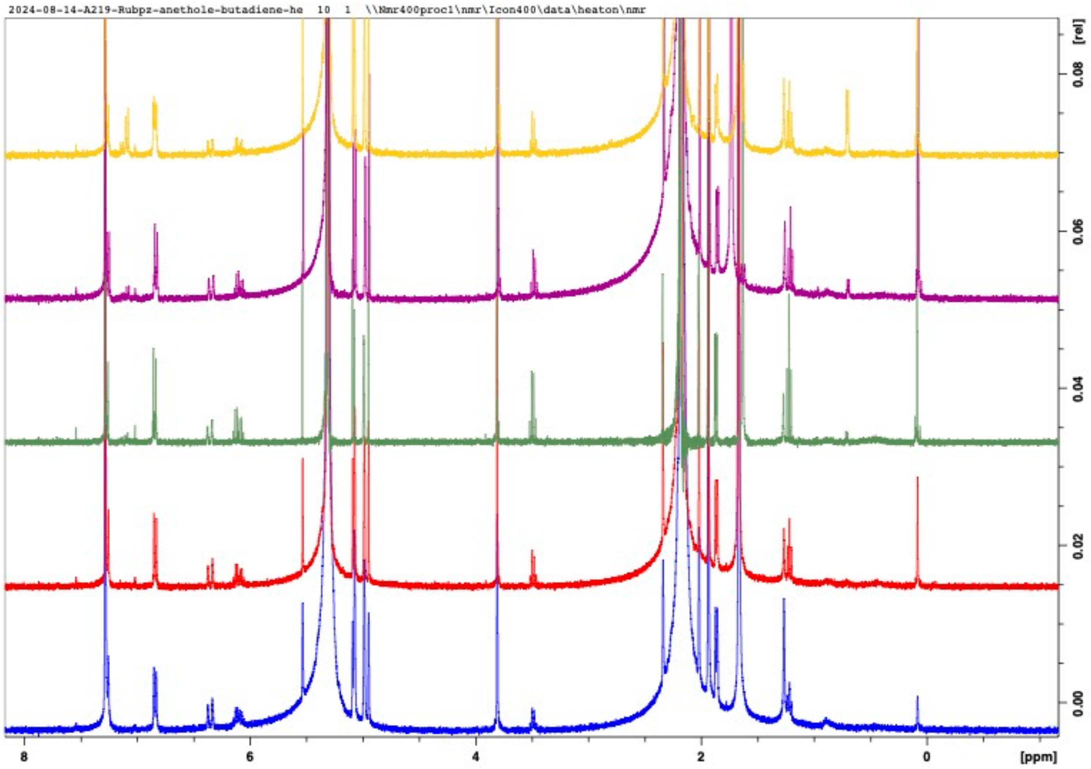

Figure S3. H1 NMR spectra for reaction quantum yield measurements between anethole and 1-acetoxy-1,3-butadiene and catalyzed by $Ru(bpz)_3^{2+}$. Prominent [4+2] product peaks can be observed at 0.7 ppm and 7.1 ppm. From bottom to top samples are shown from T=0 to 2.5 hr.

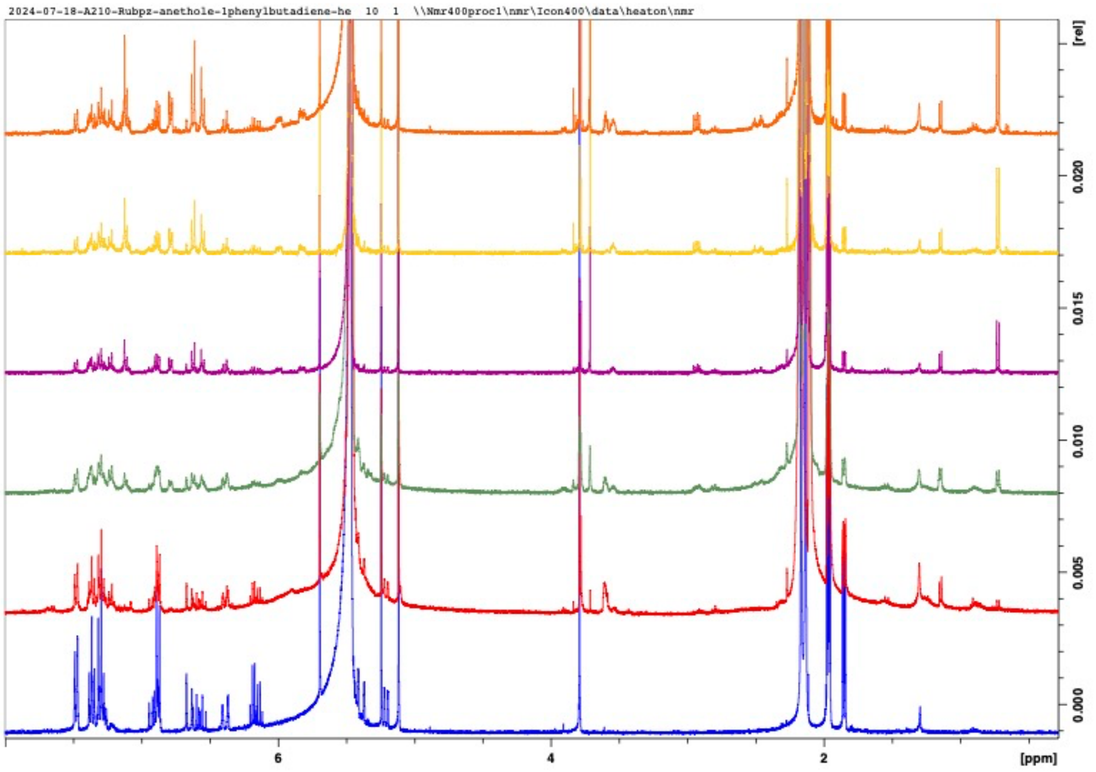

Figure S4. H^1 NMR spectra for reaction quantum yield measurements between anethole and 1-phenyl-1,3-butadiene and catalyzed by $Ru(bpz)_3^{2+}$. Prominent [4+2] product peaks can be observed at 0.7 ppm and 7.1 ppm. From bottom to top samples are shown from T=0 to 2.5 hr.

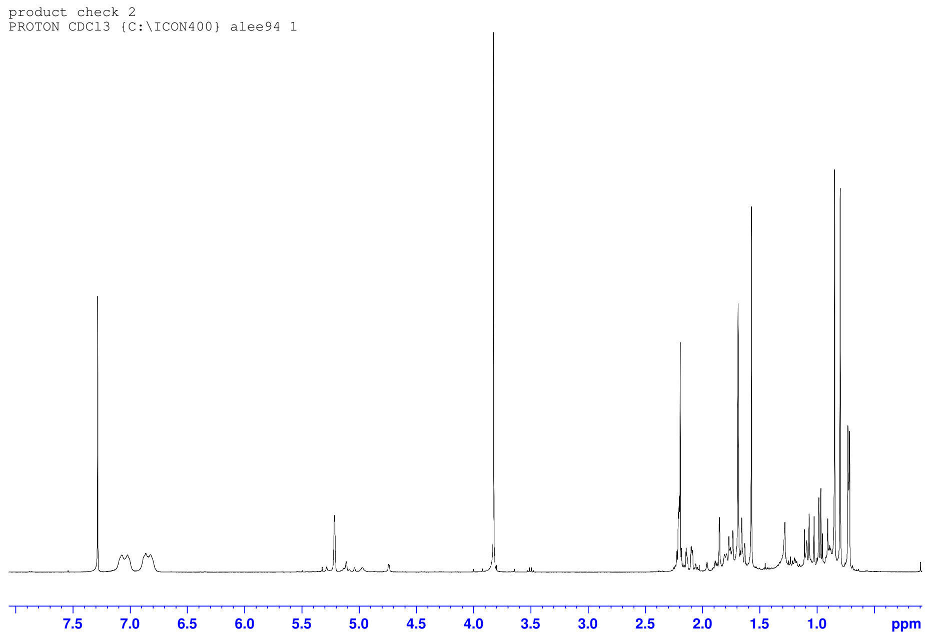

Figure S5. H1 NMR of isolated [4+2] product synthesized with anethole and 2,4-dimethyl-1,3-pentadiene.

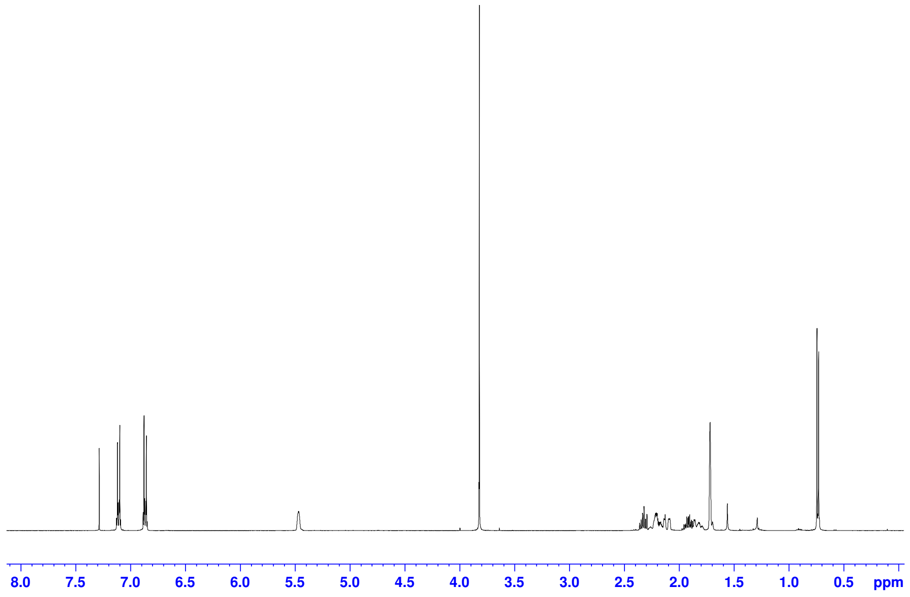

Figure S6. H1 NMR of isolated [4+2] product synthesized with anethole and isoprene

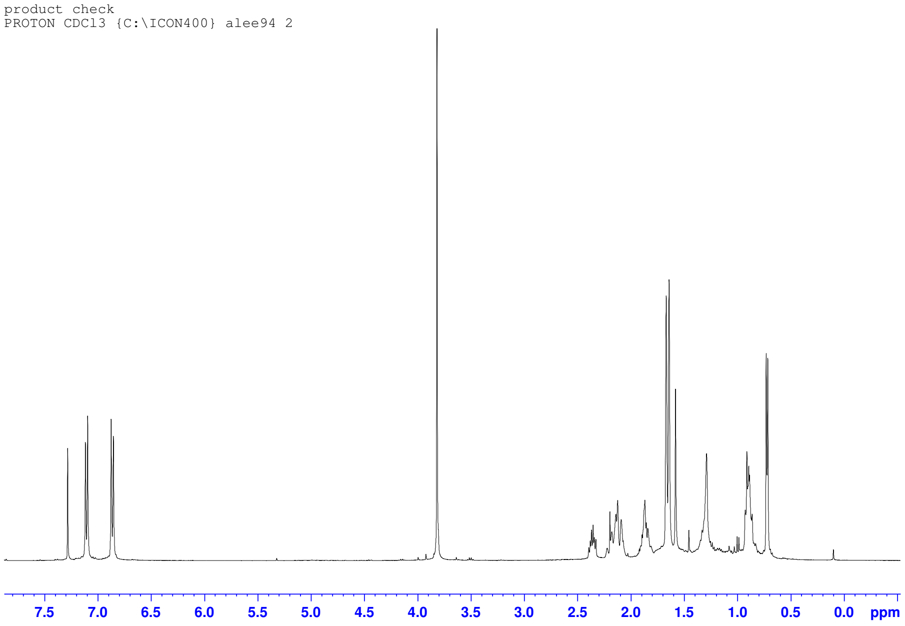

Figure S7. H1 NMR of isolated [4+2] product synthesized with anethole and 2,3-dimethyl-1,3-butadiene.

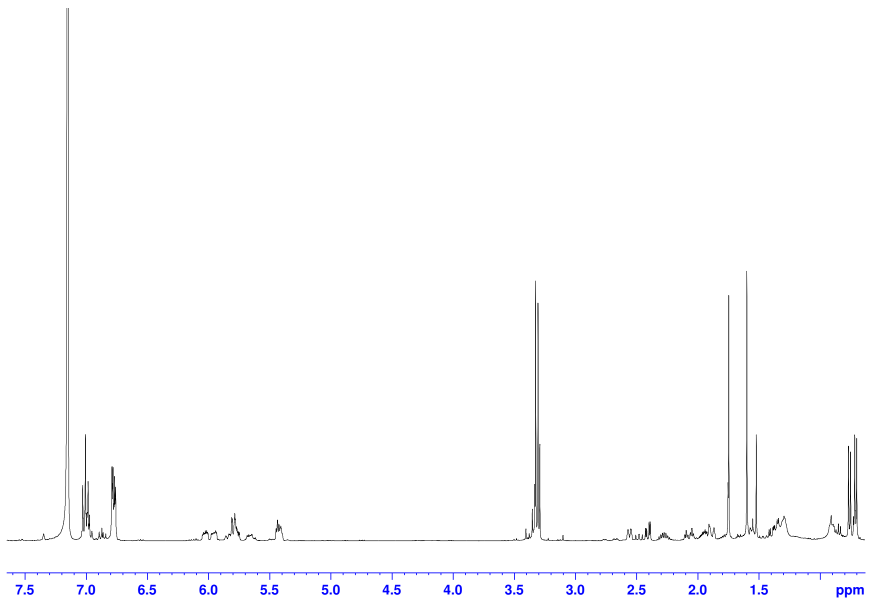

Figure S8. H1 NMR of isolated [4+2] product synthesized with anethole and 1-acetoxy-1,3-butadiene

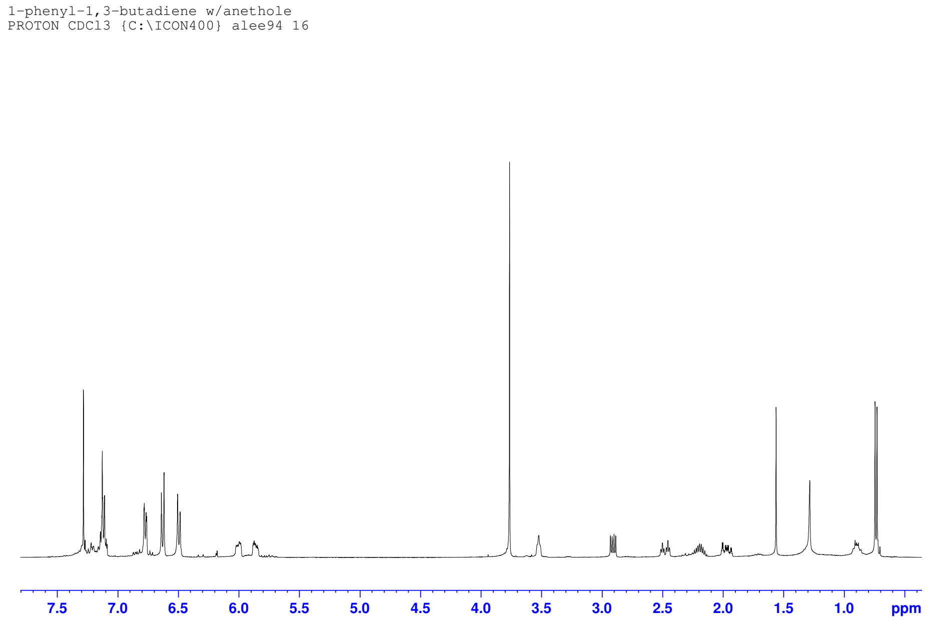

Figure S9. H1 NMR of isolated [4+2] product synthesized with anethole and 1-phenyl-1,3-butadiene

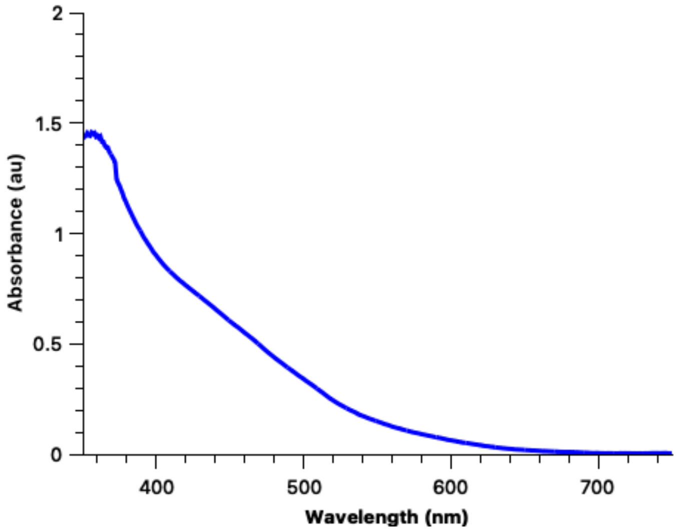

Figure S10. The electrochemically generated difference spectrum for the [4+2] and [4+2] $^{\bullet +}$ product made with anethole and 1-phenyl -1,3-butadiene, in acetonitrile with 0.1 M NH_4PF_6.

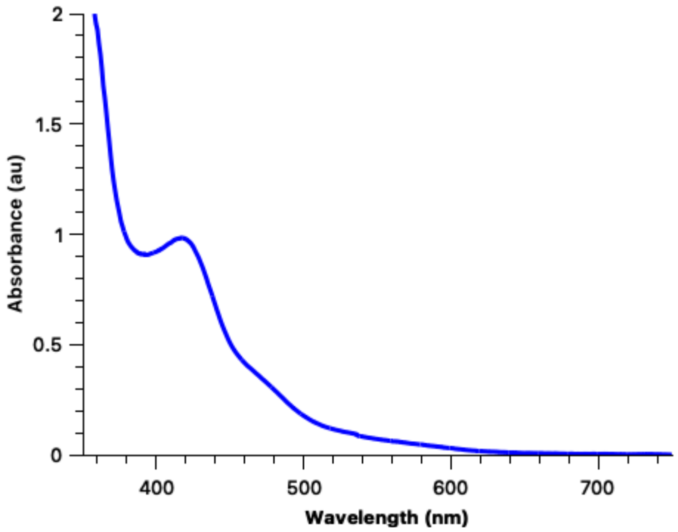

Figure S11. The electrochemically generated difference spectrum for the [4+2] and [4+2] $^{\bullet +}$ product made with anethole and 1-acetoxy-1,3-butadiene, in acetonitrile with 0.1 M NH_4PF_6.

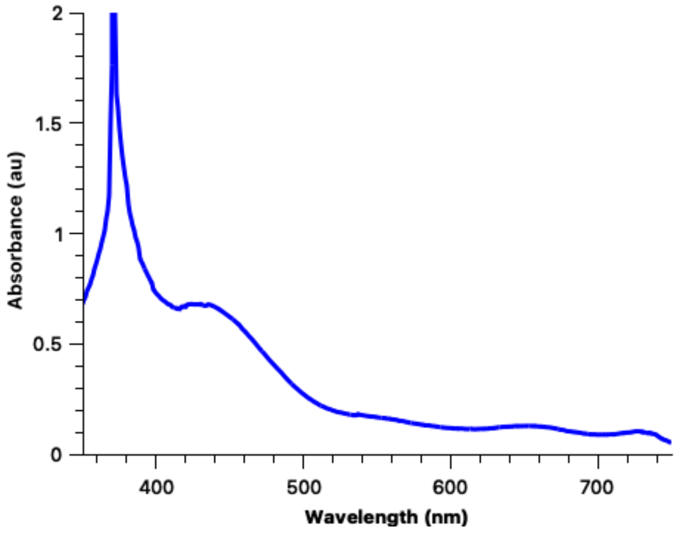

Figure S12. The electrochemically generated difference spectrum for the [4+2] and $[4+2]^{\bullet+}$ product made with anethole and 2,4-dimethyl-1,3-pentadiene, in acetonitrile with 0.1 M NH_4PF_6.

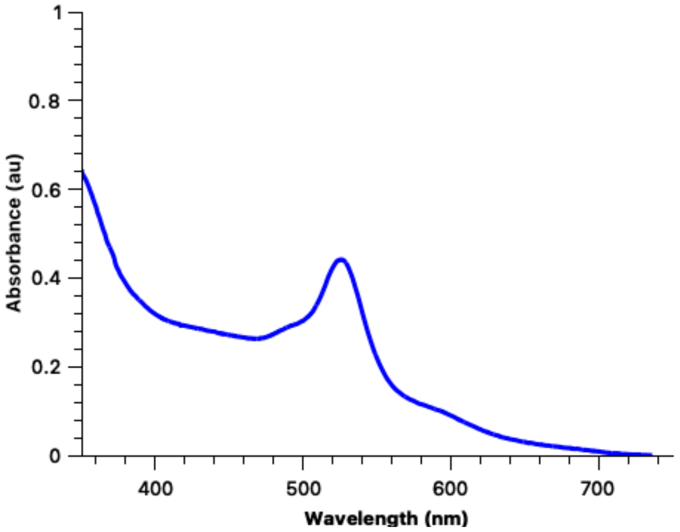

Figure S13. The electrochemically generated difference spectrum for the [4+2] and $[4+2]^{\bullet+}$ product made with anethole and 2,3-dimethyl-1,3-butadiene, in acetonitrile with 0.1 M NH_4PF_6.

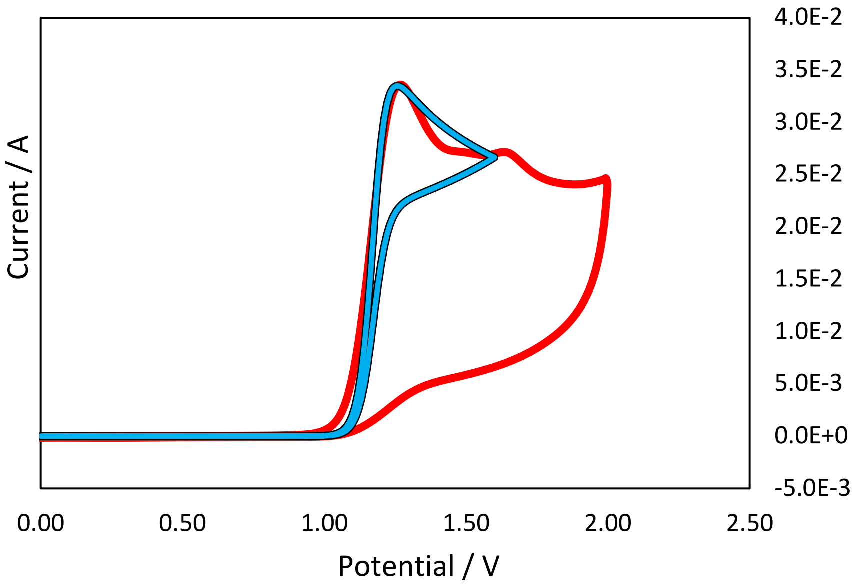

Figure S14. The cyclic voltammogram collected for anethole (red) and the fit achieved through electrochemical simulation (blue) to determine oxidation potential

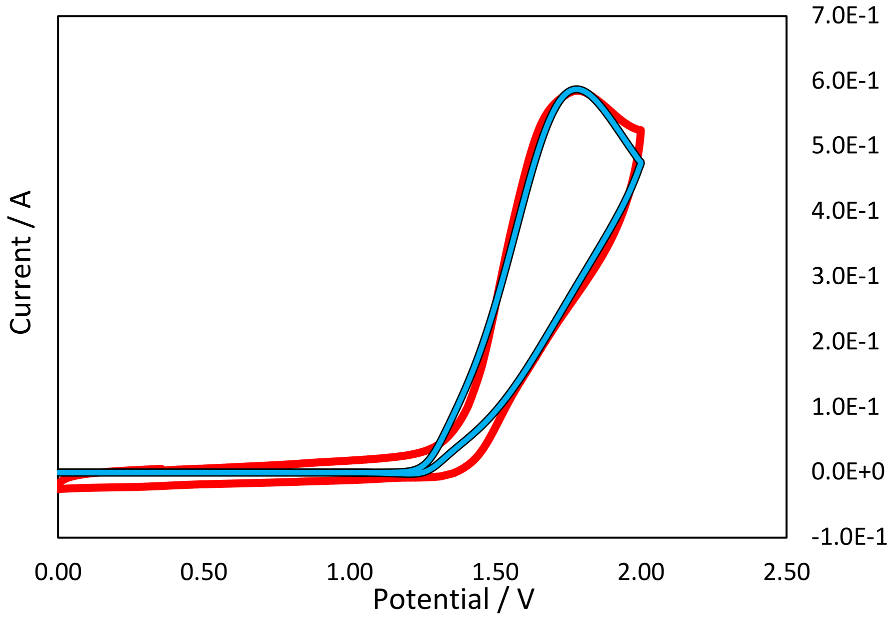

Figure S15. The cyclic voltammogram collected for the [4+2] cycloaddition product of anethole and 2,4-dimethyl-1,3-pentadiene (red) and the fit achieved through electrochemical simulation (blue) to determine oxidation potential

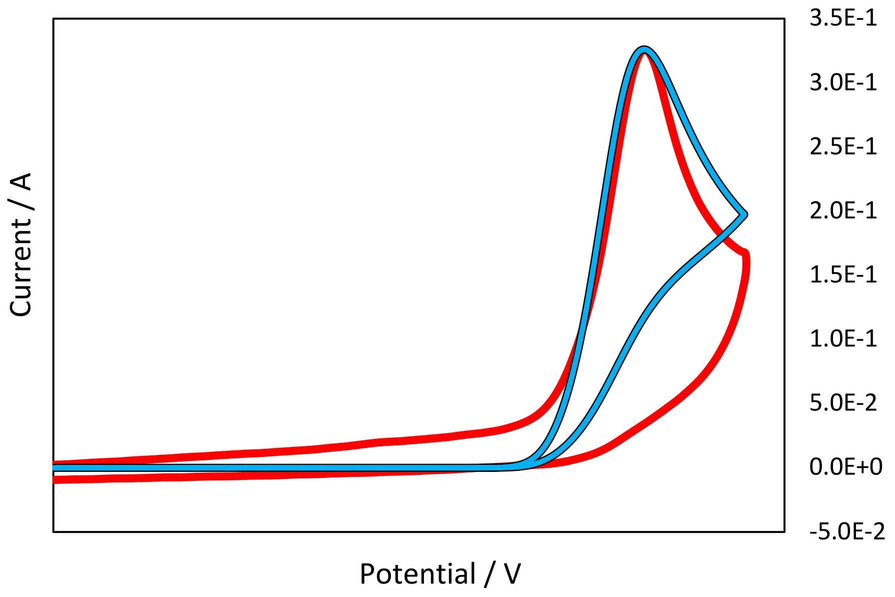

Figure S16. The cyclic voltammogram collected for the [4+2] cycloaddition product of anethole and isoprene (red) and the fit achieved through electrochemical simulation (blue) to determine oxidation potential

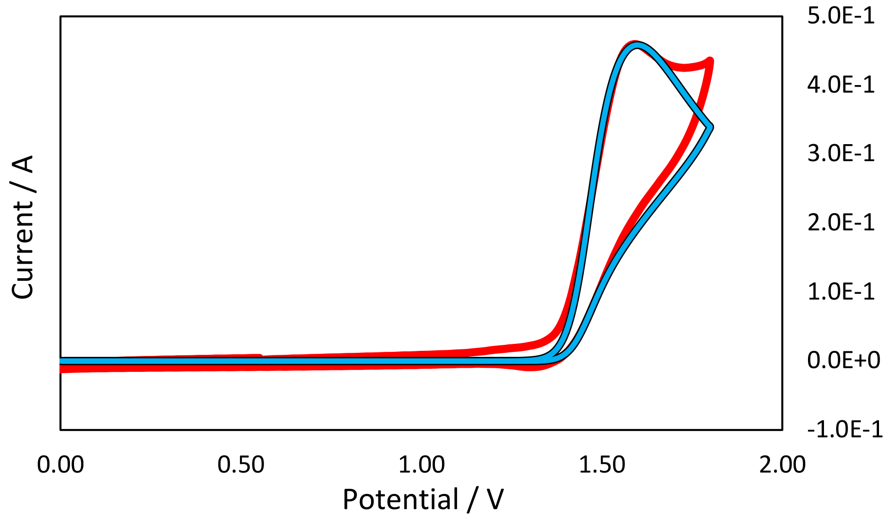

Figure S17. The cyclic voltammogram collected for the [4+2] cycloaddition product of anethole and 2,3-dimethyl-1,3-butadiene (red) and the fit achieved through electrochemical simulation (blue) to determine oxidation potential

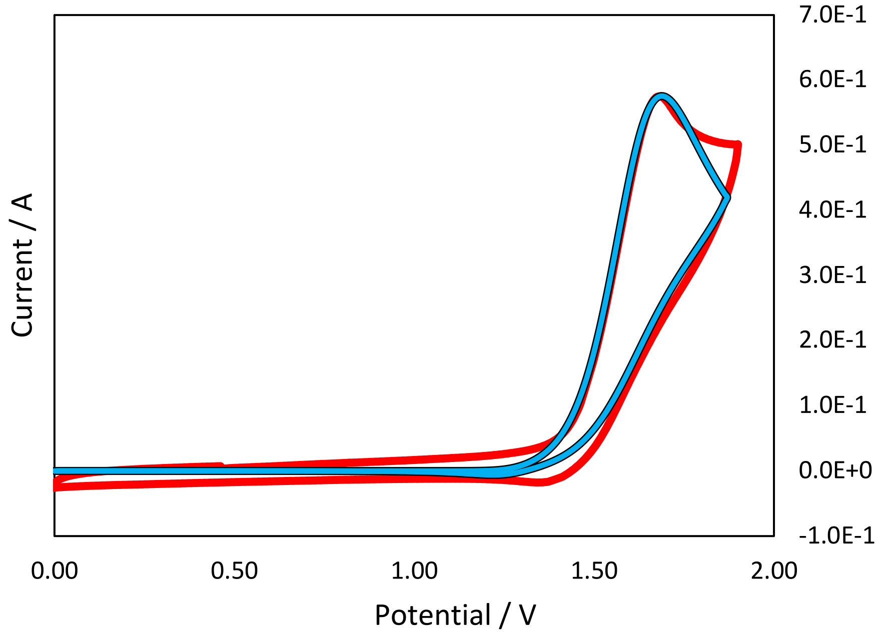

Figure S18. The cyclic voltammogram collected for the [4+2] cycloaddition product of anethole and 1-acetoxy-1,3-butadiene (red) and the fit achieved through electrochemical simulation (blue) to determine oxidation potential

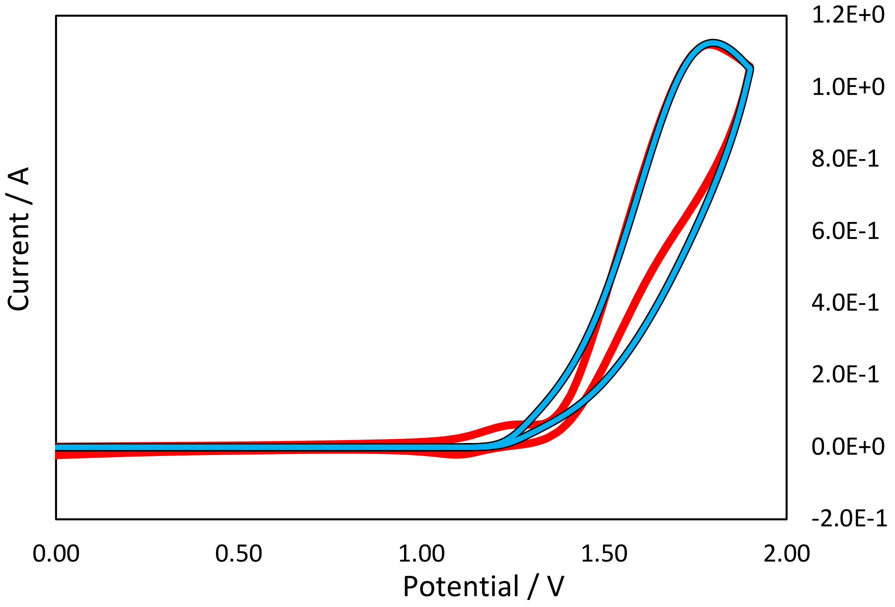

Figure S19. The cyclic voltammogram collected for the [4+2] cycloaddition product of anethole and 1-phenyl-1,3-butadiene (red) and the fit achieved through electrochemical simulation (blue) to determine oxidation potential

Table S9. Oxidation potentials found for each [4+2] cycloaddition product made by anethole and the specified diene and the calculated ΔG value in kJ

Diene	Product E° (V)	ΔG (kJ)
Trans-anethole	1.17	
2,4-dimethyl-1,3-pentadiene	1.43	25.28
Isoprene	1.37	19.78
1-acetoxy-1,3-butadiene	1.35	17.37
2,3-dimethyl-1,3-butadiene	1.33	15.44
1-phenyl-1,3-butadiene	1.32	14.96

Table S10. Constants and values from cyclic volatmmetry simulation to determine the oxidation potentials of *trans*-anethole and each subsequent [4+2] cyclization product

	Units	*trans*-anethole	2,4-dimethyl-1,3-pentadiene	isoprene	2,3-diemthyl-1,3-butadiene	1-acetoxy-1,3-butadiene	1-phenyl-1,3-butadiene
Species Concentration	mol/cm³	1.06E-02	2.00E-02	1.35E-02	2.80E-02	2.50E-02	6.07E-02
**Redox 1 constants**							
$E^{o\prime}_{(1,2)}$	V	1.17	1.43	1.37	1.33	1.35	1.32
$n_{(1,2)}$	e^-	1	1	1	1	1	1
$n_{a\,(1,2)}$	e^-	1	1	1	1	1	1
$k^o_{(1,2)}$	cm/s	0.00068	0.00261	0.000451	0.00045	0.00015	3.00E-04
$a_{(1,2)}$		0.0023	0.275	0.4	0.205	0.37	0.21
$D_{(1)}$	cm²/s	1.00E-05	1.00E-05	1.00E-05	1.3E-05	1.00E-05	1.3E-05
$D_{(2)}$	cm²/s	1.50E-06	1.50E-06	1.50E-06	2.0E-06	1.50E-06	3.0E-06
K_{R1}	s^{-1}		10	100	50	5	100
**Simulation parameters**							
E_1	V	0	0	0	0	0	0
E_2	V	1.6	1.8	1.89	2	1.87	1.9
v	V/s	0.05	0.5	0.5	0.5	0.5	0.5
A	cm²	0.07066	0.07066	0.07066	0.07066	0.07066	0.07066
T	K	293.15	293.15	293.15	293.15	293.15	293.15
x_{Scale}	%	100	100	100	100	100	100
g	unitless	1	1	1	1	1	1
CC	(I or P)	I	I	I	I	I	I

Computational data

The [4+2] products can take a range of different conformations. We first define the left-chair and right chair based on the symetry around the double bond in the cyclohexane ring (Figure S27). These might also be called half chairs because of the double bond. In the 1-phenyl-1,3-butadiene and 1-acetoxyl-1,3-butadiene products; 4''-methoxy-3'-methyl-1',2',3',4'-tetrahydro-1,1':2',1''-terphenyl and 4'-methoxy-6-methyl-1,2,5,6-tetrahydro-[1,1'-biphenyl]-2-yl acetate, respectively; this leads to an additional stereocenter at carbon 13 where the phenyl or acetoxyl substituent can be up or down in the product. All structures can be downloaded directly from https://github.com/fredingroup/photoredox/tree/main/%5B4%2B2%5D

The Gaussian commands were # wB97XD/Def2TZVP opt freq Int=SuperFineGrid

Figure S20. Representative left and right half chairs of cyclohexane and the isoprene product, 4'-methoxy-2,4-dimethyl-1,2,3,6-tetrahydro-1,1'-biphenyl, shown for reference

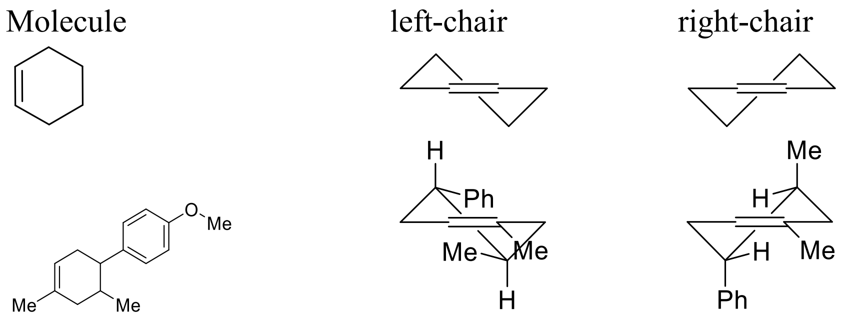

Table S11. ωB97XD/def2TZVP/PCM(DCM) Gibbs Free energies of all compounds, including all isomers of cyclized products.

	Molecule	G (Hartrees)	$G^{\bullet+}$ (Hartrees)
dienes	2,4-dimethyl-1,3-pentadiene	-273.950788	-273.7301065
	isoprene	-195.310353	-195.0737876
	2,3-dimethyl-1,3-butadiene	-234.630477	-234.3989075
	1-acetoxy-1,3-butadiene	-383.896602	-383.6734359
	1-phenyl-1,3-butadiene	-387.048296	-386.8336915
dienophile	*trans*-anethole	-463.50563	-463.295049
left-chair products	4'-methoxy-2,4,6,6-tetramethyl-1,2,3,6-tetrahydro-1,1'-biphenyl	-737.532983	-737.299937
	up 4"-methoxy-3'-methyl-1',2',3',4'-tetrahydro-1,1':2',1"-terphenyl	-850.6343023	-850.398973
	down 4"-methoxy-3'-methyl-1',2',3',4'-tetrahydro-1,1':2',1"-terphenyl	-850.6320973	-850.397673
	up 4'-methoxy-6-methyl-1,2,5,6-tetrahydro-[1,1'-biphenyl]-2-yl acetate	-847.4866782	-847.248285
	down 4'-methoxy-6-methyl-1,2,5,6-tetrahydro-[1,1'-biphenyl]-2-yl acetate	-847.4863107	-847.248285
	4'-methoxy-2,4-dimethyl-1,2,3,6-tetrahydro-1,1'-biphenyl	-658.8978722	-658.665847
	4'-methoxy-2,4,5-trimethyl-1,2,3,6-tetrahydro-1,1'-biphenyl	-698.2165301	-697.984142
right-chair products	4'-methoxy-2,4,6,6-tetramethyl-1,2,3,6-tetrahydro-1,1'-biphenyl	-737.1957774	-737.293681
	up 4"-methoxy-3'-methyl-1',2',3',4'-tetrahydro-1,1':2',1"-terphenyl	-850.6274852	-850.392581
	down 4"-methoxy-3'-methyl-1',2',3',4'-tetrahydro-1,1':2',1"-terphenyl	-850.3034508	-850.394125
	up 4'-methoxy-6-methyl-1,2,5,6-tetrahydro-[1,1'-biphenyl]-2-yl acetate	-847.1889309	-847.236105
	down 4'-methoxy-6-methyl-1,2,5,6-tetrahydro-[1,1'-biphenyl]-2-yl acetate	-847.187494	-847.238339
	4'-methoxy-2,4-dimethyl-1,2,3,6-tetrahydro-1,1'-biphenyl	-658.620488	-658.665234
	4'-methoxy-2,4,5-trimethyl-1,2,3,6-tetrahydro-1,1'-biphenyl	-697.9138767	-697.983857

Table S12. **DLPNO-CCSD(T)/def2-TZVP+def2-TZVP/C single point energies of all compounds.**

	Molecule	E (Hartrees)	$E^{\bullet+}$ (Hartrees)
dienes	2,4-dimethyl-1,3-pentadiene	-273.391759175779	-273.091371511862
	isoprene	-194.915298502678	-194.595279105593
	2,3-dimethyl-1,3-butadiene	-234.153877340229	-233.840772416443
	1-acetoxy-1,3-butadiene	-383.222764250186	-382.921538161436
	1-phenyl-1,3-butadiene	-386.287142292082	-386.00071983733
dienophile	*trans*-anethole	-462.62895677417	-462.355718727743
left-chair products	4'-methoxy-2,4,6,6-tetramethyl-1,2,3,6-tetrahydro-1,1'-biphenyl	-736.089943287939	-735.805971938715
	up 4''-methoxy-3'-methyl-1',2',3',4'-tetrahydro-1,1':2',1''-terphenyl	-848.987031495956	-848.703654548111
	down 4''-methoxy-3'-methyl-1',2',3',4'-tetrahydro-1,1':2',1''-terphenyl	-848.984908154104	-848.702211101217
	up 4'-methoxy-6-methyl-1,2,5,6-tetrahydro-[1,1'-biphenyl]-2-yl acetate	-845.916921237834	-845.6286485397
	down 4'-methoxy-6-methyl-1,2,5,6-tetrahydro-[1,1'-biphenyl]-2-yl acetate	-845.925404594516	-845.636698258533
	4'-methoxy-2,4-dimethyl-1,2,3,6-tetrahydro-1,1'-biphenyl	-657.616927726679	-657.332028295479
	4'-methoxy-2,4,5-trimethyl-1,2,3,6-tetrahydro-1,1'-biphenyl	-696.853682306842	-696.569322742399
right-chair products	4'-methoxy-2,4,6,6-tetramethyl-1,2,3,6-tetrahydro-1,1'-biphenyl	-736.082690378848	-735.800242914571
	up 4''-methoxy-3'-methyl-1',2',3',4'-tetrahydro-1,1':2',1''-terphenyl	-848.979917376334	-848.695396699018
	down 4''-methoxy-3'-methyl-1',2',3',4'-tetrahydro-1,1':2',1''-terphenyl	-848.980571310344	-848.698887744342
	up 4'-methoxy-6-methyl-1,2,5,6-tetrahydro-[1,1'-biphenyl]-2-yl acetate	-845.910132773511	-845.616590101898
	down 4'-methoxy-6-methyl-1,2,5,6-tetrahydro-[1,1'-biphenyl]-2-yl acetate	-845.907943540883	-845.623893505766
	4'-methoxy-2,4-dimethyl-1,2,3,6-tetrahydro-1,1'-biphenyl	-657.615062136689	-657.33207724229
	4'-methoxy-2,4,5-trimethyl-1,2,3,6-tetrahydro-1,1'-biphenyl	-696.852107906373	-696.569621886873

Table S13. **Predicted favorability of the lowest energy ground state (ΔG) and excited state($\Delta G^{\bullet+}$) paths for each diene and the predicted "hole" catalysis potential(ΔG_{up}) calculated from single point energies at the DLPNO-CCSD(T)/def2-TZVP+def2-TZVP/C level of theory.**

**Reagent**	ΔG (eV)	$\Delta G^{\bullet+}$ (eV)	ΔG_{up} (eV)
2,4-dimethyl-1,3-pentadiene	1.88	1.59	0.29
isoprene	1.98	1.66	0.32
2,4-dimethyl-1,3-butadiene	1.93	1.63	0.30
1-acetoxy-1,3-butadiene	2.01	1.58	0.42
1-phenyl-1,3-butadiene	1.93	1.65	0.28

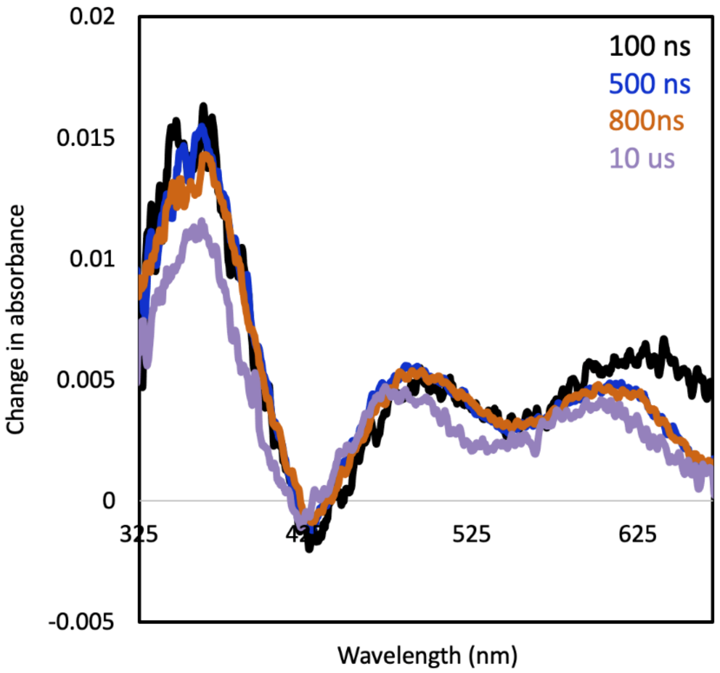

Figure S21. Transient absorption spectrum displaying the changes in absorbance found at delay times ranging from 100 ns to 10 µs after 430 nm excitation of the sample. The samples contained 53 mM anethole, 50 mM 2,4-dimethyl-1,3-pentadiene and 27 µM of the photocatalyst [Ru(bpz)3]2+ and were open to air.

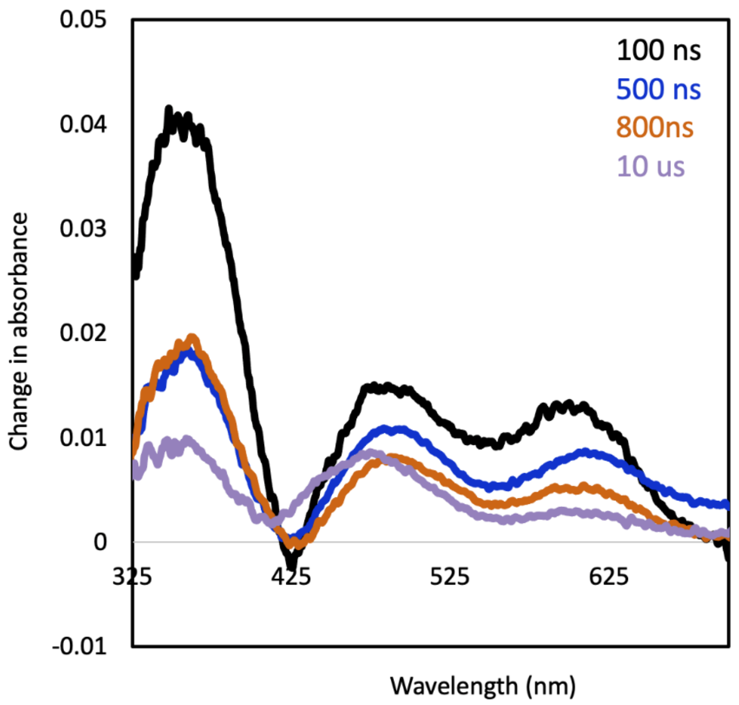

Figure S22. Transient absorption spectrum displaying the changes in absorbance found at delay times ranging from 100 ns to 10 µs after 430 nm excitation of the sample. The samples contained 53 mM anethole, 0.50 M 2,3-dimethyl-1,3-butadiene and 27 µM of the photocatalyst $[Ru(bpz)_3]^{2+}$ and were open to air.

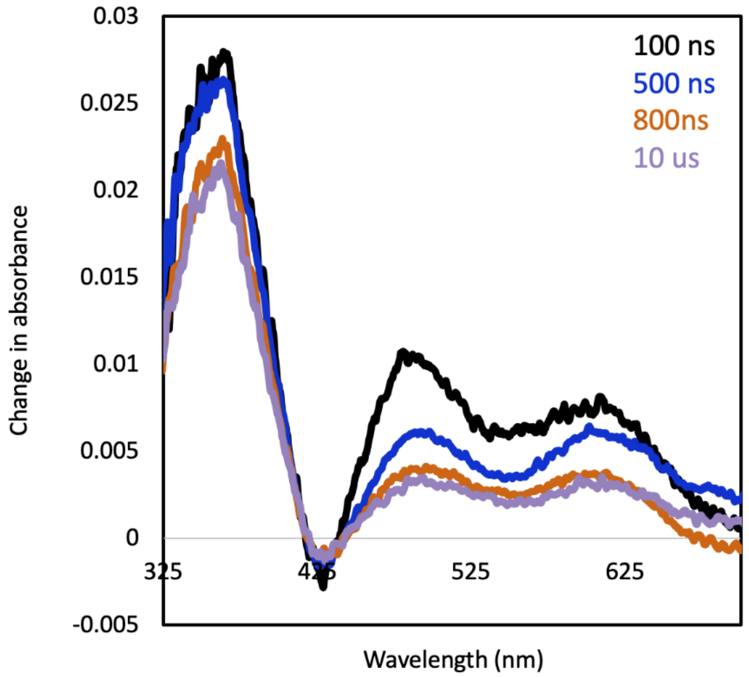

Figure S23. Transient absorption spectrum displaying the changes in absorbance found at delay times ranging from 100 ns to 10 µs after 430 nm excitation of the sample. The samples contained 53 mM anethole, 0.50 M 1-acetoxy-1,3-butadiene and 27 µM of the photocatalyst $[Ru(bpz)_3]^{2+}$ and were open to air.

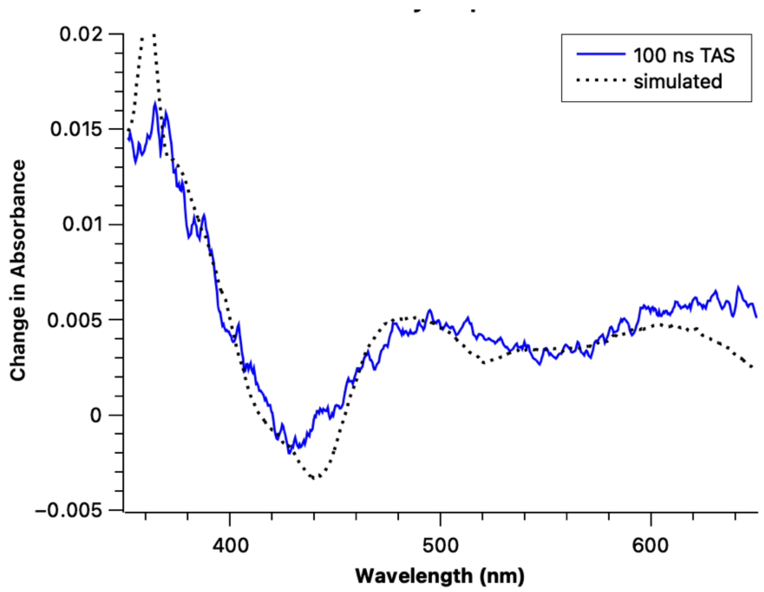

Figure S24. The 100 ns TAS spectrum for [Ru(bpz)3]$^{2+}$ and anethole excited at 430 nm with the difference spectra obtained through spectro-electrochemical analysis with anethole and 2,4-dimethyl-1,3-pentadiene.

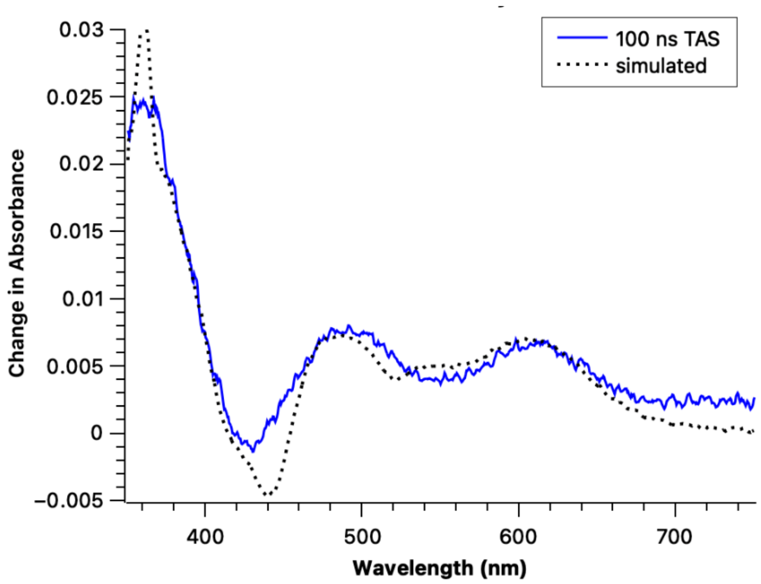

Figure S25. The 100 ns TAS spectrum for [Ru(bpz)3]$^{2+}$ and anethole excited at 430 nm with the difference spectra obtained through spectro-electrochemical analysis with anethole and 2,3-dimethyl-1,3-butadiene.

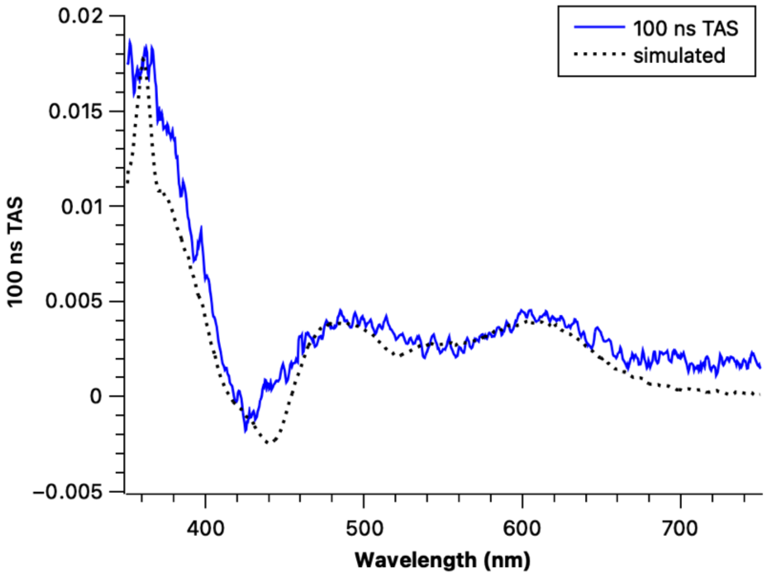

Figure S26. The 100 ns TAS spectrum for [Ru(bpz)3] $^{2+}$ and anethole excited at 430 nm with the difference spectra obtained through spectro-electrochemical analysis with anethole and 1-acetoxy-1,3-butadiene.

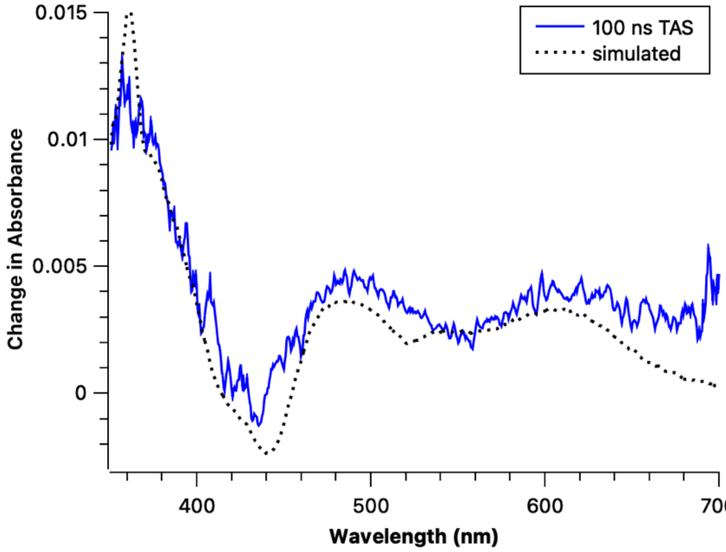

Figure S27. The 100 ns TAS spectrum for [Ru(bpz)3]$^{2+}$ and anethole excited at 430 nm with the difference spectra obtained through spectro-electrochemical analysis with anethole and 1-phenyl-1,3-butadiene.

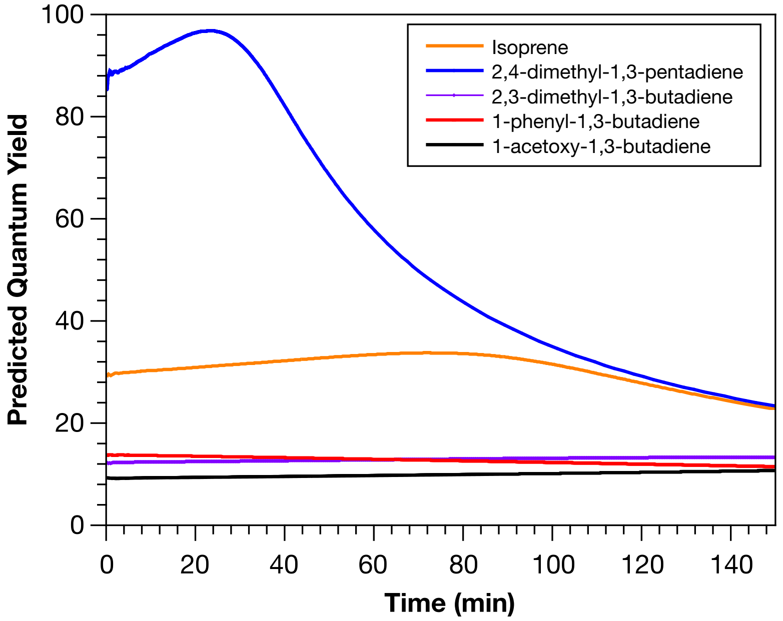

Figure S28. Predicted QY from kinetic modelling that utilizes rate constants determined in the transient absorption experiments.

Computational Optimized Structures

1-acetoxy-1,3-butadiene

C	-2.405968	-0.606817	-0.171270
C	-3.449027	0.108447	-0.569580
C	-3.722588	1.466006	-0.094976
C	-3.475975	1.923827	1.128679
H	-1.640429	-0.257427	0.507573
H	-4.123093	-0.316269	-1.305811
H	-4.177553	2.132605	-0.821697
H	-3.072218	1.285978	1.906869
H	-3.682224	2.953544	1.390822
O	-2.239494	-1.886765	-0.639500
C	-1.120420	-2.567636	-0.291318
O	-0.258706	-2.106141	0.404448
C	-1.132347	-3.937758	-0.890297
H	-1.200640	-3.862808	-1.975839
H	-0.228065	-4.468827	-0.609088
H	-2.010060	-4.482371	-0.541242

1-acetoxy-1,3-butadiene_oxidized

C	-2.475646	-0.678918	-0.100285
C	-3.505628	0.111706	-0.590524
C	-3.856247	1.362482	-0.066116
C	-3.252660	1.991751	0.991222
H	-1.839215	-0.408738	0.736917
H	-4.064687	-0.276902	-1.432594
H	-4.682548	1.867870	-0.550618
H	-2.421455	1.568533	1.538771
H	-3.606954	2.961001	1.313580
O	-2.244449	-1.817269	-0.667167
C	-1.159321	-2.638704	-0.143380
O	-0.527906	-2.253520	0.771937
C	-1.080775	-3.879846	-0.941172
H	-0.910318	-3.623481	-1.987498
H	-0.276424	-4.502898	-0.564049
H	-2.034576	-4.405480	-0.881253

1-phenyl-1,3-butadiene

C	-2.141714	0.194976	-0.316971
C	-3.390488	-0.271266	-0.229735
C	-3.817970	-1.647457	-0.496845
C	-3.054613	-2.672405	-0.869910
H	-1.348777	-0.487034	-0.611113
H	-4.187735	0.403380	0.063299
H	-4.881000	-1.826689	-0.369475
H	-1.984969	-2.581358	-1.019869
H	-3.487078	-3.649879	-1.038893
C	-1.693116	1.566768	-0.054335
C	-0.336943	1.866208	-0.199588
C	-2.554761	2.596765	0.335821
C	0.146129	3.144437	0.034659
H	0.347705	1.081860	-0.501282
C	-2.074186	3.872597	0.569885
H	-3.612829	2.404786	0.459311
C	-0.721374	4.154081	0.420789
H	1.202315	3.350458	-0.085100
H	-2.759280	4.655137	0.871210
H	-0.349289	5.153830	0.604926

1-phenyl-1,3-butadiene_oxidized

C	-2.122649	0.214154	-0.315351
C	-3.423834	-0.256327	-0.220686
C	-3.829007	-1.586082	-0.480054
C	-3.010733	-2.601343	-0.859670
H	-1.344435	-0.480261	-0.610173
H	-4.206385	0.431151	0.073134
H	-4.884579	-1.796978	-0.361547
H	-1.945058	-2.479176	-1.001462
H	-3.415332	-3.588637	-1.035527
C	-1.700266	1.535655	-0.060776
C	-0.322335	1.831957	-0.210274
C	-2.582535	2.573239	0.334524
C	0.149124	3.100149	0.023387
H	0.355311	1.043421	-0.511842
C	-2.100621	3.835960	0.565168
H	-3.638326	2.378848	0.457040
C	-0.738301	4.102558	0.410931
H	1.200800	3.321854	-0.091799
H	-2.773471	4.626622	0.866484
H	-0.367339	5.102432	0.595277

2,3-dimethyl-1,3-butadiene

C	-1.772120	-1.105601	-0.837504
C	-2.966331	-1.129118	-0.250467
C	-3.655259	0.125957	0.151499
C	-3.004473	1.101754	0.780470
H	-1.265390	-2.022418	-1.115591
H	-1.273054	-0.171932	-1.065635
H	-1.958442	1.004962	1.043718
H	-3.505284	2.021777	1.058691
C	-3.692044	-2.419346	0.016591
H	-4.609439	-2.482577	-0.573538
H	-3.068611	-3.277061	-0.232859
H	-3.983414	-2.492621	1.067417
C	-5.122130	0.228606	-0.165392
H	-5.305570	0.044706	-1.227010
H	-5.696453	-0.516129	0.390833
H	-5.508763	1.214580	0.089439

2,3-dimethyl-1,3-butadiene_oxidized

C	-1.635677	-1.156921	-0.443662
C	-2.995544	-1.123125	-0.220715
C	-3.665138	0.098790	0.120858
C	-2.959141	1.251529	0.390759
H	-1.159321	-2.086795	-0.724679
H	-0.996918	-0.289766	-0.371064
H	-1.881998	1.313766	0.357160
H	-3.487562	2.153203	0.670593
C	-3.792947	-2.374047	-0.363570
H	-4.545446	-2.258635	-1.147428
H	-3.162478	-3.224969	-0.604910
H	-4.335155	-2.585625	0.561171
C	-5.152626	0.112136	0.211782
H	-5.593177	-0.208582	-0.735295
H	-5.494202	-0.595864	0.970774
H	-5.529448	1.100443	0.458887

2,4-dimethyl-1,3-pentadiene

C	-1.743735	-0.592074	1.346136
C	-2.557226	0.467252	1.385082
C	-3.218665	1.158568	0.263979
C	-3.850803	0.521349	-0.720558
H	-2.734678	0.921314	2.358342
H	-4.331427	1.067841	-1.523835
H	-3.921632	-0.558555	-0.745532
C	-1.314960	-1.299300	0.093981
H	-1.843421	-2.251630	-0.009837
H	-1.506223	-0.711430	-0.801967
H	-0.248980	-1.534670	0.144925
C	-1.166028	-1.158600	2.611372
H	-0.073542	-1.107982	2.591980
H	-1.522509	-0.631638	3.496247
H	-1.428362	-2.216168	2.709747
C	-3.172410	2.661005	0.329972
H	-3.608647	3.015855	1.267766
H	-2.138807	3.015825	0.307993
H	-3.715349	3.117182	-0.497494

2,4-dimethyl-1,3-pentadiene_oxidized

C	-1.552306	0.953778	0.107650
C	-1.462115	1.709089	1.375380
H	-0.941784	2.652963	1.173520
H	-2.464774	1.989569	1.707903
H	-0.937711	1.173912	2.162304
C	-0.774399	-0.194932	-0.031863
H	-0.049448	-0.350947	0.760878
C	-0.753440	-1.166407	-1.052213
C	0.399507	-2.125229	-1.051046
H	0.350684	-2.759330	-0.162912
H	0.389744	-2.763828	-1.930953
H	1.344865	-1.583131	-1.011756
C	-1.773114	-1.326316	-1.968245
H	-2.676754	-0.738453	-1.950850
H	-1.708255	-2.105184	-2.716417
C	-2.437956	1.527946	-0.930486
H	-3.431786	1.075013	-0.826740
H	-2.566978	2.596294	-0.765604
H	-2.083174	1.352927	-1.942947

isoprene

C	-1.979302	-1.480704	-0.898026
C	-3.139439	-1.487323	-0.252143
C	-3.898817	-0.290986	0.160612
C	-3.297008	0.811409	0.602249
H	-1.479010	-2.404100	-1.161297
H	-1.498426	-0.554551	-1.192304
H	-3.606832	-2.442356	-0.023303
H	-2.219835	0.866404	0.702917
H	-3.867960	1.687002	0.887319
C	-5.394099	-0.410945	0.070034
H	-5.704050	-0.622082	-0.956596
H	-5.747951	-1.242525	0.685404
H	-5.889886	0.500229	0.402782

isoprene_oxidized

C	-1.894667	-1.484940	-0.705629
C	-3.240172	-1.436258	-0.494463
C	-3.944818	-0.326467	0.043299
C	-3.284318	0.836608	0.422573
H	-1.445347	-2.377368	-1.120072
H	-1.228389	-0.663534	-0.481033
H	-3.829794	-2.307473	-0.755823
H	-2.215226	0.960643	0.327708
H	-3.843619	1.666106	0.834102
C	-5.417392	-0.441940	0.207038
H	-5.872721	-0.784116	-0.724182
H	-5.632696	-1.206663	0.959391
H	-5.873457	0.494874	0.514740

trans-anethole

C	-0.865071	-3.050747	0.245722
C	-0.080764	-2.230109	0.939698
H	-0.649291	-3.248520	-0.800685
H	-0.327545	-2.051913	1.984279
C	-2.062401	-3.748581	0.803841
H	-2.965586	-3.476375	0.250979
H	-1.957112	-4.833421	0.717386
H	-2.215866	-3.501402	1.855135
C	1.108877	-1.511569	0.460313
C	1.786898	-0.663999	1.329451
C	1.612330	-1.629197	-0.841314
C	2.916412	0.046886	0.941820
H	1.425592	-0.549917	2.345269
C	2.730252	-0.932935	-1.244005
H	1.124109	-2.278602	-1.556973

C	3.395011	-0.085529	-0.354959
H	3.405601	0.692562	1.656783
H	3.111100	-1.031732	-2.252591
O	4.482457	0.554981	-0.842860
C	5.188826	1.423046	0.019820
H	5.588205	0.886153	0.884749
H	6.012323	1.826528	-0.563949
H	4.554246	2.244099	0.364773

trans-anethole_oxidized

C	-0.834714	-3.045241	0.239570
C	-0.052359	-2.190003	0.957914
H	-0.597934	-3.250884	-0.798454
H	-0.321846	-2.010052	1.993469
C	-2.018774	-3.735097	0.786275
H	-2.900216	-3.479948	0.190532
H	-1.892587	-4.817178	0.685456
H	-2.202552	-3.487359	1.829894
C	1.090157	-1.506702	0.477958
C	1.789765	-0.648179	1.374293
C	1.585729	-1.626344	-0.855203
C	2.896387	0.044145	0.991871
H	1.422946	-0.549776	2.387767
C	2.685948	-0.943513	-1.250225
H	1.084022	-2.267065	-1.565667
C	3.365264	-0.093495	-0.335723
H	3.405494	0.687110	1.693395
H	3.073993	-1.023132	-2.256066
O	4.413330	0.520252	-0.811284
C	5.182811	1.405193	0.014490
H	5.598927	0.861577	0.861785
H	5.980468	1.770145	-0.623083
H	4.564343	2.235254	0.353720

4'-methoxy-2,4,5-trimethyl-1,2,3,6-tetrahydro-1,1'-biphenyl

C	-2.027193	-1.545579	-0.131199
C	-3.534260	-1.592203	-0.166317
C	-4.271148	-0.480745	-0.118890
C	-3.639721	0.882840	0.008826
H	-1.633027	-2.260469	-0.861289
H	-1.685938	-1.912060	0.845006
H	-3.747080	1.411750	-0.948386
H	-4.218248	1.471646	0.729126
C	-2.171445	0.889288	0.420047
C	-1.421716	-0.168012	-0.401217
H	-2.113637	0.595339	1.475076
H	-1.591587	0.072418	-1.457562
C	-1.579339	2.284929	0.272939
H	-2.181466	3.018887	0.812920
H	-0.560126	2.332945	0.657957
H	-1.554216	2.580988	-0.779905
C	0.071124	-0.167697	-0.172573
C	0.956871	0.067596	-1.211210
C	0.613461	-0.397473	1.093870
C	2.335823	0.077294	-1.020357
H	0.569656	0.253430	-2.206867
C	1.976330	-0.389763	1.307429
H	-0.043947	-0.580480	1.936294
C	2.852035	-0.152306	0.247701
H	2.984640	0.265341	-1.863829
H	2.385980	-0.566136	2.294035
O	4.173538	-0.164799	0.550584
C	5.097166	0.073256	-0.490658
H	4.955095	1.064370	-0.930946
H	6.084676	0.019963	-0.038936
H	5.018075	-0.684914	-1.275015
C	-4.079081	-2.990258	-0.249713
H	-3.833146	-3.442026	-1.215653
H	-3.610570	-3.617301	0.514716
H	-5.156129	-3.050061	-0.112138
C	-5.771314	-0.433714	-0.192322
H	-6.228333	-1.386102	-0.450315
H	-6.192815	-0.100723	0.761151
H	-6.083019	0.299120	-0.942558

4'-methoxy-2,4,5-trimethyl-1,2,3,6-tetrahydro-1,1'-biphenyl_oxidized

C	-1.989794	-1.530926	-0.126736
C	-3.496515	-1.588568	-0.175451
C	-4.247286	-0.487883	-0.119130
C	-3.638476	0.883005	0.033408
H	-1.581328	-2.234110	-0.858326
H	-1.648629	-1.886700	0.852075
H	-3.752487	1.429453	-0.911955
H	-4.222645	1.447423	0.767167
C	-2.175022	0.906091	0.453686
C	-1.421079	-0.128801	-0.402591
H	-2.117339	0.586662	1.499562
H	-1.605536	0.113414	-1.453027
C	-1.591231	2.307653	0.329325
H	-2.201244	3.024920	0.880393
H	-0.574921	2.363630	0.724539
H	-1.565381	2.622684	-0.717034
C	0.050199	-0.125074	-0.176997
C	0.943209	0.045630	-1.265706
C	0.588415	-0.307586	1.123395
C	2.292151	0.041625	-1.092946
H	0.531414	0.183574	-2.256539
C	1.928355	-0.313310	1.326644
H	-0.080268	-0.438086	1.963224
C	2.812802	-0.139347	0.218055
H	2.955054	0.174697	-1.934625

H	2.362961	-0.445411	2.307724
O	4.075658	-0.161299	0.507077
C	5.085746	-0.001733	-0.506421
H	4.983921	0.973550	-0.979061
H	6.028993	-0.067980	0.023326
H	5.003796	-0.803773	-1.237608
C	-4.016783	-2.993869	-0.285008
H	-3.769718	-3.421625	-1.261165
H	-3.536896	-3.628348	0.465612
H	-5.091790	-3.069469	-0.143253
C	-5.746339	-0.461856	-0.205025
H	-6.184688	-1.413809	-0.493512
H	-6.179402	-0.162561	0.753932
H	-6.061904	0.286693	-0.937204

4'-methoxy-2,4,6,6-tetramethyl-1,2,3,6-tetrahydro-1,1'-biphenyl

C	1.980123	-1.605312	0.117460
C	3.485343	-1.551660	0.221107
C	4.235184	-0.458716	0.148615
C	3.626660	0.897339	-0.059314
H	3.972236	-2.514836	0.362138
H	3.703264	1.463556	0.878904
H	4.226995	1.455583	-0.785306
C	2.169427	0.872820	-0.514679
C	1.390983	-0.174222	0.300587
H	2.153320	0.579823	-1.569775
H	1.563690	0.079237	1.354033
C	1.574439	2.272593	-0.397056
H	2.214316	3.001566	-0.899159
H	0.580013	2.336046	-0.837655
H	1.494365	2.565170	0.653961
C	-0.106333	-0.117128	0.088133
C	-0.970970	-0.127385	1.171629
C	-0.682105	-0.050937	-1.182833
C	-2.353785	-0.088242	1.021704
H	-0.560971	-0.168228	2.174546
C	-2.049977	-0.008828	-1.357374
H	-0.048204	-0.023725	-2.060455
C	-2.900945	-0.030786	-0.252864
H	-2.981189	-0.099122	1.901472
H	-2.481687	0.045094	-2.348830
O	-4.229320	0.013018	-0.519635
C	-5.127387	-0.001301	0.570023
H	-4.978911	0.865404	1.220455
H	-6.125794	0.038863	0.141493
H	-5.024491	-0.916785	1.159571
C	1.456149	-2.518840	1.233028
H	1.718780	-2.125177	2.217663
H	0.370270	-2.616540	1.181734
H	1.888699	-3.517799	1.141311
C	1.616602	-2.242168	-1.232321
H	0.546255	-2.440828	-1.304484
H	1.909161	-1.607701	-2.070610
H	2.141966	-3.193625	-1.340738
C	5.728009	-0.494475	0.285380
H	6.092984	-1.503883	0.476549
H	6.209417	-0.118074	-0.621867
H	6.055362	0.151416	1.105714

4'-methoxy-2,4,6,6-tetramethyl-1,2,3,6-tetrahydro-1,1'-biphenyl_oxidized

C	1.945103	-1.594656	0.121130
C	3.449323	-1.550182	0.229226
C	4.214086	-0.468917	0.147386
C	3.629136	0.895209	-0.073593
H	3.917616	-2.519515	0.383270
H	3.715298	1.470618	0.857057
H	4.234712	1.434189	-0.808284
C	2.175137	0.891789	-0.533971
C	1.391915	-0.134186	0.307625
H	2.155067	0.581213	-1.582402
H	1.575030	0.112767	1.358389
C	1.591840	2.297771	-0.427027
H	2.232612	3.011988	-0.946217
H	0.595050	2.371477	-0.864314
H	1.525469	2.606666	0.619448
C	-0.082448	-0.079197	0.098121
C	-0.953200	-0.122144	1.219470
C	-0.655983	0.000419	-1.197857
C	-2.305590	-0.105347	1.083268
H	-0.517997	-0.169384	2.208767
C	-2.001051	0.017371	-1.365435
H	-0.011523	0.050605	-2.063647
C	-2.858543	-0.041125	-0.225014
H	-2.946078	-0.137703	1.951803
H	-2.458754	0.075087	-2.343134
O	-4.128220	-0.026994	-0.482474
C	-5.109383	-0.081100	0.569848
H	-5.005144	0.789997	1.214188
H	-6.067533	-0.064863	0.063546
H	-4.993425	-1.004579	1.134432
C	1.402466	-2.492456	1.240363
H	1.647371	-2.089638	2.224986
H	0.318601	-2.609039	1.172275
H	1.843292	-3.487836	1.164477
C	1.570265	-2.218567	-1.230338
H	0.499371	-2.418389	-1.304657
H	1.871284	-1.589277	-2.068597
H	2.088137	-3.172953	-1.337800
C	5.704856	-0.526276	0.288119
H	6.053500	-1.538498	0.491742

H	6.191482	-0.167839	-0.623074
H	6.038797	0.124701	1.101156

4'-methoxy-2,4-dimethyl-1,2,3,6-tetrahydro-1,1'-biphenyl

C	-2.003540	-1.569939	-0.133496
C	-3.502497	-1.575804	-0.168885
C	-4.251306	-0.478955	-0.118823
C	-3.632136	0.886782	0.000788
H	-1.613410	-2.283064	-0.865432
H	-1.658728	-1.932623	0.842034
H	-3.986806	-2.545589	-0.242650
H	-3.746520	1.410738	-0.957817
H	-4.203446	1.477846	0.724365
C	-2.157743	0.882707	0.402840
C	-1.404850	-0.185752	-0.407855
H	-2.099160	0.599680	1.460736
H	-1.573368	0.045162	-1.466526
C	-1.559532	2.274381	0.241200
H	-2.159648	3.016055	0.772767
H	-0.540533	2.323219	0.626327
H	-1.532744	2.559009	-0.814747
C	0.087229	-0.175156	-0.175060
C	0.974451	0.065028	-1.211329
C	0.627138	-0.399317	1.093409
C	2.352652	0.084935	-1.016081
H	0.589107	0.246822	-2.208435
C	1.989233	-0.381381	1.311262
H	-0.031593	-0.585786	1.934043
C	2.866503	-0.139087	0.253951
H	3.002823	0.276479	-1.857718
H	2.397066	-0.553433	2.299363
O	4.187015	-0.141530	0.561050
C	5.112249	0.102375	-0.477433
H	4.963447	1.091503	-0.919892
H	6.098589	0.057971	-0.022222
H	5.042097	-0.657729	-1.260767
C	-5.748388	-0.516495	-0.189046
H	-6.122165	-1.532791	-0.316509
H	-6.190315	-0.096475	0.719190
H	-6.110581	0.090830	-1.024099

4'-methoxy-2,4-dimethyl-1,2,3,6-tetrahydro-1,1'-biphenyl_oxidized

C	-1.960855	-1.560732	-0.131426
C	-3.458978	-1.576652	-0.185450
C	-4.221811	-0.490932	-0.127193
C	-3.625114	0.881289	0.023255
H	-1.555093	-2.263232	-0.863008
H	-1.618766	-1.912252	0.847817
H	-3.923170	-2.552691	-0.285854
H	-3.746332	1.426449	-0.921425
H	-4.202420	1.444993	0.762461
C	-2.155490	0.894077	0.434746
C	-1.397639	-0.152661	-0.410771
H	-2.096903	0.585165	1.483618
H	-1.579727	0.081158	-1.463462
C	-1.566434	2.292441	0.296643
H	-2.173130	3.015916	0.843184
H	-0.549140	2.349127	0.688987
H	-1.542779	2.598937	-0.752359
C	0.072882	-0.136539	-0.179773
C	0.967751	0.058778	-1.262918
C	0.608304	-0.327865	1.120592
C	2.315861	0.069918	-1.084621
H	0.558340	0.204795	-2.253628
C	1.947564	-0.320096	1.328938
H	-0.061891	-0.475884	1.956346
C	2.833723	-0.122541	0.225717
H	2.980580	0.223464	-1.921085
H	2.380052	-0.459707	2.309918
O	4.096032	-0.134562	0.517182
C	5.103922	0.043052	-0.495501
H	4.983744	1.015412	-0.969533
H	6.048055	-0.005477	0.034534
H	5.036213	-0.760952	-1.226160
C	-5.716061	-0.547236	-0.220092
H	-6.073597	-1.565609	-0.371239
H	-6.174622	-0.149443	0.689327
H	-6.072478	0.070705	-1.049260

down 4''-methoxy-3'-methyl-1',2',3',4'-tetrahydro-1,1':2',1''-terphenyl

C	-1.983743	-1.478287	-0.038614
C	-3.480649	-1.481293	-0.171527
C	-4.222950	-0.383170	-0.160376
C	-3.660087	0.995793	-0.008461
H	-1.579580	-2.123399	-0.824895
H	-3.954308	-2.453016	-0.268643
H	-5.298867	-0.466326	-0.276787
H	-3.751675	1.522458	-0.967259
H	-4.271622	1.566347	0.696610
C	-2.197145	1.026098	0.441336
C	-1.406206	-0.055791	-0.309726
H	-2.168179	0.797589	1.511592
H	-1.577539	0.125310	-1.377293
C	-1.623663	2.423705	0.233300
H	-2.269196	3.173043	0.696545
H	-0.626648	2.525876	0.660793
H	-1.555954	2.653450	-0.833919
C	0.088783	-0.013671	-0.094892
C	0.951181	-0.216611	-1.159814
C	0.659985	0.175403	1.165055

C	2.333622	-0.239694	-1.000867
H	0.541573	-0.365267	-2.153110
C	2.026846	0.162530	1.346340
H	0.022873	0.327186	2.027799
C	2.878140	-0.048922	0.261998
H	2.962534	-0.401522	-1.864535
H	2.458019	0.309766	2.328428
O	4.205901	-0.048429	0.535512
C	5.105543	-0.260453	-0.531980
H	5.013641	0.520438	-1.292299
H	6.102860	-0.224892	-0.100504
H	4.948623	-1.237708	-0.997470
C	-1.536025	-2.083325	1.279629
C	-0.419697	-2.914597	1.318103
C	-2.188571	-1.802241	2.476313
C	0.045708	-3.434780	2.515215
H	0.102305	-3.142412	0.395770
C	-1.724001	-2.315143	3.679211
H	-3.074351	-1.179189	2.467511
C	-0.602737	-3.131052	3.703933
H	0.918934	-4.075304	2.520561
H	-2.244160	-2.079889	4.599636
H	-0.240880	-3.533557	4.641714

down 4''-methoxy-3'-methyl-1',2',3',4'-tetrahydro-1,1'2',1''-terphenyl_oxidized

C	-1.968633	-1.469790	-0.060046
C	-3.465902	-1.475314	-0.177670
C	-4.218819	-0.385570	-0.159739
C	-3.673206	1.000194	-0.012590
H	-1.569430	-2.099626	-0.860087
H	-3.924834	-2.453347	-0.273585
H	-5.293673	-0.481807	-0.267334
H	-3.769676	1.524968	-0.970989
H	-4.287987	1.563797	0.693733
C	-2.215168	1.044966	0.444764
C	-1.421025	-0.021173	-0.326691
H	-2.189519	0.800409	1.510331
H	-1.584997	0.158450	-1.393888
C	-1.644760	2.446080	0.249931
H	-2.289483	3.185517	0.727162
H	-0.647441	2.552809	0.679102
H	-1.582678	2.691265	-0.813277
C	0.051842	0.001033	-0.096299
C	0.927251	-0.269002	-1.179844
C	0.609775	0.224631	1.189261
C	2.276253	-0.312619	-1.018142
H	0.500567	-0.443887	-2.158990
C	1.951093	0.192621	1.379739
H	-0.044723	0.414789	2.027717
C	2.816608	-0.075136	0.276727
H	2.924284	-0.519119	-1.856464
H	2.398717	0.357875	2.349723
O	4.082583	-0.081156	0.551814
C	5.074322	-0.331538	-0.461570
H	5.017596	0.437749	-1.229797
H	6.025185	-0.279761	0.056052
H	4.925992	-1.323261	-0.884818
C	-1.489190	-2.070286	1.250097
C	-0.372069	-2.901364	1.262789
C	-2.115258	-1.782122	2.459039
C	0.124977	-3.410509	2.452440
H	0.118987	-3.150700	0.328631
C	-1.618168	-2.285090	3.652344
H	-3.006432	-1.167323	2.467954
C	-0.493401	-3.096787	3.653826
H	0.994353	-4.055654	2.440350
H	-2.117016	-2.047569	4.583419
H	-0.108260	-3.492592	4.584809

down 4'-methoxy-6-methyl-1,2,5,6-tetrahydro-[1,1'-biphenyl]-2-yl acetate

C	-2.340856	-1.411944	-0.377106
C	-3.824933	-1.355302	-0.560904
C	-4.511139	-0.222783	-0.504167
C	-3.886776	1.107528	-0.224431
H	-1.893313	-2.059056	-1.130839
H	-4.326021	-2.297879	-0.751056
H	-5.582385	-0.240862	-0.675981
H	-3.860425	1.689137	-1.155171
H	-4.526526	1.673937	0.457573
C	-2.473582	1.011924	0.352060
C	-1.671417	-0.028633	-0.444570
H	-2.558838	0.660961	1.385941
H	-1.731300	0.263751	-1.499537
C	-1.812817	2.385017	0.353218
H	-2.451685	3.115707	0.853418
H	-0.849653	2.373277	0.862722
H	-1.646161	2.730102	-0.671093
C	-0.205151	-0.119741	-0.089707
C	0.750886	-0.229428	-1.086780
C	0.239419	-0.144112	1.233226
C	2.104150	-0.370552	-0.800712
H	0.440096	-0.211335	-2.125471
C	1.577247	-0.283475	1.540516
H	-0.474117	-0.053913	2.042705
C	2.523321	-0.402483	0.523113
H	2.810172	-0.454563	-1.614257
H	1.911707	-0.301524	2.570045
O	3.811549	-0.538321	0.918418
C	4.804726	-0.668796	-0.077669
H	4.842749	0.215037	-0.720709

H	5.750587	-0.770131	0.448625
H	4.637116	-1.557085	-0.693106
O	-2.090750	-2.018042	0.912764
C	-1.237661	-3.045235	0.993956
O	-0.723731	-3.579203	0.043844
C	-0.999830	-3.429969	2.423713
H	-1.933755	-3.435424	2.983153
H	-0.519800	-4.403365	2.470366
H	-0.342293	-2.683673	2.873889

down 4'-methoxy-6-methyl-1,2,5,6-tetrahydro-[1,1'-biphenyl]-2-yl acetate_oxidized

C	-2.172815	-1.401075	-0.358556
C	-3.655539	-1.408043	-0.559403
C	-4.393271	-0.309846	-0.485089
C	-3.838254	1.045945	-0.184226
H	-1.692519	-2.036769	-1.102895
H	-4.103238	-2.370991	-0.773509
H	-5.460969	-0.375546	-0.663051
H	-3.855152	1.646051	-1.102440
H	-4.497897	1.562267	0.517024
C	-2.418807	1.019788	0.382100
C	-1.585403	0.024325	-0.440561
H	-2.477788	0.654245	1.411339
H	-1.667057	0.309629	-1.494129
C	-1.818197	2.420749	0.380272
H	-2.480307	3.115904	0.898197
H	-0.848056	2.457016	0.878020
H	-1.686501	2.781320	-0.643038
C	-0.130145	-0.035417	-0.103369
C	0.813216	-0.218608	-1.146852
C	0.332946	0.025770	1.234476
C	2.144163	-0.335426	-0.896722
H	0.455558	-0.267510	-2.167003
C	1.655150	-0.084306	1.514994
H	-0.376370	0.157206	2.038193
C	2.592400	-0.268695	0.452371
H	2.845963	-0.475594	-1.705075
H	2.035396	-0.041599	2.526098
O	3.830613	-0.366569	0.815951
C	4.887505	-0.561836	-0.143313
H	4.912901	0.275415	-0.838375
H	5.797697	-0.594344	0.444095
H	4.737906	-1.505049	-0.665647
O	-1.847604	-1.929631	0.944434
C	-1.646854	-3.251796	1.054685
O	-1.740987	-4.014740	0.128981
C	-1.293682	-3.623606	2.461895
H	-2.100459	-3.326124	3.132051
H	-1.130352	-4.694907	2.530057
H	-0.394380	-3.088054	2.768022

up 4''-methoxy-3'-methyl-1',2',3',4'-tetrahydro-1,1':2',1''-terphenyl

C	-2.039453	-1.542993	-0.170313
C	-3.544410	-1.514497	-0.214765
C	-4.263130	-0.401767	-0.164266
C	-3.657677	0.961171	-0.029761
H	-1.740067	-1.863592	0.834293
H	-4.042766	-2.475333	-0.295753
H	-5.345319	-0.463605	-0.216552
H	-3.746665	1.494196	-0.985176
H	-4.234866	1.549564	0.689270
C	-2.190805	0.927589	0.398572
C	-1.435384	-0.138306	-0.413241
H	-2.158041	0.632287	1.453884
H	-1.589133	0.099709	-1.472503
C	-1.566506	2.310848	0.263367
H	-2.168012	3.055754	0.788655
H	-0.556243	2.340580	0.671846
H	-1.512041	2.604851	-0.788798
C	0.053273	-0.148798	-0.161252
C	0.956470	0.007453	-1.198763
C	0.571215	-0.350300	1.118911
C	2.331999	-0.033866	-0.992954
H	0.585799	0.158118	-2.206350
C	1.930978	-0.392572	1.347589
H	-0.101681	-0.477729	1.959427
C	2.825676	-0.235913	0.288938
H	2.996279	0.089257	-1.836286
H	2.323246	-0.548282	2.344606
O	4.142212	-0.295527	0.606226
C	5.084859	-0.148265	-0.434933
H	4.994883	0.828345	-0.919076
H	6.064850	-0.229585	0.028791
H	4.974700	-0.935351	-1.186307
C	-1.463236	-2.545617	-1.147325
C	-0.460203	-3.425815	-0.757232
C	-1.890678	-2.572025	-2.472071
C	0.105898	-4.309530	-1.665140
H	-0.110835	-3.411225	0.268767
C	-1.329050	-3.452688	-3.383625
H	-2.673244	-1.893178	-2.792510
C	-0.326625	-4.325459	-2.982732
H	0.887517	-4.985805	-1.341684
H	-1.675163	-3.458848	-4.409899
H	0.112842	-5.014326	-3.693145

up 4''-methoxy-3'-methyl-1',2',3',4'-tetrahydro-1,1'2',1''-terphenyl_oxidized

C	-2.019922	-1.527392	-0.163884
C	-3.523366	-1.524321	-0.233411

C	-4.265809	-0.429366	-0.162219
C	-3.698354	0.944099	0.019887
H	-1.724435	-1.841865	0.843157
H	-3.993156	-2.494598	-0.352829
H	-5.344389	-0.513329	-0.236388
H	-3.804077	1.507930	-0.914873
H	-4.288181	1.488891	0.761470
C	-2.233550	0.940729	0.452728
C	-1.468137	-0.089123	-0.398893
H	-2.196037	0.618104	1.498189
H	-1.641422	0.154264	-1.451420
C	-1.633434	2.337342	0.342895
H	-2.245767	3.057122	0.887939
H	-0.623140	2.383435	0.753478
H	-1.589550	2.656538	-0.701602
C	0.003833	-0.103272	-0.164796
C	0.899481	-0.082123	-1.265213
C	0.533974	-0.209660	1.145411
C	2.245231	-0.168937	-1.094736
H	0.490080	-0.007351	-2.263755
C	1.871947	-0.295523	1.346670
H	-0.136516	-0.225557	1.993839
C	2.759179	-0.281252	0.226268
H	2.908568	-0.157594	-1.946455
H	2.302805	-0.379466	2.334701
O	4.017725	-0.378156	0.516453
C	5.029026	-0.386056	-0.508295
H	4.980175	0.538024	-1.081030
H	5.970272	-0.451915	0.024959
H	4.893892	-1.255085	-1.149757
C	-1.392153	-2.496874	-1.144436
C	-0.388391	-3.367201	-0.735301
C	-1.768146	-2.491380	-2.484669
C	0.233867	-4.211019	-1.644854
H	-0.087536	-3.384374	0.306226
C	-1.149722	-3.332507	-3.395917
H	-2.554423	-1.822940	-2.817252
C	-0.144145	-4.194224	-2.978518
H	1.012317	-4.884304	-1.308744
H	-1.454879	-3.317903	-4.434624
H	0.337735	-4.852885	-3.689669

up 4'-methoxy-6-methyl-1,2,5,6-tetrahydro-[1,1'-biphenyl]-2-ylacetate

C	-2.390597	-1.352775	-0.036874
C	-3.882295	-1.214907	-0.023750
C	-4.490939	-0.042016	0.074224
C	-3.755239	1.256655	0.189757
H	-2.049540	-1.759520	0.917454
H	-4.451894	-2.135284	-0.087457
H	-5.575071	-0.002436	0.072164
H	-3.860757	1.810409	-0.751763
H	-4.229502	1.880517	0.952339
C	-2.270903	1.086683	0.517863
C	-1.669449	-0.041209	-0.340859
H	-2.193255	0.786096	1.569339
H	-1.874783	0.202809	-1.389080
C	-1.529283	2.404830	0.338716
H	-2.019906	3.198031	0.906242
H	-0.495203	2.334965	0.676281
H	-1.520722	2.700070	-0.714244
C	-0.175324	-0.193991	-0.182305
C	0.678008	-0.028863	-1.260005
C	0.396064	-0.503188	1.053088
C	2.057344	-0.161305	-1.135205
H	0.264500	0.210440	-2.233080
C	1.760868	-0.638594	1.200309
H	-0.235766	-0.637636	1.923815
C	2.605590	-0.468133	0.103304
H	2.682135	-0.024154	-2.006043
H	2.195439	-0.877892	2.162488
O	3.930361	-0.620607	0.341258
C	4.825137	-0.456655	-0.739302
H	4.763847	0.551909	-1.157844
H	5.821518	-0.617410	-0.335235
H	4.634017	-1.188301	-1.529427
O	-2.004875	-2.287266	-1.066810
C	-1.880710	-3.575569	-0.739648
O	-2.114939	-4.015748	0.358378
C	-1.405407	-4.384774	-1.909469
H	-0.402869	-4.057934	-2.188432
H	-1.388954	-5.438631	-1.647498
H	-2.057669	-4.220302	-2.766784

up 4'-methoxy-6-methyl-1,2,5,6-tetrahydro-[1,1'-biphenyl]-2-ylacetate_oxidized

C	-2.367933	-1.364422	-0.035015
C	-3.858309	-1.218546	-0.041766
C	-4.464125	-0.045364	0.061778
C	-3.731151	1.251852	0.203059
H	-2.038802	-1.756669	0.929347
H	-4.425637	-2.137947	-0.124691
H	-5.547219	-0.002759	0.043936
H	-3.838956	1.826743	-0.724422
H	-4.201946	1.856111	0.982472
C	-2.248914	1.082105	0.533024
C	-1.664289	-0.041725	-0.355120
H	-2.170096	0.760667	1.576784
H	-1.884123	0.205132	-1.397160
C	-1.497173	2.396257	0.365598
H	-1.985928	3.184178	0.939911

H	-0.465002	2.324525	0.713328
H	-1.483439	2.703481	-0.682999
C	-0.185614	-0.184345	-0.205390
C	0.667010	0.014558	-1.320320
C	0.388617	-0.516926	1.046359
C	2.017432	-0.106668	-1.217354
H	0.223514	0.266305	-2.274217
C	1.731814	-0.644382	1.180471
H	-0.248397	-0.663859	1.907806
C	2.578552	-0.440828	0.046706
H	2.650677	0.044402	-2.078627
H	2.196774	-0.892816	2.124258
O	3.845441	-0.579965	0.270579
C	4.823781	-0.394360	-0.770665
H	4.744698	0.613064	-1.174652
H	5.782029	-0.534027	-0.284052
H	4.678668	-1.144075	-1.546508
O	-1.955248	-2.297744	-1.050296
C	-1.875346	-3.592979	-0.708773
O	-2.134198	-4.003717	0.392884
C	-1.424263	-4.448873	-1.853201
H	-0.504515	-4.957708	-1.564656
H	-2.180520	-5.211804	-2.036157
H	-1.258885	-3.868558	-2.756359

4'-methoxy-2,4,5-trimethyl-1,2,3,6-tetrahydro-1,1'-biphenyl

C	-2.258284	-0.628101	-1.386030
C	-2.818956	-1.604613	-0.379486
C	-2.831524	-1.343026	0.929517
C	-2.283409	-0.050003	1.483623
H	-3.092902	-0.228429	-1.973299
H	-1.643086	-1.178293	-2.105794
H	-2.924894	0.281949	2.306634
H	-1.305648	-0.254496	1.936693
C	-2.154944	1.070908	0.456932
C	-1.452091	0.533561	-0.804624
H	-1.522033	1.854771	0.882694
H	-1.449026	1.339404	-1.544575
C	-3.510541	1.692992	0.129710
H	-3.937389	2.166146	1.016255
H	-3.414226	2.455731	-0.646322
H	-4.222432	0.942526	-0.219688
C	0.000394	0.203893	-0.520480
C	0.933289	1.231674	-0.473566
C	0.462883	-1.087523	-0.269572
C	2.273871	1.005971	-0.187649
H	0.610480	2.248681	-0.669908
C	1.792201	-1.335622	0.017758
H	-0.228257	-1.920742	-0.288197
C	2.710410	-0.290009	0.062642
H	2.958965	1.841534	-0.169214
H	2.137883	-2.343282	0.211604
O	3.992188	-0.627357	0.350532
C	4.955269	0.403607	0.407193
H	4.711794	1.135109	1.183098
H	5.900026	-0.076141	0.650973
H	5.048945	0.915717	-0.554732
C	-3.338060	-2.275312	1.992773
H	-4.221931	-1.854430	2.481785
H	-3.593763	-3.266308	1.625550
H	-2.579080	-2.394416	2.771989
C	-3.348628	-2.857203	-1.018171
H	-2.529942	-3.440998	-1.451141
H	-3.897980	-3.501009	-0.335681
H	-4.017376	-2.599983	-1.845000

4'-methoxy-2,4,5-trimethyl-1,2,3,6-tetrahydro-1,1'-biphenyl_oxidized

C	-2.278993	-0.640292	-1.405807
C	-2.806442	-1.610001	-0.376377
C	-2.774512	-1.342879	0.931715
C	-2.229351	-0.041119	1.469162
H	-3.123897	-0.224425	-1.963601
H	-1.696490	-1.190797	-2.151885
H	-2.875198	0.298205	2.284522
H	-1.255049	-0.233172	1.935413
C	-2.114004	1.076464	0.438128
C	-1.451747	0.516315	-0.853701
H	-1.467774	1.858993	0.843086
H	-1.442074	1.331114	-1.584062
C	-3.467135	1.706524	0.121707
H	-3.865597	2.190659	1.014461
H	-3.378053	2.461359	-0.661415
H	-4.192201	0.958679	-0.202429
C	-0.023965	0.189428	-0.573129
C	0.907924	1.262261	-0.485509
C	0.440175	-1.129222	-0.352797
C	2.216287	1.056270	-0.187431
H	0.554481	2.269612	-0.664895
C	1.742536	-1.364204	-0.048162
H	-0.254466	-1.954759	-0.410912
C	2.659436	-0.274208	0.046855
H	2.905937	1.884747	-0.129976
H	2.119293	-2.361459	0.132221
O	3.876948	-0.596774	0.353966
C	4.905520	0.400060	0.496832
H	4.648695	1.079664	1.307843
H	5.804463	-0.154832	0.739110
H	5.032602	0.937934	-0.440789
C	-3.245258	-2.272889	2.012385
H	-4.142202	-1.873741	2.494790

H	-3.466733	-3.277442	1.661408
H	-2.481150	-2.351660	2.791038
C	-3.361000	-2.862453	-0.992506
H	-2.562002	-3.451657	-1.453281
H	-3.887933	-3.499429	-0.287255
H	-4.058877	-2.603993	-1.793898

4'-methoxy-2,4,6,6-tetramethyl-1,2,3,6-tetrahydro-1,1'-biphenyl

C	2.204649	0.553476	-1.476925
C	2.854517	1.559383	-0.559361
C	2.961436	1.469429	0.762724
C	2.406539	0.300559	1.528762
H	3.271576	2.432886	-1.057573
H	3.102226	0.034940	2.331011
H	1.489279	0.618332	2.036383
C	2.146550	-0.929100	0.658247
C	1.445532	-0.544131	-0.670715
H	1.440826	-1.574353	1.188370
H	1.450484	-1.441302	-1.296637
C	3.434299	-1.736724	0.487506
H	3.695869	-2.214237	1.434073
H	3.323036	-2.519921	-0.264974
H	4.275200	-1.103849	0.200855
C	-0.018076	-0.248822	-0.396484
C	-0.977644	-1.196758	-0.719207
C	-0.468938	0.924532	0.215198
C	-2.330797	-1.011714	-0.451989
H	-0.669698	-2.119536	-1.198597
C	-1.805751	1.131147	0.488134
H	0.237369	1.705951	0.465057
C	-2.751548	0.161411	0.158937
H	-3.033818	-1.784804	-0.727685
H	-2.139419	2.048347	0.956984
O	-4.040041	0.452209	0.465749
C	-5.031177	-0.500665	0.144549
H	-4.859906	-1.446038	0.667305
H	-5.977653	-0.076849	0.471186
H	-5.071932	-0.686280	-0.932613
C	3.290948	-0.093244	-2.358680
H	3.842642	0.679919	-2.898316
H	4.008110	-0.672431	-1.782945
H	2.832369	-0.755129	-3.097868
C	1.269088	1.296603	-2.445678
H	0.735565	0.587341	-3.083154
H	0.529926	1.906313	-1.929313
H	1.856714	1.953943	-3.090677
C	3.609030	2.541202	1.587618
H	4.473335	2.144619	2.128157
H	3.939411	3.380597	0.975249
H	2.912651	2.918477	2.342811

4'-methoxy-2,4,6,6-tetramethyl-1,2,3,6-tetrahydro-1,1'-biphenyl_oxidized

C	2.215384	0.577326	-1.495106
C	2.831054	1.575756	-0.548718
C	2.911596	1.467529	0.773875
C	2.346359	0.288691	1.517205
H	3.253752	2.455627	-1.028095
H	3.035206	0.008507	2.318942
H	1.430368	0.602740	2.030465
C	2.093796	-0.937438	0.640712
C	1.444037	-0.537782	-0.726718
H	1.370115	-1.578533	1.150879
H	1.460296	-1.431737	-1.353460
C	3.370013	-1.762964	0.479974
H	3.603291	-2.247698	1.429171
H	3.258979	-2.540066	-0.277453
H	4.222098	-1.137860	0.213885
C	0.004732	-0.244456	-0.449816
C	-0.962674	-1.251125	-0.716374
C	-0.435831	0.973964	0.132379
C	-2.281464	-1.076842	-0.437627
H	-0.631756	-2.182231	-1.157293
C	-1.744623	1.174537	0.423409
H	0.285303	1.753620	0.333171
C	-2.699827	0.151141	0.144451
H	-2.996565	-1.855858	-0.654728
H	-2.100239	2.095821	0.863417
O	-3.925462	0.433269	0.457397
C	-4.994726	-0.503948	0.233075
H	-4.816138	-1.411323	0.807266
H	-5.888204	-0.002537	0.586621
H	-5.076926	-0.720922	-0.830436
C	3.325984	-0.064201	-2.348114
H	3.880609	0.717139	-2.870769
H	4.034835	-0.634064	-1.753972
H	2.893097	-0.728165	-3.099910
C	1.292985	1.309556	-2.486619
H	0.789740	0.595124	-3.142822
H	0.534151	1.918257	-1.996089
H	1.887636	1.975810	-3.113555
C	3.539593	2.528475	1.626119
H	4.396253	2.124801	2.172606
H	3.876344	3.377878	1.032276
H	2.829604	2.889913	2.375852

4'-methoxy-2,4-dimethyl-1,2,3,6-tetrahydro-1,1'-biphenyl

C	-2.244827	-0.616641	-1.408942
C	-2.801168	-1.573048	-0.394398
C	-2.826816	-1.346520	0.915411
C	-2.271358	-0.074705	1.499562

H	-3.071802	-0.208622	-2.000179
H	-1.621020	-1.160914	-2.123870
H	-3.212063	-2.502501	-0.779029
H	-2.907186	0.250623	2.329027
H	-1.292921	-0.294214	1.943378
C	-2.142072	1.055844	0.478210
C	-1.444038	0.540679	-0.799071
H	-1.505739	1.833605	0.909661
H	-1.448035	1.362897	-1.520326
C	-3.497873	1.684857	0.164725
H	-3.924793	2.140113	1.060544
H	-3.400702	2.463182	-0.595413
H	-4.209881	0.942218	-0.200833
C	0.011976	0.216659	-0.525901
C	0.939473	1.249615	-0.483174
C	0.483086	-1.072300	-0.278272
C	2.282863	1.031226	-0.204792
H	0.609980	2.264974	-0.676847
C	1.815367	-1.313069	0.001421
H	-0.203387	-1.909354	-0.294878
C	2.728087	-0.262478	0.041897
H	2.963117	1.870816	-0.189513
H	2.167638	-2.319014	0.192261
O	4.013242	-0.593127	0.322049
C	4.971174	0.442821	0.374790
H	4.727493	1.172553	1.152275
H	5.919523	-0.032148	0.613915
H	5.057809	0.955863	-0.587311
C	-3.373376	-2.338931	1.896200
H	-4.214267	-1.913074	2.451312
H	-3.710715	-3.251663	1.404098
H	-2.612689	-2.607334	2.635736

4'-methoxy-2,4-dimethyl-1,2,3,6-tetrahydro-1,1'-biphenyl_oxidized

C	-2.259326	-0.634064	-1.419187
C	-2.784650	-1.583167	-0.382989
C	-2.782350	-1.345453	0.925441
C	-2.235006	-0.062258	1.493187
H	-3.095804	-0.212731	-1.984729
H	-1.663798	-1.178491	-2.157421
H	-3.195267	-2.517589	-0.752356
H	-2.882623	0.274381	2.307697
H	-1.264827	-0.269319	1.960756
C	-2.108225	1.061592	0.465312
C	-1.442110	0.521112	-0.836930
H	-1.460983	1.838338	0.879482
H	-1.436702	1.350839	-1.549654
C	-3.458487	1.698166	0.148774
H	-3.864482	2.168985	1.045182
H	-3.361741	2.464547	-0.622005
H	-4.180978	0.955993	-0.193861
C	-0.011815	0.201218	-0.560110
C	0.912377	1.280543	-0.470356
C	0.462557	-1.114719	-0.346321
C	2.224590	1.083082	-0.183715
H	0.549567	2.285887	-0.642113
C	1.769135	-1.341524	-0.053457
H	-0.226472	-1.945152	-0.404088
C	2.680481	-0.245969	0.034127
H	2.906734	1.917662	-0.125751
H	2.154659	-2.336856	0.118913
O	3.905731	-0.563453	0.313900
C	4.933954	0.437381	0.427067
H	4.685171	1.133163	1.226435
H	5.836636	-0.111022	0.670239
H	5.049026	0.955455	-0.523428
C	-3.300979	-2.332784	1.925152
H	-4.142101	-1.910772	2.481920
H	-3.629532	-3.256350	1.448885
H	-2.528260	-2.577784	2.659727

down 4''-methoxy-3'-methyl-1',2',3',4'-tetrahydro-1,1':2',1''-terphenyl

C	-2.178651	-0.687358	-1.359019
C	-2.780754	-1.639826	-0.362208
C	-2.930703	-1.362348	0.926924
C	-2.459439	-0.087001	1.558762
H	-3.022658	-0.208718	-1.870535
H	-3.152565	-2.581931	-0.752425
H	-3.403642	-2.093127	1.574722
H	-3.205541	0.263147	2.277322
H	-1.559011	-0.292244	2.148122
C	-2.184295	1.013184	0.529788
C	-1.393003	0.466966	-0.679474
H	-1.558584	1.775713	1.001446
H	-1.348103	1.275380	-1.414235
C	-3.484627	1.685373	0.091015
H	-3.951258	2.197138	0.934728
H	-3.300633	2.424430	-0.691756
H	-4.204839	0.958605	-0.290861
C	0.046854	0.151519	-0.330748
C	1.046272	1.022278	-0.740743
C	0.444747	-0.968399	0.403949
C	2.387975	0.813888	-0.440101
H	0.779386	1.898238	-1.321627
C	1.770556	-1.196987	0.711776
H	-0.292420	-1.689921	0.729002
C	2.756690	-0.305653	0.293568
H	3.123835	1.524542	-0.788224
H	2.063720	-2.072820	1.276872
O	4.030227	-0.613390	0.641486

C	5.061780	0.257345	0.227109
H	4.930007	1.259442	0.645167
H	5.988640	-0.168794	0.603175
H	5.112135	0.324941	-0.863355
C	-1.366704	-1.353664	-2.449335
C	-1.211388	-0.704635	-3.671984
C	-0.730094	-2.576432	-2.268081
C	-0.437135	-1.253882	-4.681449
H	-1.705821	0.247218	-3.833715
C	0.044171	-3.133374	-3.276358
H	-0.831719	-3.099625	-1.325472
C	0.196198	-2.473652	-4.486103
H	-0.331113	-0.731277	-5.624104
H	0.532889	-4.085924	-3.112732
H	0.800609	-2.907278	-5.272799

down 4''-methoxy-3'-methyl-1',2',3',4'-tetrahydro-1,1'2',1''-terphenyl_oxidized

C	-2.191507	-0.707557	-1.367515
C	-2.790947	-1.625906	-0.339793
C	-2.875698	-1.339368	0.952897
C	-2.344036	-0.075551	1.559051
H	-3.031961	-0.229431	-1.883231
H	-3.218317	-2.550771	-0.711095
H	-3.349217	-2.048543	1.622458
H	-3.044411	0.288902	2.314044
H	-1.419482	-0.295506	2.104965
C	-2.113835	1.025619	0.522311
C	-1.391467	0.461957	-0.737995
H	-1.464381	1.787486	0.960172
H	-1.369496	1.270813	-1.470500
C	-3.423265	1.702562	0.123734
H	-3.856102	2.206573	0.988805
H	-3.260822	2.448164	-0.656173
H	-4.155316	0.979376	-0.239249
C	0.033429	0.158232	-0.407466
C	1.015698	1.148710	-0.681079
C	0.447347	-1.050492	0.207277
C	2.329029	0.962645	-0.386950
H	0.701320	2.074348	-1.145395
C	1.751077	-1.262907	0.514880
H	-0.286938	-1.810235	0.430558
C	2.724114	-0.260478	0.222157
H	3.056086	1.727967	-0.613807
H	2.089004	-2.177773	0.981202
O	3.942100	-0.556669	0.550178
C	5.031421	0.355521	0.317647
H	4.875312	1.268934	0.888896
H	5.914241	-0.164436	0.671204
H	5.115907	0.565733	-0.746895
C	-1.384095	-1.405528	-2.449165
C	-1.109616	-0.720890	-3.632032
C	-0.876395	-2.690651	-2.297512
C	-0.341455	-1.298137	-4.629089
H	-1.509247	0.276878	-3.776674
C	-0.107604	-3.274527	-3.295801
H	-1.079461	-3.250875	-1.393446
C	0.165422	-2.580282	-4.462958
H	-0.143815	-0.749672	-5.541439
H	0.276406	-4.277394	-3.157518
H	0.762964	-3.035758	-5.242165

down 4'-methoxy-6-methyl-1,2,5,6-tetrahydro-[1,1'-biphenyl]-2-yl acetate

C	-2.156658	-0.397945	-1.559543
C	-2.733209	-1.452555	-0.658480
C	-2.837111	-1.296290	0.653969
C	-2.331045	-0.087364	1.379794
H	-2.975522	0.139062	-2.041615
H	-3.098213	-2.355616	-1.135765
H	-3.280372	-2.087827	1.248436
H	-3.040854	0.200188	2.159450
H	-1.408706	-0.362772	1.903297
C	-2.077268	1.099313	0.445997
C	-1.313109	0.646386	-0.820928
H	-1.427935	1.810094	0.962394
H	-1.245286	1.514334	-1.481880
C	-3.379794	1.823031	0.104871
H	-3.806183	2.274521	1.002107
H	-3.208062	2.618211	-0.623486
H	-4.129231	1.142319	-0.305022
C	0.112569	0.245613	-0.496926
C	1.093723	1.226440	-0.529840
C	0.505936	-1.037803	-0.116340
C	2.417043	0.966442	-0.194408
H	0.826164	2.234232	-0.828564
C	1.815849	-1.319112	0.217399
H	-0.218888	-1.839804	-0.086236
C	2.784719	-0.318480	0.184219
H	3.140923	1.767368	-0.240370
H	2.109143	-2.320780	0.505114
O	4.041890	-0.689009	0.528162
C	5.057517	0.291853	0.497109
H	4.844615	1.108508	1.192808
H	5.972955	-0.209221	0.801902
H	5.187421	0.699257	-0.509486
O	-1.346418	-0.998481	-2.584847
C	-1.844824	-1.408867	-3.756969
O	-1.095089	-1.942708	-4.534144
C	-3.299677	-1.168392	-4.059585
H	-3.533667	-1.649147	-5.004352
H	-3.948404	-1.563945	-3.278566

H -3.495348 -0.098352 -4.147575

down 4'-methoxy-6-methyl-1,2,5,6-tetrahydro-[1,1'-biphenyl]-2-yl acetate_oxidized

C -2.168810 -0.410020 -1.565802
C -2.722634 -1.445916 -0.630852
C -2.781331 -1.272533 0.682214
C -2.265670 -0.052237 1.381742
H -2.990829 0.120769 -2.048073
H -3.103354 -2.353926 -1.084351
H -3.203972 -2.056000 1.300724
H -2.969367 0.247539 2.161396
H -1.341636 -0.316176 1.908546
C -2.030988 1.127027 0.434339
C -1.313897 0.647504 -0.864530
H -1.367933 1.842444 0.925168
H -1.245968 1.512121 -1.529211
C -3.333230 1.853427 0.102945
H -3.736211 2.311782 1.006567
H -3.169359 2.642617 -0.632144
H -4.091679 1.171732 -0.285802
C 0.092630 0.247023 -0.546376
C 1.057221 1.287954 -0.444177
C 0.504403 -1.083844 -0.294875
C 2.351766 1.041586 -0.117435
H 0.744889 2.305721 -0.639511
C 1.791570 -1.359079 0.036596
H -0.210722 -1.888727 -0.367448
C 2.746269 -0.301839 0.132118
H 3.066462 1.847905 -0.053148
H 2.129257 -2.367272 0.232163
O 3.947444 -0.663992 0.452425
C 5.013432 0.294953 0.587726
H 4.769043 1.007617 1.373307
H 5.883914 -0.288804 0.863519
H 5.178846 0.797671 -0.363467
O -1.353065 -1.018333 -2.577704
C -1.843763 -1.458096 -3.751813
O -1.078802 -1.993463 -4.507796
C -3.298513 -1.237832 -4.057462
H -3.526977 -1.734043 -4.995348
H -3.942614 -1.631176 -3.271452
H -3.506232 -0.171381 -4.160618

up 4''-methoxy-3'-methyl-1',2',3',4'-tetrahydro-1,1':2',1''-terphenyl

C -2.361494 -0.559839 -1.263171
C -2.926723 -1.565976 -0.300772
C -2.882567 -1.447040 1.019678
C -2.278569 -0.269133 1.721707
H -1.636692 -1.098736 -1.883944
H -3.376601 -2.449667 -0.742367
H -3.283924 -2.244457 1.636452
H -2.867491 -0.024716 2.609502
H -1.284793 -0.548595 2.089929
C -2.177174 0.957295 0.813584
C -1.568791 0.573306 -0.554351
H -1.482065 1.664175 1.274757
H -1.636348 1.460932 -1.186977
C -3.525142 1.663337 0.679634
H -3.829543 2.077381 1.643042
H -3.470190 2.483122 -0.039622
H -4.308392 0.982135 0.347599
C -0.090775 0.266493 -0.411516
C 0.830354 1.301059 -0.502121
C 0.407592 -1.010805 -0.152990
C 2.195625 1.095142 -0.344460
H 0.478854 2.306787 -0.706140
C 1.760913 -1.238447 0.007349
H -0.273236 -1.849173 -0.069728
C 2.668460 -0.185966 -0.086181
H 2.870102 1.935019 -0.430727
H 2.134768 -2.234734 0.207119
O 3.975443 -0.503780 0.082823
C 4.929928 0.533488 -0.002537
H 4.759031 1.296741 0.761988
H 5.898670 0.069222 0.164921
H 4.921494 1.003307 -0.990208
C -3.394651 -0.024527 -2.245291
C -4.762983 -0.186483 -2.067668
C -2.954754 0.653323 -3.381699
C -5.668529 0.322737 -2.989790
H -5.131431 -0.710194 -1.194205
C -3.852230 1.164715 -4.303905
H -1.889831 0.779675 -3.545417
C -5.218041 1.002113 -4.110001
H -6.730803 0.186978 -2.827941
H -3.486046 1.687507 -5.178864
H -5.922974 1.399570 -4.829230

up 4''-methoxy-3'-methyl-1',2',3',4'-tetrahydro-1,1'2',1''-terphenyl_oxidized

C -2.355139 -0.584016 -1.294873
C -2.894209 -1.575637 -0.304280
C -2.825991 -1.434100 1.012645
C -2.225944 -0.241152 1.692515
H -1.654411 -1.130349 -1.936445
H -3.345493 -2.467826 -0.724514
H -3.210561 -2.223190 1.649179
H -2.825629 0.023209 2.566385
H -1.239745 -0.512619 2.086430
C -2.122643 0.973867 0.770553

C	-1.548197	0.554627	-0.619603
H	-1.414927	1.680914	1.210156
H	-1.607240	1.442109	-1.253282
C	-3.460205	1.695530	0.629733
H	-3.740828	2.129917	1.590419
H	-3.401914	2.500886	-0.103927
H	-4.255094	1.017306	0.323443
C	-0.095916	0.252713	-0.454204
C	0.820915	1.341512	-0.444049
C	0.405979	-1.056222	-0.261874
C	2.154854	1.158056	-0.268956
H	0.435359	2.342859	-0.586831
C	1.734839	-1.269292	-0.080855
H	-0.276614	-1.894471	-0.248285
C	2.640946	-0.165481	-0.086209
H	2.830961	1.999724	-0.271575
H	2.142560	-2.259280	0.069958
O	3.888462	-0.471877	0.083557
C	4.917449	0.534908	0.097129
H	4.741899	1.229581	0.916358
H	5.841851	-0.008799	0.254163
H	4.939297	1.052509	-0.860318
C	-3.409237	-0.042025	-2.252301
C	-4.771780	-0.190749	-2.029438
C	-2.993803	0.620044	-3.406004
C	-5.698502	0.319887	-2.928609
H	-5.118804	-0.705722	-1.142284
C	-3.913934	1.133880	-4.304320
H	-1.933429	0.730405	-3.607323
C	-5.274038	0.986512	-4.066617
H	-6.756514	0.194982	-2.735313
H	-3.569839	1.644904	-5.194708
H	-5.996339	1.385260	-4.767361

up 4'-methoxy-6-methyl-1,2,5,6-tetrahydro-[1,1'-biphenyl]-2-ylacetate

C	-2.193124	-0.460477	-1.492869
C	-2.843443	-1.509803	-0.642077
C	-2.885867	-1.448768	0.682021
C	-2.318440	-0.313053	1.475452
H	-1.465985	-0.929385	-2.156265
H	-3.281169	-2.354611	-1.162878
H	-3.345843	-2.262822	1.231879
H	-2.956723	-0.116832	2.339955
H	-1.348284	-0.618574	1.883086
C	-2.167958	0.958696	0.639443
C	-1.468806	0.637980	-0.700414
H	-1.509817	1.644041	1.179200
H	-1.517547	1.535132	-1.320634
C	-3.509398	1.663267	0.444402
H	-3.912956	1.975121	1.409655
H	-3.394437	2.552203	-0.178408
H	-4.242419	1.015076	-0.036473
C	0.000012	0.326672	-0.490730
C	0.917638	1.368204	-0.480137
C	0.489052	-0.960624	-0.266878
C	2.272364	1.158128	-0.257663
H	0.571911	2.381431	-0.653258
C	1.833158	-1.192032	-0.045738
H	-0.188695	-1.805857	-0.258493
C	2.738090	-0.133369	-0.038418
H	2.945591	2.003251	-0.264198
H	2.200923	-2.195735	0.125474
O	4.034702	-0.454583	0.184725
C	4.987787	0.588201	0.200615
H	4.773053	1.312271	0.991534
H	5.948266	0.117991	0.396121
H	5.028803	1.105357	-0.762134
O	-3.202175	0.183740	-2.309337
C	-3.531212	-0.240781	-3.533331
O	-4.413956	0.338398	-4.114902
C	-2.779454	-1.396701	-4.137422
H	-2.784804	-2.270649	-3.486873
H	-3.248418	-1.647373	-5.083859
H	-1.739913	-1.118861	-4.318779

up 4'-methoxy-6-methyl-1,2,5,6-tetrahydro-[1,1'-biphenyl]-2-ylacetate_oxidized

C	-2.187591	-0.479476	-1.532738
C	-2.795062	-1.529084	-0.651344
C	-2.800916	-1.456620	0.672649
C	-2.233415	-0.308665	1.448014
H	-1.492688	-0.947954	-2.231875
H	-3.233587	-2.384305	-1.152120
H	-3.232821	-2.272021	1.241648
H	-2.873639	-0.100688	2.307564
H	-1.266746	-0.607405	1.869509
C	-2.092578	0.959398	0.605718
C	-1.433732	0.619342	-0.766922
H	-1.420630	1.645580	1.125987
H	-1.480091	1.521985	-1.379214
C	-3.429832	1.673219	0.422787
H	-3.799946	2.001846	1.394895
H	-3.322337	2.550736	-0.215583
H	-4.178397	1.021372	-0.025976
C	0.011352	0.312939	-0.545992
C	0.917140	1.405215	-0.437729
C	0.509318	-1.000888	-0.388981
C	2.239969	1.220230	-0.194507
H	0.533217	2.409701	-0.560496
C	1.827680	-1.216267	-0.141939

H	-0.164822	-1.844067	-0.451889	H	5.066520	1.149610	-0.596610
C	2.723606	-0.108055	-0.038816	O	-3.213364	0.188319	-2.290387
H	2.909598	2.064267	-0.124822	C	-3.622895	-0.226632	-3.501515
H	2.233278	-2.210036	-0.012507	O	-4.515407	0.383559	-4.026750
O	3.957754	-0.416008	0.200794	C	-2.939233	-1.403638	-4.140978
C	4.976159	0.594053	0.335120	H	-2.938347	-2.277100	-3.489841
H	4.732180	1.253076	1.166377	H	-3.465103	-1.640819	-5.060397
H	5.889134	0.047331	0.540406	H	-1.903219	-1.157780	-4.380149

References

1) Cismesia, M. A.; Yoon, T. P. Characterizing chain processes in visible light photoredox catalysis. *Chemical Science* **2015**, *6* (10), 5426-5434, 10.1039/C5SC02185E. DOI: 10.1039/C5SC02185E.

2) Lee, A. A.; Swierk, J. R. Mechanistic Investigation of a Photoredox Cycloaddition Chain Reaction. *Journal of the American Chemical Society* **2024**, *146* (50), 34900-34908. DOI: 10.1021/jacs.4c14255.
